# *Textrous!*: Extracting Semantic Textual Meaning from Gene Sets

**DOI:** 10.1371/journal.pone.0062665

**Published:** 2013-04-30

**Authors:** Hongyu Chen, Bronwen Martin, Caitlin M. Daimon, Sana Siddiqui, Louis M. Luttrell, Stuart Maudsley

**Affiliations:** 1 Receptor Pharmacology Unit, Laboratory of Neuroscience, National Institute on Aging, National Institutes of Health, Baltimore, Maryland, United States of America; 2 Metabolism Unit, Laboratory of Clinical Investigation, National Institute on Aging, National Institutes of Health, Baltimore, Maryland, United States of America; 3 Division of Endocrinology, Diabetes & Medical Genetics, Department of Medicine, Medical University of South Carolina, Charleston, South Carolina, United States of America; University of Vermont, United States of America

## Abstract

The un-biased and reproducible interpretation of high-content gene sets from large-scale genomic experiments is crucial to the understanding of biological themes, validation of experimental data, and the eventual development of plans for future experimentation. To derive biomedically-relevant information from simple gene lists, a mathematical association to scientific language and meaningful words or sentences is crucial. Unfortunately, existing software for deriving meaningful and easily-appreciable scientific textual ‘tokens’ from large gene sets either rely on controlled vocabularies (Medical Subject Headings, Gene Ontology, BioCarta) or employ Boolean text searching and co-occurrence models that are incapable of detecting indirect links in the literature. As an improvement to existing web-based informatic tools, we have developed *Textrous!*, a web-based framework for the extraction of biomedical semantic meaning from a given input gene set of arbitrary length. *Textrous!* employs natural language processing techniques, including latent semantic indexing (LSI), sentence splitting, word tokenization, parts-of-speech tagging, and noun-phrase chunking, to mine MEDLINE abstracts, PubMed Central articles, articles from the Online Mendelian Inheritance in Man (OMIM), and Mammalian Phenotype annotation obtained from Jackson Laboratories. *Textrous!* has the ability to generate meaningful output data with even very small input datasets, using two different text extraction methodologies (collective and individual) for the selecting, ranking, clustering, and visualization of English words obtained from the user data. *Textrous!*, therefore, is able to facilitate the output of quantitatively significant and easily appreciable semantic words and phrases linked to both individual gene and batch genomic data.

## Introduction

With the increasing experimental prevalence of high-throughput genomic technologies, researchers are often challenged with the task of selecting, analyzing, clustering, and interpreting lists of functionally-relevant genes to a particular experiment at hand [1]. Given that an abundance of information about individual genes is contained in the text of published literature, with the recent development of novel informatic procedures literature mining with natural language processing techniques has become much more fruitful in recent years [2]. Current developments in this emerging field include literature-based methods for determining the functional coherence of a gene set, generating related transcription factors from microarray derived gene sets, and the functional user-based clustering of related genes [3]–[5].

An important aspect of gene set interpretation is the transformation of large gene sets into interpretable and manageable forms. Bridging the gap between large gene sets and the English language is potentially valuable for a variety of applications, including the discovery of previously unknown biological connections, identification of potential research topics, visualization of biological themes, discrimination between specific data sets, and validation of existing data. Current software for the interpretation of high-throughput genomic data share one or more of the following characteristics: reliance on controlled-languages (Gene Ontology (GO), Medical Subject Headings (MeSH), BioCarta, Kyoto Encyclopedia of Genes and Genomes (KEGG)), inability to search more than a few genes, and use of standard Boolean and co-occurrence models [6]–[9]. For example, Gene2MeSH, LigerCat, AmiGO, and Genes2WordCloud, four tools for generating enriched biological themes from a gene set, employ Boolean models or use exclusively terms that are preselected by BioCarta, GO, MeSH, and KEGG [10]–[13]. Our development of *Textrous!* in no way makes any of these excellent resources redundant. Therefore *Textrous!* should, as with other applications, be seen as a complementary device that should be used in conjunction with other forms of textual analysis such as the exemplary LigerCat which facilitates data text extraction using ‘MeSH Cloud’ outputs [13]. The combined use of multiple data analysis tools is therefore likely to yield the most comprehensive and meaningful appreciation of the input data. Similar tools that fall into the same generic category include the Database for Annotation, Visualization, and Integrated Discovery (DAVID), PubMatrix, WebGestalt, and Gene Set Enrichment Analysis [14]–[17]. All of these important and useful applications can create structured text interpretations of complex biological data, but do so using rigid clustering criteria that may possess considerable redundancy or possess limitations in their scope.

The use of predefined vocabularies such as GO or KEGG pathways places limitations on the range of words that can be used to describe a gene set. In addition, the textual connections implied by these curated libraries may be added to (*e.g*. GO terms) or even rapidly superseded, *e.g*. for the case of KEGG pathways, by the acquisition of additional experimental knowledge. Potentially, this can lead to decreased recall, as infrequent words not suitable for curation are discarded completely. A co-occurrence model suffers from disadvantages as well; most notably, such a model is unable to extract indirect relationships and facilitate new discoveries. To address these issues, we have developed *Textrous!,* a web-based framework for the extraction of semantic meaning from gene sets without the use of controlled-languages and pathways. *Textrous!* employs various natural language processing techniques, including latent semantic indexing (LSI), sentence splitting, word tokenization, parts-of-speech tagging, and noun-phrase chunking, to mine MEDLINE abstracts, PubMed Central articles, articles from the Online Mendelian Inheritance in Man (OMIM), and Mammalian Phenotype annotation obtained from Jackson Laboratories (www.informatics.jax.org/phenotypes.shtml) [18], [19]. From an input of one or more genes, *Textrous!* is able to generate words and noun-phrases and their associated similarity scores, z-scores, and p-values. In addition, *Textrous!* can easily create a hierarchical cloud, combining elements of traditional word clouds and agglomerative hierarchical clustering, as well as a heat map, illustrating the pairwise similarities between each gene and word. *Textrous!* therefore presents an alternative to rigidly-curated data set interpretation systems that allows experimenters to generate additional and more nuanced levels of textual appreciation of large biomedical data sets.

## Materials and Methods

### Generation of “Gene-Documents”

Our corpus of “gene-documents” was created from a concatenation of all MEDLINE titles, abstracts, full articles, and articles from the Online Mendelian Inheritance in Man as well as all the articles from the Jackson Laboratories Mammalian Phenotype Database. Individual genes were linked to PubMed articles by manually curated citation cross-reference data in the Entrez Gene repository. High precision and low recall is expected due to the manual curation process, as there are far more gene-article links than curated links [3]. Since a small proportion of MEDLINE abstracts describe sequencing experiments that specifically mention a disproportionately large number of genes, all PubMed abstracts that mention more than ten genes are discarded. Abstracts and articles were downloaded using PubMed’s E-Utilities, and articles from OMIM and Jackson Laboratories were downloaded and extracted from their respective FTP dumps.

All gene-documents were kept in the collection and left unfiltered. Punctuation was stripped from all the gene-documents, with the exception of hyphens, underscores, and apostrophes. Words occurring in more than half of all documents, only one document, or found in Cornell University’s SMART stoplist were excluded from all documents [20], [21]. In addition to Cornell’s SMART stoplist, a small list of 200 words was manually added to the stoplist. These words were determined empirically by multiple experimenters in our laboratory after multiple diverse-user interrogations of *Textrous!*. The final corpus consisted of 67412 genes from a variety of plant and animal genomes and 12281 words.

### Generation of the Term Document Matrix

A term-document matrix was constructed by applying both local and global weightings to the frequency of terms across each document in the corpus. In the term-document matrix, each row represents an English word, while each column represents a gene in the gene-document collection. A term frequency (TF) - inverse document frequency (IDF) weighting scheme was used in the generation of the term-document matrix. Given our total collection of words (T), and gene-documents (D), term frequency can be calculated by the number of times a word appears in a document:
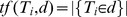



The global weighting function, inverse document frequency, can be calculated as follows:
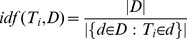



Weighting functions are applied to each element of the term-document matrix to increase the significance of words that are more likely to identify particular genes and decrease the significance of common, non-descriptive words. Since our corpus is selected from exclusively biological/biomedical literature, certain colloquial words have erroneously high global weightings (“*geared*”, “*ready*”, “*book*”) while other seemingly-important words often demonstrate inappropriately low weightings due to their relative commonality in biological/biomedical literature (“*energy*”, “*metabolism*”, “*cancer*”). To account for this, inverse document frequency was calculated from two sources: the “gene documents” collection from PubMed, OMIM, Jackson Laboratories, and the fifteenth edition of Encyclopedia Britannica (www.britannica.com/). The Encyclopedia Britannica text source was chosen specifically for its high-quality literary standards, large amount of text, academic writing, and variety of topics, some of which are not biomedically-related.

**Figure 1:**
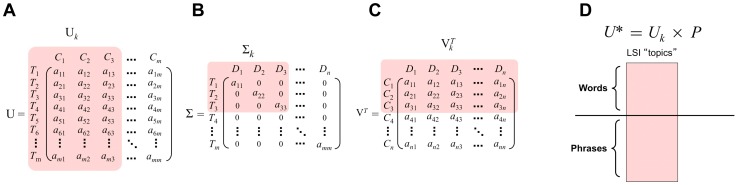
Singular Value Decomposition (SVD) on a term document matrix and the generation of the U* matrix. (A–C) U and V^T^ contain the LSI vectors for terms and documents, respectively while Σ contains the singular values of the original term document matrix. (D) An illustration of the resulting matrix U*, obtained by the multiplication of U_k_ and P. Note that the resulting matrix contains the word vectors and phrase vectors in LSI space, facilitating the comparison between every word/phrase and every other word/phrase entity.

A rank-reduced Singular Value Decomposition (SVD) was applied to the term-document matrix (M), yielding three matrices, U, Σ, and V^T^ (Figure 1A–C) [22]. Σ is computed by taking the square root of the eigenvalues of MM^T^ or M^T^M sorted in descending order on the main diagonal. U and V^T^ are computed by taking the eigenvectors of MM^T^ and M^T^M corresponding to the eigenvectors in Σ and placing them into their appropriate columns and rows, respectively. A rank of 120 was employed and was empirically determined by testing varying values of k (from 100 to 500). The columns of U and rows of V^T^ can be viewed as LSI “concepts” or “topics”, dimensions by which two terms or documents can be compared. As such, U and V^T^ are referred to as the “term-concept” and “document-concept” matrices, respectively.

### Generation of the Phrase Matrix

For each gene-document, all noun-phrases were extracted by the use of four statistically-based classifiers involved in sentence splitting, word tokenization, parts of speech tagging, and noun-phrase chunking. From this, a master list of all noun-phrases was obtained. This list was processed by eliminating all punctuation and capitalization with exception of hyphens, underscores, and apostrophes; stripping all preceding articles; removing duplicate phrases; and discarding all phrases that cannot be formed by the 12281 words in the term-document matrix. The resulting set of noun-phrases (NP) was used to generate a term-phrase-document matrix, with phrases and words as rows, gene documents as columns, and each cell as the presence of a word or phrase in a document.

A term-phrase-document matrix (P) was constructed as follows:
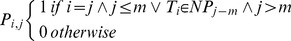



Weighting functions are not applied to avoid the application of weighting functions twice, because this matrix is left-multiplied by U_k_. The resulting matrix U* can be viewed as a “term-phrase-concept matrix”, with each row representing a word or phrase and each column representing an LSI dimension (Figure 1D) [23].

### Query Processing

Since each word and phrase is represented by a row vector in U*, a similarity score between any two words and phrases can be generated by using cosine distance. Cosine distance, defined by the cosine of the angle between two vectors, was computed as follows:
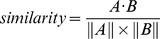



Higher cosine scores correspond to a higher degree of similarity between two words. A user query of a list of genes is then treated as another hypothetical word in the English language, represented by a row in the original term-document matrix. All values of this row vector are positive for genes in the user query, and zero otherwise. This, in effect, is the same as supposing such a word existed in all gene documents in the user query. Such a word would perfectly describe the user’s gene set and thus serves as a point-of-reference for all other input English words.

Fortunately, recomputation of the SVD is not needed to index the query into LSI space. Since M = USV^T^ and therefore MVS^−1^ =  U, we can index the query vector by right-multiplying it by V and S^−1^. The LSI-space query vector can then be compared to any other word or phrase in U* using cosine distance. The top associated words and phrases can then easily be retrieved by identifying the words and phrases with the highest associated cosine scores. Rudimentary statistical significance was calculated using a Student’s t-test.

### Generation of Hierarchical Word Clouds

Hierarchical clouds are an integration of agglomerative hierarchical clustering (typically viewed as a dendrogram) with traditional word clouds. Agglomerative hierarchical clustering was applied to the top 30 output words of any given gene-based query. Briefly, the process is defined as the initialization of each word as its own cluster and the two closest clusters at each step being iteratively joined into one at each step, forming a tree structure. Each join is represented by a 2×1 or 1×2 HTML table, chosen randomly. The resulting cloud displays the collection of words via nested HTML tables. Each cell is color-coded to represent the time at which joins were made and font sizes are adjusted to be proportional to the calculated cosine similarities.

### Generation of Gene-Word Heat Maps

Two-dimensional heat maps are generated to illustrate the pairwise differences between specific genes and specific words. Gene-word similarities were pre-calculated for all possible combinations and represented in a heat map format as an HTML table. In a heatmap, words are sorted by the number of genes with which they share statistical significance. The strength of gene-word association is indicated by the color intensity of each pairwise association. For the output heatmap teal is used as color of gene-word association. The most popular noun-phrase associations from the identified words can be accessed through a hyperlink embedded in the gene-word heat map.

### Programming Procedures

Generation of gene-documents, including word tokenization, web crawling, filtering and parsing, was written in Python. Construction of the term-document matrix was written in Java, and computation of the SVD was done with SVDLIBC [24]. The parsing of noun-phrases, generation of the phrase matrix, and indexing of the phrase matrix into U* was written in Java with the help of Apache OpenNLP [25]. All web development was programmed with Python CGI, and resulting data displayed with HTML and CSS.

### Accessing *Textrous!*

*Textrous!* is housed on a globally-visible NIH site at: http://textrous.irp.nia.nih.gov.

## Results

### Description of the *Textrous!* User Interface

**Figure 2:**
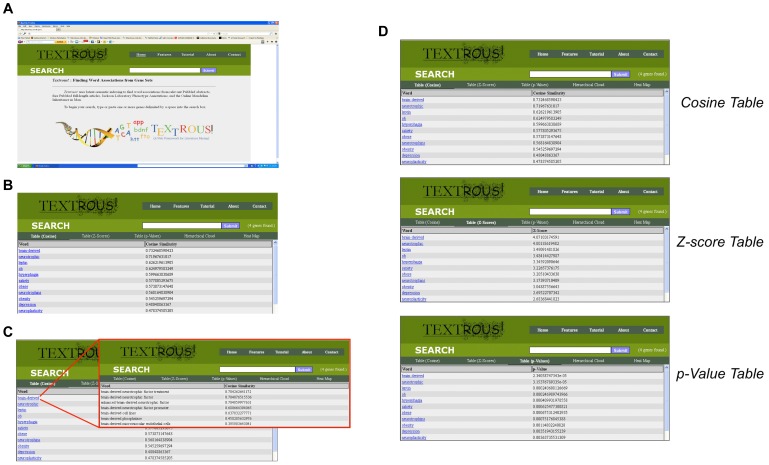
Web-based user interface for *Textrous!*. (A) The main navigation bar is on the top-right. The search bar is below the main navigation bar, and the secondary navigation bar is below the search bar. Features can be accessed by clicking the appropriate menu item, phrases by clicking on the word hyperlinks, and excluded words by clicking the “(x genes found)” description in the search bar. (B) Primary Cosine Similarity output from *Textrous!* user interface. The main navigation bar is on the top-right. The search bar is below the main navigation bar, and the secondary navigation bar is below the search bar. The ‘Cosine Similarity’ output is demonstrated for the following Gene Symbol input sequence: Lep, Bdnf, Fto, Lepr. After symbol input into the ‘Search’ box then the cosine similarity word list is generated by pressing ‘Submit’. Automatically the ‘Cosine Table’ is depicted first. Additional textual output modes can be accessed subsequently using the toolbar. (C) Phrase hyperlinking from Cosine Similarity tables. Each word term generated from the input query list can be clicked on to link out (in red box) to the phrases in which it resides. The phrases containing the identified word are ranked according to their cosine similarity as well. (D) In addition to the Cosine Similarity output feature, the resulting word lists can be assessed by their output Z-score table or the probability scores in their p-value table. In each of these text word output formats each word can be linked out to its phrase context scoring box as in Figure 5.

The current *Textrous!* website can be accessed at http://textrous.cit.nih.gov/(Figure 2A). The web interface contains a search box where the user can input one or more official gene symbols delimited by whitespace. *Textrous!* is able to generate words and noun-phrases and their associated similarity scores, z-scores, and *p*-values. After initial Gene Symbol input and activation of ‘Submit’ the Cosine Similarity results are depicted (Figure 2B). Each output word in the ranking list can be used to link out to the top-scoring phrases associated with that specific word (Figure 2C). After initial searching, the user can interact with the results by displaying the top words Cosine Similarity as well as their Z-score and probability value tables (Figure 2D). In addition *Textrous!* allows the generation of hierarchical word clouds as well as displaying heat maps: both of these options allow the linking to noun-phrases from each word. Noun-phrases can then be traced back to their original PubMed articles. The number of genes found, as well as the genes excluded from the query, can be viewed from the search bar. All features are accessible on every page. A list of stopwords can be found at http://textrous.cit.nih.gov/genes2word/stopwords or equivalently through the Features page of the main site.

### Data Examples and Applications

*Textrous!* is able to process multiple genes with two different methodologies: collective processing and individual processing. Data tables, hierarchical clouds, and phrasing *collectively* process an entire gene set as a whole, while two-dimensional heat maps *individually* process each gene. Both methods can be advantageous for distinct reasons and often times generate idiosyncratically effective results from the same initial gene set query. Unlike the previously described gene set annotational applications, *e.g*. DAVID or WebGestalt, *Textrous!* is able to generate biomedically-relevant word association data from even just one input gene identity. Such flexibility may be advantageous when minimal numbers of important genomic and proteomic factors are extracted from experimental data.

#### Simple dataset paradigm

**Figure 3:**
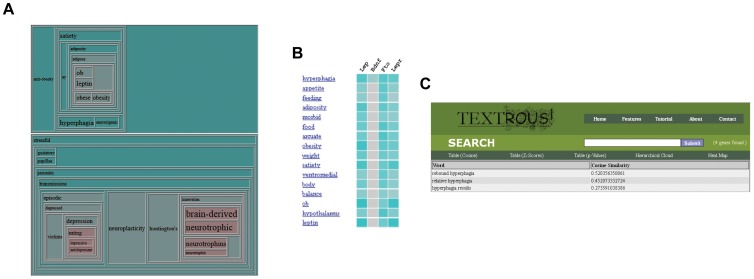
Diverse *Textrous!* processing formats. (A) An illustration of the hierarchical cloud displaying multiple themes produced by *collective* processing. The hierarchical cloud shows depression and stress at the conjunction between terms related to the central nervous system and terms related to obesity. Each cell is color-coded to represent the time at which joins were made. Font sizes are adjusted in proportion to the calculated cosine similarities. (B) An illustration of the heat map produced by *individual* processing. The top associated (Cosine Similarity) terms are shown, as well as the relationships amongst genes. Here, the heat map shows that the top words are obesity-related, and that “Bdnf” is dissimilar to the other genes in the query. Grey color indicates a relative lack of association, while the intensity of teal color corresponds directly to the strength of correlation of each pairwise association. (C) Each of the output textual terms can be hyperlinked, via clicking on the word, to their associated top-scoring (Cosine Similarity) phrases. In this panel the output word term ‘*hyperphagia*’ was linked out to its associated phrase contexts.

Using *collective* processing, the features of every gene are combined into an “average” gene, or equivalently, the sum of the vectors created by each individual gene. Such dataset management can be likened to a *gestalt* appreciation of the whole dataset ‘phenotype’. This serves as a different method of retrieving words, and leads to potentially distinct and alternatively meaningful results. Using *collective* processing in addition to hierarchical clouds allows the viewing of distinct themes within a specific gene set. One can quickly observe the presence of distinct themes in the hierarchical cloud generated by the query “*Lep Bdnf Fto Lepr*” (Lep, leptin; Bdnf, brain-derived neurotrophic factor; Fto, fat mass and obesity associated; Lepr, leptin receptor): obesity related words in the upper section, nervous system related words on the lower right, and, in the conjunction, depression related words on the lower left (Figure 3A). Additionally, *collectively-processed* phrasing allows users to clarify potentially vague terms. For example, expanding the word “gain” on the query “*Fto*” yields “body weight gain”, “excessive weight gain”, and “weight gain” as the top calculated results. Using the same method, adjectives can be traced back to the nouns that they describe. Using *individual* processing, a user is able to view the relationships between specific words and specific genes, as well as gene to gene relationships (Figure 3B). For example, a query of the metabolism- and neurotrophic-associated genes “*Lep Bdnf Fto Lepr*” shows not only which genes are responsible for which output words, with eventual associated noun-phrases, *e.g*. *hyperphagia* (Figure 3C), and at what degree. In addition from this individual processing it is evident that an additional nuance of investigation is revealed, *i.e.* Bdnf is ‘*currently*’ considered to be relatively dissimilar to the other genes in the query (Figure 3B).

To further validate the potential utility of *Textrous!* for dataset investigation we chose to next employ two considerably larger, previously validated and investigated transcriptomic datasets representing diverse molecular signatures. In these two paradigms *Textrous!* is challenged with extracting phenotypically relevant behavioral data as well as demonstrating its capacity to discriminate between two closely related molecular signaling datasets.

#### Physiological large dataset paradigm

**Figure 4:**
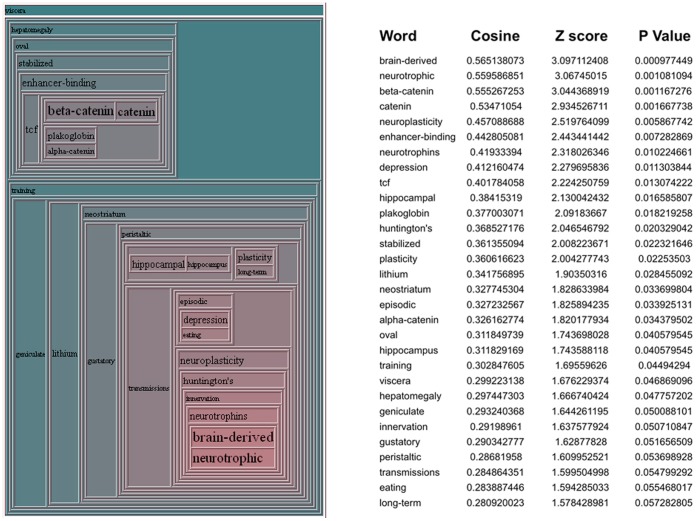
Hierarchical cloud collective processing from large physiological datasets. The hierarchical cloud represents the most strongly associated words with a large input dataset derived from behavioral experiments investigating learning task-oriented activity in mice. The highest scoring (Cosine Similarity, Z score, probability) words extracted by *Textrous!* for the input dataset are indicated next to the hierarchical cloud.

**Figure 5:**
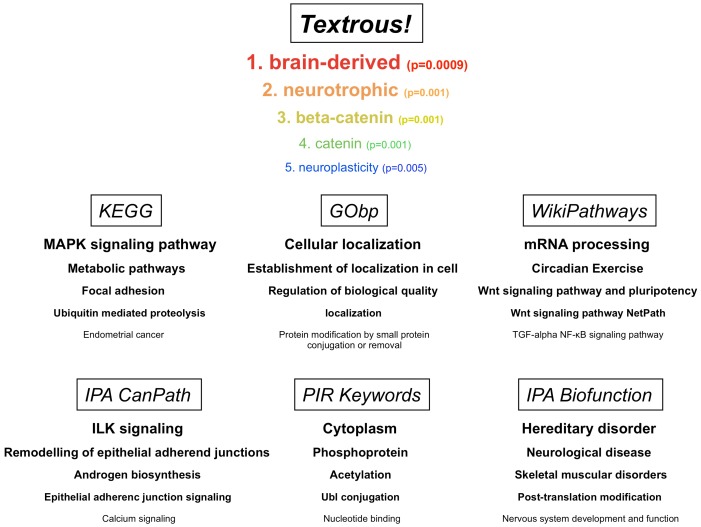
Multiple comparison of the functional accuracy and specificity of *Textrous!*-extracted data with other data analysis modules. The top five most significantly associated words obtained from *Textrous!* collective analysis of the mouse learning dataset are compared to the top 5 most significantly enriched, KEGG pathways, GO-biological processes (GO*bp*), WikiPathways, Ingenuity Pathway Analysis (IPA) Canonical Signaling Pathways (IPA CanPath), Protein Information Resource Keywords (PIR Keywords) and IPA BioFunctions generated using WebGestalt (KEGG, GObp, WikiPathways), IPA (CanPath, BioFunctions) and NIH-DAVID (PIR Keywords) respectively. The text size and descending sequential orientation indicate the first to the fifth most significantly enriched group for each analytical mode illustrated.

**Figure 6:**
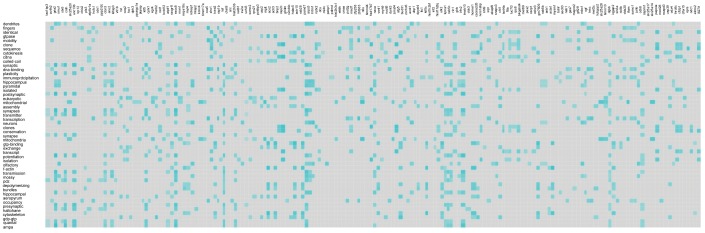
*Textrous!-*mediated individual processing output of an exemplary large dataset. The heatmap representation (teal-colored blocks indicate strongly-associated gene-word interactions in an intensity-sensitive manner: grey blocks indicate no significant interaction) indicates the gene (vertical)-word (horizontal) interactions within the large mouse learning dataset created with *Textrous!* individual processing.

To demonstrate the use of *Textrous!* for large behavioral datasets we chose a transcriptomic dataset obtained from murine central nervous tissue from experimental mice subjected to physical and cognitive tasks designed to isolate specifically the transcriptomic signatures associated with cognitive activity from transcriptional effects induced collaterally by the physical activity required to perform the cognitive task (*i.e.* Morris Water Maze) [26]. The dataset for transcripts significantly altered in the murine cortex in response to a cognitive task (Morris Water Maze completion) involving physical activity (swimming) compared to a task involving the same amount of physical activity with no goal-oriented behavior (time-controlled random swimming) is available on PubMed Central [26] and has been included in Table S1. Using this dataset (392 significantly-regulated transcripts) we derived, using *Textrous!,* the significantly associated words linked to this dataset (Table S2). In addition we also extracted the noun-phrases associated with the top 10 significantly-associated words from this list (Table S3). In Figure 4 we demonstrate the hierarchical cloud and the cosine similarities, Z scores and P values for the words forming the cloud. The strongest elements in the cloud, *e.g.* brain-derived, neurotrophic, neuroprotective and neuroplasticity are all words consistently linked with physiological activities (*e.g.* neurosynaptic reinforcement, learning, memory) as well as neurochemicals (*e.g.* brain-derived neurotrophic factor) that regulate cognitive behavior [27]–[31]. We next compared these *Textrous!*-derived hierarchical cloud outputs to a diverse array of other forms of bioinformatic analysis of the same dataset (Figure 5, Tables S2–S9). We found that with respect to the actual experimental paradigm, *i.e*. assessment of cognitive and learning behavior in mice [26], the *Textrous!* output (using the Top 5 lowest P value scoring words: numbered 1–5) was more tightly associated with the physical experiment data than the Top 5 lowest P value scoring outputs using KEGG (Table S4), GO (Table S5), WikiPathways (Table S6), IPA BioFunction (Table S7), NIH-DAVID PIR (Protein Information Resource: http://pir.georgetown.edu/) (Table S8) or IPA Canonical Pathways analysis (Table S9). In contrast to *Textrous!*, the other annotational tools (KEGG) often generate and prioritize highly generic and poorly-focused outputs, *e.g.* metabolic pathways. Therefore *Textrous!* appears to at least provide an important additional resource for extracting physiologically-relevant information from larger-scale datasets via *collective processing*. As *Textrous!* also allows simultaneous *individual processing*, via heatmap generation (Figure 6, Figure S1), we also found that the strongest gene-word associations for this specific transcript set again exhibited a profound neurophysiological learning phenotype (Figure S1, red box). For example, the strongest connections were discovered between neurophysiological words such as: dendrites; synaptic; plasticity; potentiation and transmitter, with important neurophysiological genes linked with learning such as: Gria2 (glutamate receptor, ionotropic, AMPA 2) [32]; Nrxn (Neurexin) [33]; Arc (activity-regulated cytoskeleton-associated protein) [34]; Homer1 (homer homolog 1) [35] and Rasgrf1 (Ras protein-specific guanine nucleotide-releasing factor 1) [36]. Using this physiological model example, *Textrous!* was able to generate physiologically accurate textual data extraction and presentation using *individual* as well as *collective* processing techniques from this large dataset.

#### Compare and Contrast Paradigm

**Figure 7:**
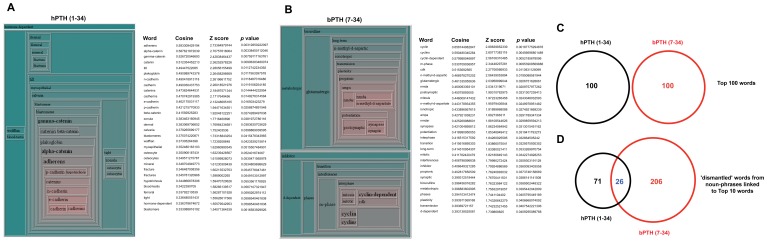
Hierarchical cloud collective processing for compare-and-contrast large datasets. (A) The hierarchical cloud represents the most strongly associated words associated with the hPTH (1–34)-induced transcriptomic response in murine calvarial bone. The highest scoring (Cosine Similarity, Z score, probability) words extracted by *Textrous!* for the input dataset are indicated next to the hierarchical cloud. (B) Hierarchical cloud representing the most strongly associated words associated with the bPTH (7–34)-induced transcriptomic response in murine calvarial bone. The highest scoring (Cosine Similarity, Z score, probability) words extracted by *Textrous!* for the input dataset are indicated next to the hierarchical cloud. (C) Venn diagram illustrating the distinct nature of collective processing-Textrous!-extracted words for the hPTH (1–34) and bPTH (7–34) datasets. (D) Venn diagram illustrating the minimal commonality between words from manually-dismantled noun-phrases from hPTH (1–34) and bPTH (7–34) datasets.

As *Textrous!* demonstrated a robust ability to extract physiologically-relevant phenotypic data from a single comparison behavioral dataset we next tested whether Textrous! would be able to facilitate discriminatory data extraction from two contrasting datasets instead. We have recently demonstrated that structurally distinct therapeutic molecules, human parathyroid hormone (hPTH (1–34)) and a molecular variant (bPTH (7–34)) can activate the same parathyroid hormone receptor in bone tissue in a G protein- or β-arrestin-dependent manner respectively [37]. The functional signaling pathways and functional sequelae entrained by these two distinct ligands both support bone development but via clearly distinguishable mechanisms. Treatment of mice with hPTH (1–34) primarily affects signaling activity associated with enhanced bone formation through collagen synthesis and matrix mineralization, while bPTH (7–34) primarily affects pathways that promote expansion of the osteoblast pool, via modulation of cell cycle regulation, cell survival, and migration. This diverse molecular activity is one of the first demonstrations of ‘biased agonist activity’ in an *in vivo* setting. As the molecular pathways of these two ligands are well characterized and mechanistically distinct we employed *Textrous!* in a compare-and-contrast manner between these two large datasets (Tables S10, S11). Using the *Textrous! collective* processing with the hierarchical clouds we found a clear distinction between the two datasets (Figure 7A–B). From the hierarchical cloud output from the hPTH (1–34) dataset that this ligand induces a classical ‘bone development’ phenotype as the most significantly associated words extracted are linked with mineralization, matrix synthesis and bone structure including: catenins [38], [39]; calvaria; osteocytes; cadherin [40] and mineral (Figure 7A, Table S12). In contrast, the words depicted in the hierarchical cloud from the bPTH (7–34) dataset include words less strongly associated with classical bone modeling activity but more with the atypical cell-cycle regulatory activity demonstrated by this ligand *in vivo* [37], *e.g*. cyclin, cyclin-dependent, cdk, m-phase and mitosis (Figure 7B, Table S13). When we compared the top 100 extracted word associations from these two datasets we found that no words were common between the two *Textrous!* extractions (Figure 7C). When the noun-phrases associated with the top 10 extracted words were compared between the hPTH (1–34) and bPTH (7–34) datasets again there were no common noun-phrases (Tables S14 and S15 respectively). Even with a manual dismantling of the individual words contained in the two noun-phrase lists only a minimal overlap between the two datasets was observed (Figure 7D). Therefore the *collective* processing module of *Textrous!* was able to both generate an accurate appreciation of the two datasets in such a manner that their distinct mechanistic natures can be confidently compared and contrasted. Indicative of the potential discovery aspect of *Textrous!* data extraction a potentially strong interaction of bPTH (7–34) with neuronal activity is also suggested by the following extracted words, *e.g.* glutamatergic, nmda and post-synaptic. Future experimentation may indeed demonstrate this potential activity of this parathyroid hormone variant.

**Figure 8:**
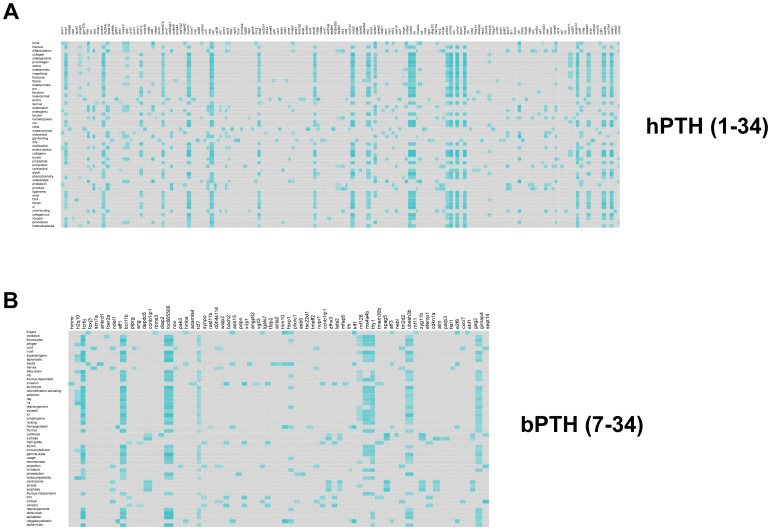
*Textrous!-*mediated individual processing output of compare-and-contrast large datasets. Individual processing heatmaps for hPTH (1–34)- and bPTH (7–34)-mediated transcriptomic activity in murine calvarial bone are demonstrated in panels (A) and (B) respectively. Teal-colored blocks indicate strongly-associated gene-word interactions in a intensity-sensitive manner, while grey blocks indicate no significant interaction.

With the *individual processing* of these contrasting datasets (Figure 8) we again found a strong distinction of *Textrous!* output. There were no common extracted heatmap words between these two datasets and the phenotypic nature of each signaling paradigm was clearly indicated. For the ‘classically-acting’ G protein-dependent hPTH (1–34) we were able to extract G protein signaling-associated words (gtp-binding, heterotrimer), bone differentiation-associated words (collagen, osteogenic, ossification, periosteum, *etc*.) and most surprisingly the word ‘pluripotent’ (Figure 8A). This final word is extremely interesting as the hPTH (1–34) ligand is considered ‘*pluripotent*’ in its signaling activity compared to the ‘β-arrestin-focused’ bPTH (7–34) [37]. In contrast to the hPTH (1–34) dataset, the individual processing (heatmap output) for the bPTH (7–34) dataset yielded extraction of words more specifically associated with alteration of cell cycle activity (s-phase, arrests, prophase, centrosomes) and cell motility (invasion, rearrangement, cytokinesis, projection). This data output therefore accurately replicates the actual *in vivo* data for the specific activity of this bPTH (7–34) receptor ligand compared to the standard hPTH (1–34) variant.

Using these three group paradigms (simple data, large physiological data and compare-and-contrast data) we have found that *Textrous!* data analysis is able to facilitate efficient and physiologically-meaningful data extraction, via multiple processing techniques, from both small and large input data lists.

## Discussion

*Textrous!* is a novel web-based bioinformatics application that extracts semantic meaning from gene sets without the use of potentially outdated curated datasets, signaling pathways, or controlled languages. It is useful in many different contexts to help biologists extract impartial and differential knowledge from large volumes of genes or proteins (using official Gene Symbol nomenclature). Using an LSI-based approach, we were able to extract both implicit and explicit links to specific input genomic factors from diverse forms of scientific literature. *Textrous!* provides the user with a wealth of functionality for visualizing words, identifying themes, interrogating results, and determining statistical significance. Taken together, the most important aspect of *Textrous!* is that it allows genomic and proteomic researchers to determine word associations to gene sets of arbitrary length in an objective, standardized, non-biased, and non-curated manner. Such a technological development therefore may possess considerable advantages over user-defined gene/protein clustering applications as it is not as reliant upon historically-derived functional signaling pathway data. It is highly likely that with further experimental evidence the rigid nature of specific gene/protein biological annotation will be demonstrated to be more and more redundant and inaccurate. Therefore in using a non-curated process, *Textrous!* provides a less biased and more ‘future-proof’ informatics data set annotation process. In conclusion, when using complicated large scale datasets *Textrous!* is able to simply create a naturalistic and unbiased scientific interpretation of these data.

## Supporting Information

Figure S1 **Interaction clustering for** ***Textrous!-*** **mediated individual processing output of an exemplary large dataset.** (A) The individual processing heatmap representation for the mouse learning dataset created with *Textrous!* was manually organized to indicate the clustering strength of the top 20 gene-word associations (indicated in red box). The top 20 most commonly associated gene-word combinations are indicated in panel (B).
(TIF)

Table S1 **Relative transcription responses for learning-based physical activity versus non-learning based physical activity.** Gene transcription z-ratios for the learning task (Morris Water Maze: *Learn*) mice compared to time-controlled non-goal oriented physical activity (*Swim*).
(DOC)

Table S2 ***Textrous!*** **output for learning task-oriented activity transcriptomic dataset.** The table indicates the Cosine similarity with the associated Z-scores and probability P values for each *Textrous!*-derived output word.
(DOC)

Table S3 ***Textrous!*** **noun-phrase output for learning task-oriented activity.** The noun-phrase output from *Textrous!* indicated below was generated from the top 10 most-significantly associated words (Table S2) from the original learning-driven transcriptomic set.
(DOC)

Table S4 **KEGG signaling pathway output for learning task-oriented activity.** KEGG signaling pathway output was prepared using WebGestalt (http://bioinfo.vanderbilt.edu/webgestalt/). The table indicates the KEGG pathway output generated using the original learning task-oriented transcriptomic dataset. The table indicates the number of reference genes in the KEGG pathway category (C), number of genes from the input set in the specific category (O), the expected number in the category (E) based on a murine background set, the ratio of enrichment (R) and p value (P: hypergeometric test, p<0.05) adjusted by multiple test adjustment.
(DOC)

Table S5 **Gene Ontology term enrichment output for learning task-oriented activity.** Gene Ontology term enrichment output was prepared using WebGestalt (http://bioinfo.vanderbilt.edu/webgestalt/). The table indicates the GO term output generated using the original learning task-oriented transcriptomic dataset. The table indicates the number of reference genes in the GO term category (C), number of genes from the input set in the specific category (O), the expected number in the category (E) based on a murine background set, the ratio of enrichment (R) and p value (P: hypergeometric test, p<0.05) adjusted by multiple test adjustment.
(DOC)

Table S6 **WikiPathways enrichment output for learning task-oriented activity.** WikiPathway term enrichment output was prepared using WebGestalt (http://bioinfo.vanderbilt.edu/webgestalt/). The table indicates the WikiPathways output generated using the original learning task-oriented transcriptomic dataset. The table indicates the number of reference genes in the specific WikiPathway category (C), number of genes from the input set in the specific category (O), the expected number in the category (E) based on a murine background set, the ratio of enrichment (R) and p value (P: hypergeometric test, p<0.05) adjusted by multiple test adjustment.
(DOC)

Table S7 **Ingenuity Pathway Analysis BioFunction enrichment output for learning task-oriented activity.** Ingenuity Pathway Analysis (IPA: http://www.ingenuity.com/products/ipa) was employed to generate specific BioFunction activity output from the murine learning transcriptomic dataset. The specific significant P value for each enriched BioFunction is indicated.
(DOC)

Table S8 **NIH DAVID PIR Keyword enrichment output for learning task-oriented activity.** The batch gene annotation module of NIH DAVID (http://david.abcc.ncifcrf.gov/) was employed for the derivation of the fold enrichment (using a murine background set) and the significant P value (<0.05) for the specifically enriched PIR keywords extracted from the learning oriented-task transcriptomic dataset.
(DOC)

Table S9 **IPA Canonical signaling pathway enrichment output for learning task-oriented activity.** Ingenuity Pathway Analysis (IPA: http://www.ingenuity.com/products/ipa) was employed to generate specific Canonical Signaling Pathway activity output from the murine learning oriented-task transcriptomic dataset. The specific significant negative log_10_ of the P value, as well as the enrichment ratio for each of the significantly-populated Canonical signaling pathways is indicated.
(DOC)

Table S10 **Parathyroid hormone (hPTH (1–34))-induced bone transcription response in wild-type mice.** The transcriptomic response data indicates the significantly regulated genes expressed in calvarial bone extracts from mice intermittently dosed with hPTH (1–34).
(DOC)

Table S11 **Parathyroid hormone variant (bPTH (7–34))-induced bone transcription responses in wild-type mice.** The transcriptomic response data indicates the significantly regulated genes expressed in calvarial bone extracts from wild-type mice intermittently dosed with the parathyroid hormone variant bPTH (7–34).
(DOC)

Table S12 ***Textrous!*** **output from hPTH (1–34) calvarial bone transcription response in wild-type mice.** The Cosine similarity, Z-scores and associated P values for the word data output (top 100) from hPTH (1–34)-treated mice is indicated in the table.
(DOC)

Table S13 ***Textrous!*** **output from bPTH (7–34) parathyroid hormone variant calvarial bone transcription responses in wild-type mice.** The Cosine similarity, Z-scores and associated P values for the word data output (top 100) from bPTH (7–34)-treated mice is indicated in the table.
(DOC)

Table S14 ***Textrous!*** **noun-phrase output from hPTH (1–34) calvarial bone transcription responses in wild-type mice.** The data indicated in the table consists of the Cosine similarity scores for the most strongly associated noun-phrases linked to the top 10 most significantly-associated words extracted by *Textrous!* from the hPTH (1–134)-induced transcriptome data.
(DOC)

Table S15 ***Textrous!*** **noun-phrase output from bPTH (7–34) parathyroid hormone variant-treatment of calvarial bone transcription responses in wild-type mice.** The data indicated in the table consists of the Cosine similarity scores for the most strongly associated noun-phrases linked to the top 10 most significantly-associated words linked to the bPTH (7–34)-induced transcriptome data.
(DOC)

## References

[1] Maudsley S, Chadwick W, Wang L, Zhou Y, Martin B, et al (2011) Bioinformatic approaches to metabolic pathways analysis. Methods Mol Biol 756: 99–130. 21870222 10.1007/978-1-61779-160-4_5 PMC4698828

[2] Jensen L, Saric J, Bork P (2006) Literature Mining for the Biologist: from Information Retrieval to Biological Discovery. Nat Rev Genet 7: 119–129. 16418747 10.1038/nrg1768

[3] Xu L, Furlotte N, Lin Y, Heinrich K, Berry MW, et al (2011) Functional Cohesion of Gene Sets Determined by Latent Semantic Indexing of PubMed Abstracts. PLoS One 6: e18851. 21533142 10.1371/journal.pone.0018851 PMC3077411

[4] Roy S, Heinrich K, Phan V, Berry MW, Homayouni R (2011) Latent Semantic Indexing of PubMed Abstracts for Identification of Transcription Factor Candidates from Microarray Derived Gene Sets. BMC Bioinformatics 12: 519–532. 10.1186/1471-2105-12-S10-S19 PMC3236841 22165960

[5] Homayouni R, Heinrich K, Wei L, Berry MW (2004) Gene Clustering by Latent Semantic Indexing of MEDLINE abstracts. Bioinformatics 21: 104–115. 15308538 10.1093/bioinformatics/bth464

[6] Coletti MH, Bleich HL (2001) Medical Subject Headings Used to Search the Biomedical Literature. J Am Med Inform Assoc 8: 317–323. 11418538 10.1136/jamia.2001.0080317 PMC130076

[7] Ashburner M, Ball CA, Blake JA, Botstein D, Butler H, et al (2000) Gene Ontology: tool for the unification of biology. The Gene Ontology Consortium. Nat Genet 25: 25–29. 10802651 10.1038/75556 PMC3037419

[8] Nishimura D (2001) A View From the Web: Biocarta. Biotech Software & Internet Report 2: 117–120.

[9] Kanehisa M (2002) The KEGG Database. Novartis Found Symp 247: 91–101. 12539951

[10] Carbon S, Ireland A, Mungall CJ, Shu SQ, Marshall B, et al (2009) AmiGO: Online Access to Ontology and Annotation Data. Bioinformatics 25: 288–289. 19033274 10.1093/bioinformatics/btn615 PMC2639003

[11] Ade AS, Wright ZC, States DJ (2007) Gene2MeSH [Internet]. Ann Arbor (MI): National Center for Integrative Biomedical Informatics. Available: http://gene2mesh.ncibi.org. Accessed 2012 Jul 1.

[12] Baroukh C, Jenkins S, Dannenfelser R, Ma’ayan A (2011) Gene2WordCloud: A Quick Way to Identify Biological Themes from Gene Lists and Free Text. Source Code Biol Med. 6: 15. 10.1186/1751-0473-6-15 PMC3213042 21995939

[13] Sarkar IN, Schenk R, Miller H, Norton CN (2009) LigerCat: using "MeSH Clouds" from journal, article, or gene citations to facilitate the identification of relevant biomedical literature. AMIA Annu Symp Proc 2009: 563–7. 20351918 PMC2815376

[14] Dennis G, Sherman BT, Hosack DA, Yang J, Gao W, et al (2003) DAVID: Database for Annotation, Visualization, and Integrated Discovery. Genome Biol 4: 3. PMC193660 12734009

[15] Becker KG, Hosack DA, Dennis G, Lempicki RA, Bright TJ (2003) PubMatrix: A Tool for Multiplex Literature Mining. BMC Bioinformatics 4: 61. 14667255 10.1186/1471-2105-4-61 PMC317283

[16] Zhang B, Kirov S, Snoddy J (2005) WebGestalt: An Integrated System for Exploring Gene Sets in Various Biological Contexts. Nucleic Acids Res 33: 741–748. 10.1093/nar/gki475 PMC1160236 15980575

[17] Subrarmanian CA, Tamayo P, Mootha VK, Mukherjee S, Ebert BL, et al (2005) Gene Set Enrichment Analysis: A Knowledge-based Approach for Interpreting Genome-wide Expression Profiles. Proc Natl Acad Sci USA 102: 15545–15550. 16199517 10.1073/pnas.0506580102 PMC1239896

[18] Deerwester S, Dumais ST, Furnas GW, Landauer TK, Harshman R (1999) Indexing by Latent Semantic Analysis. J Am Soc Inf Sci. 41: 381–407.

[19] Online Mendelian Inheritance in Man, OMIM®. McKusick-Nathans Institute of Genetic Medicine, Johns Hopkins University (Baltimore, MD). Available: http://omim.org Accessed 2012 Jul 1.

[20] SMART stoplist. Available: ftp://ftp.cs.cornell.edu/pub/smart/english.stop. Accessed 2012 Jul 6.

[21] Dumais S (2004) Latent Semantic Analysis. Annual Review of Information Science and Technology. 38: 4.

[22] Golub GH, Reinsch C (1970) Singular Value Decomposition and Least Squares Solutions. Numerische Mathematik. 14: 403–420.

[23] Jahiruddin, Abulaish M, Dey L (2010) A Concept-Driven Biomedical Knowledge Extraction of Visualization Framework for Conceptualization of Text Corpora. J Biomed Inform 43: 1020–1035. 20870033 10.1016/j.jbi.2010.09.008

[24] Berry M, Do T, O’Brien G, Krishna V, Varadhan S. SVDLIBC: A C library for Computing Singular Value Decompositions. Version 1.4.

[25] Apache OpenNLP. Available: http://opennlp.apache.org. Accessed 2012 Jul 8.

[26] Park SS, Stranahan AM, Chadwick W, Zhou Y, Wang L, et al (2011) Cortical gene transcription response patterns to water maze training in aged mice. BMC Neurosci 12: 63 doi: 10.1186/1471–2202–12–63. 21714909 10.1186/1471-2202-12-63 PMC3142531

[27] Chadwick W, Mitchell N, Caroll J, Zhou Y, Park SS, et al (2011) Amitriptyline-mediated cognitive enhancement in aged 3×Tg Alzheimer's disease mice is associated with neurogenesis and neurotrophic activity. PLoS One 6: e21660. 21738757 10.1371/journal.pone.0021660 PMC3124550

[28] Mattson MP, Maudsley S, Martin B (2004) BDNF and 5-HT: a dynamic duo in age-related neuronal plasticity and neurodegenerative disorders. Trends Neurosci 27: 589–94. 15374669 10.1016/j.tins.2004.08.001

[29] Zeng Y, Tan M, Kohyama J, Sneddon M, Watson JB, et al (2011) Epigenetic enhancement of BDNF signaling rescues synaptic plasticity in aging. J Neurosci 31: 17800–10. 22159096 10.1523/JNEUROSCI.3878-11.2011 PMC3278324

[30] Morrison JH, Baxter MG (2012) The ageing cortical synapse: hallmarks and implications for cognitive decline. Nat Rev Neurosci 13: 240–50. 22395804 10.1038/nrn3200 PMC3592200

[31] Spires-Jones T, Knafo S (2012) Spines, plasticity, and cognition in Alzheimer's model mice. Neural Plast 2012: 319836. 22203915 10.1155/2012/319836 PMC3238410

[32] Hackmann K, Matko S, Gerlach EM, von der Hagen M, Klink B, et al (2013) Partial deletion of GLRB and GRIA2 in a patient with intellectual disability. Eur J Hum Genet 21: 112–4. 22669415 10.1038/ejhg.2012.97 PMC3522202

[33] Mishina M, Uemura T, Yasumura M, Yoshida T (2012) Molecular mechanism of parallel fiber-Purkinje cell synapse formation. Front Neural Circuits 6: 90. 23189042 10.3389/fncir.2012.00090 PMC3505014

[34] Shepherd JD, Bear MF (2011) New views of Arc, a master regulator of synaptic plasticity. Nat Neurosci 14: 279–84. 21278731 10.1038/nn.2708 PMC8040377

[35] Gerstein H, O'Riordan K, Osting S, Schwarz M, Burger C (2012) Rescue of synaptic plasticity and spatial learning deficits in the hippocampus of Homer1 knockout mice by recombinant Adeno-associated viral gene delivery of Homer1c. Neurobiol Learn Mem 97: 17–29. 21945599 10.1016/j.nlm.2011.08.009 PMC3496399

[36] Fernández-Medarde A, Porteros A, de las Rivas J, Núñez A, Fuster JJ, et al (2007) Laser microdissection and microarray analysis of the hippocampus of Ras-GRF1 knockout mice reveals gene expression changes affecting signal transduction pathways related to memory and learning. Neuroscience 146: 272–85. 17321057 10.1016/j.neuroscience.2007.01.022

[37] Gesty-Palmer D, Yuan L, Martin B, Wood WH III, Lee MH, et al (2013) β-Arrestin-Selective G Protein-Coupled Receptor Agonists Engender Unique Biological Efficacy in Vivo. Mol Endocrinol 27: 296–314. 23315939 10.1210/me.2012-1091 PMC3683806

[38] Bonnet N, Conway SJ, Ferrari SL (2012) Regulation of beta catenin signaling and parathyroid hormone anabolic effects in bone by the matricellular protein periostin. Proc Natl Acad Sci U S A 109: 15048–53. 22927401 10.1073/pnas.1203085109 PMC3443161

[39] Kim TH, Bae CH, Jang EH, Yoon CY, Bae Y, et al (2012) Col1a1-cre mediated activation of β-catenin leads to aberrant dento-alveolar complex formation. Anat Cell Biol 45: 193–202. 23094208 10.5115/acb.2012.45.3.193 PMC3472146

[40] Miron RJ, Hedbom E, Ruggiero S, Bosshardt DD, Zhang Y, et al (2011) Premature osteoblast clustering by enamel matrix proteins induces osteoblast differentiation through up-regulation of connexin 43 and N-cadherin. PLoS One 6: e23375. 21858092 10.1371/journal.pone.0023375 PMC3156132

