# Supplementary figures and images for: Textrous!: Extracting Semantic Textual Meaning from Gene Sets
Source: PLoS One. 2013 Apr 30;8(4):e62665. doi: 10.1371/journal.pone.0062665 (PMC3639949; doi:10.1371/journal.pone.0062665)

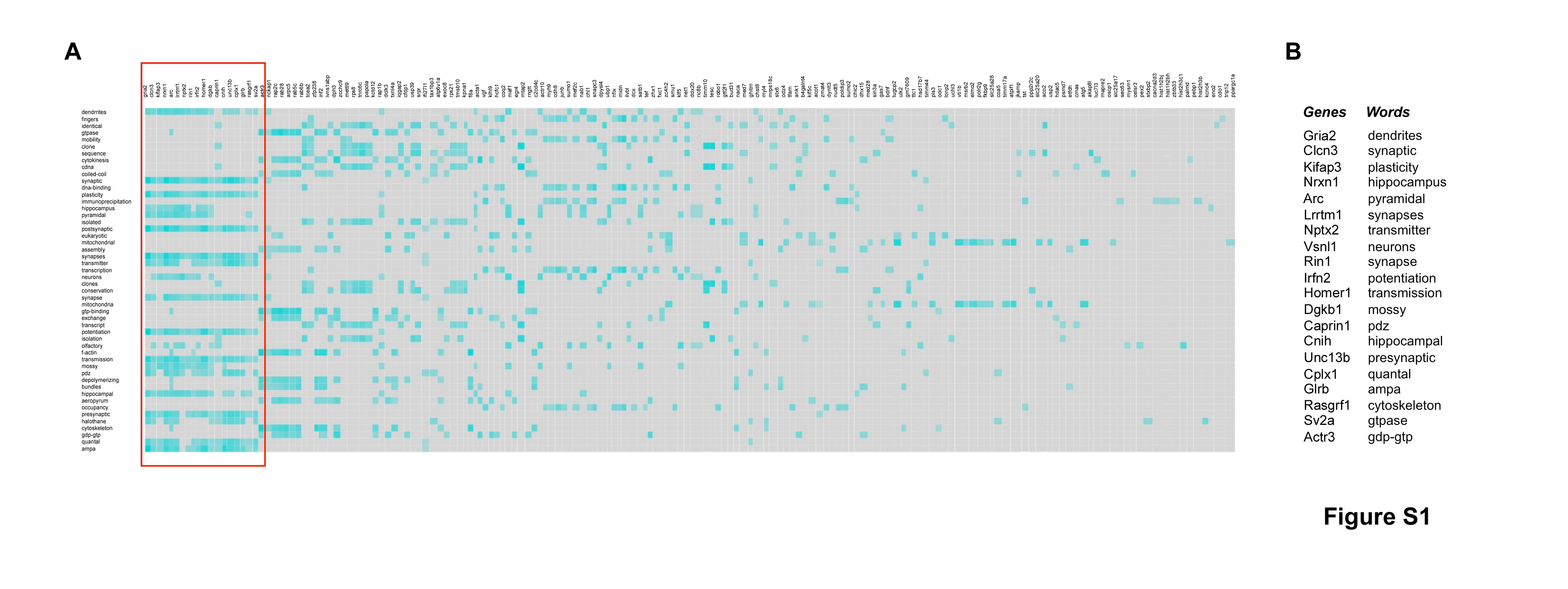

Supplement: Figure S1 — Interaction clustering for Textrous!- mediated individual processing output of an exemplary large dataset. (A) The individual processing heatmap representation for the mouse learning dataset created with Textrous! was manually organized to indicate the clustering strength of the top 20 gene-word associations (indicated in red box). The top 20 most commonly associated gene-word combinations are indicated in panel (B). (TIF) [file pone.0062665.s001.tif]
