# Supplementary material for: Textrous!: Extracting Semantic Textual Meaning from Gene Sets
Source: PLoS One. 2013 Apr 30;8(4):e62665. doi: 10.1371/journal.pone.0062665 (PMC3639949; doi:10.1371/journal.pone.0062665)
Supplement: Table S1 — Relative transcription responses for learning-based physical activity versus non-learning based physical activity. Gene transcription z-ratios for the learning task (Morris Water Maze: Learn) mice compared to time-controlled non-goal oriented physical activity (Swim). (DOC) [file pone.0062665.s002.doc]

**Table S1. Relative transcription responses for learning-based physical activity versus non-learning based physical activity.** Gene transcription z-ratios for the learning task (Morris Water Maze: *Learn*) mice compared to time-controlled non-goal oriented physical activity (*Swim*).

| **Gene Description** | **Gene Symbol** | **(zratio) Learn vs. Swim** |
| --- | --- | --- |
| activity regulated cytoskeletal-associated protein | Arc | 8.044686151 |
| dual specificity phosphatase 1 | Dusp1 | 4.687722786 |
| inhibin beta-A | Inhba | 4.658358006 |
| troponin C, cardiac/slow skeletal | Tnnc1 | 4.586315372 |
| RIKEN cDNA 6330406I15 gene | 6330406I15Rik | 4.500835076 |
| RIKEN cDNA 6330406I15 gene | 6330406I15Rik | 4.194858914 |
| early growth response 4 | Egr4 | 4.113714891 |
| tescalcin | Tesc | 3.990748043 |
| glucosaminyl (N-acetyl) transferase 2, I-branching enzyme, transcript variant 3 | Gcnt2 | 3.859773888 |
| IQ motif containing GTPase activating protein 2 | Iqgap2 | 3.858747556 |
| RIKEN cDNA D330017J20 gene | D330017J20Rik | 3.830176032 |
| RIKEN cDNA 2610204M08 gene | 2610204M08Rik | 3.729203078 |
| dual specificity phosphatase 6 | Dusp6 | 3.714384057 |
| zinc finger, DHHC domain containing 14 | Zdhhc14 | 3.519511088 |
| cyclin D1 | Ccnd1 | 3.406085095 |
| Jun-B oncogene | Junb | 3.384280668 |
| brain derived neurotrophic factor (Bdnf), transcript variant 3 | Bdnf | 3.371353018 |
| activin A receptor, type IC | Acvr1c | 3.343477507 |
| diacylglycerol kinase, beta | Dgkb | 3.249940961 |
| RIKEN cDNA 6330578E17 gene | 6330578E17Rik | 3.186544125 |
| protein phosphatase 1, catalytic subunit, beta isoform | Ppp1cb | 3.132913438 |
| F-box protein 3, transcript variant 1 | Fbxo3 | 3.100675112 |
| ubiquitin carboxyl-terminal esterase L3 | Uchl3 | 3.037555123 |
| ubiquitin carboxyl-terminal esterase L5 | Uchl5 | 3.030422602 |
| ecto-NOX disulfide-thiol exchanger 1 | Enox1 | 2.942562164 |
| schwannomin interacting protein 1 | Schip1 | 2.929331822 |
| similar to ubiquinol-cytochrome c reductase binding protein | LOC100048480 | 2.908099363 |
| RIKEN cDNA 4833439L19 gene | 4833439L19Rik | 2.895035691 |
| nudix (nucleoside diphosphate linked moiety X)-type motif 5 | Nudt5 | 2.892168202 |
| cytochrome b5 reductase 4 | Cyb5r4 | 2.84852896 |
| cell cycle associated protein 1 | Caprin1 | 2.845414841 |
| V-set and transmembrane domain containing 2B | Vstm2b | 2.834255538 |
| ADP-ribosylation factor-like 3 | Arl3 | 2.830052274 |
| cell cycle associated protein 1 | Caprin1 | 2.82061102 |
| OTU domain, ubiquitin aldehyde binding 2 | Otub2 | 2.812206151 |
| insulin receptor substrate 2 | Irs2 | 2.805178475 |
| protein phosphatase 2C, magnesium dependent, catalytic subunit | Ppm2c | 2.788403028 |
| cyclin D1 | Ccnd1 | 2.780249783 |
| myosin, light polypeptide 4 | Myl4 | 2.76265531 |
| centaurin, delta 3 | Centd3 | 2.758158336 |
| tropomyosin 4 | Tpm4 | 2.72970558 |
| vesicle transport through interaction with t-SNAREs 1B homolog | Vti1b | 2.724626992 |
| synaptotagmin XIII | Syt13 | 2.647266014 |
| influenza virus NS1A binding protein, transcript variant 2 | Ivns1abp | 2.641004992 |
| RIKEN cDNA 6530418L21 gene | 6530418L21Rik | 2.606923231 |
| histone cluster 2, H3b | Hist2h3b | 2.577215729 |
| potassium channel tetramerisation domain containing 12 | Kctd12 | 2.546845385 |
| ubiquitin-conjugating enzyme E2F (putative) | Ube2f | 2.54447325 |
| factor 8-associated gene A (F8a) | F8a | 2.538668063 |
| ubiquitin-conjugating enzyme E2G 1 (UBC7 homolog, C. elegans) | Ube2g1 | 2.53701599 |
| abl-interactor 1, transcript variant 1 | Abi1 | 2.529459797 |
| basic helix-loop-helix domain containing, class B2 | Bhlhb2 | 2.520791799 |
| VGF nerve growth factor inducible | Vgf | 2.498065597 |
| zinc finger protein 238 , transcript variant 2 | Zfp238 | 2.493675293 |
| protein phosphatase 2 (formerly 2A), regulatory subunit B (PR 52), gamma isoform | Ppp2r2c | 2.422053075 |
| SEC63-like (S. cerevisiae) | Sec63 | 2.418881472 |
| proteasome (prosome, macropain) 26S subunit, non-ATPase, 7 | Psmd7 | 2.406883554 |
| mitochondrial ribosomal protein S18C, nuclear gene encoding mitochondrial protein. | Mrps18c | 2.406398726 |
| cornichon homolog (Drosophila) | Cnih | 2.401911422 |
| small nuclear RNA activating complex, polypeptide 3 | Snapc3 | 2.401822107 |
| transcription factor A, mitochondrial | Tfam | 2.397498114 |
| RIKEN cDNA 1200015N20 gene | 1200015N20Rik | 2.38951388 |
| trophoblast glycoprotein | Tpbg | 2.381399749 |
| methyltransferase like 9 | Mettl9 | 2.374804369 |
| RAP2C, member of RAS oncogene family | Rap2c | 2.370035401 |
| trafficking protein particle complex 2 | Trappc2 | 2.367449934 |
| transmembrane protein 128 | Tmem128 | 2.361060465 |
| tribbles homolog 2 (Drosophila) | Trib2 | 2.358105911 |
| RIKEN cDNA 1700123O20 gene | 1700123O20Rik | 2.354019353 |
| growth arrest and DNA-damage-inducible 45 alpha | Gadd45a | 2.353392503 |
| RAP2C, member of RAS oncogene family | Rap2c | 2.344313714 |
| mediator complex subunit 7 | Med7 | 2.315910193 |
| acyl-CoA thioesterase 1 | Acot1 | 2.311578867 |
| RIKEN cDNA 2310008M10 gene | 2310008M10Rik | 2.293669974 |
| chloride channel 3, transcript variant a | Clcn3 | 2.277690436 |
| transmembrane protein 66 | Tmem66 | 2.265239046 |
| thyroid hormone receptor interactor 12 | Trip12 | 2.256954498 |
| glypican 1 | Gpc1 | 2.254264441 |
| doublecortin-like kinase 3 | Dclk3 | 2.242233878 |
| heat shock protein 1 (chaperonin) | Hspd1 | 2.230659203 |
| SH3-domain kinase binding protein 1 | Sh3kbp1 | 2.230318243 |
| resistance to inhibitors of cholinesterase 8 homolog B (C. elegans), transcript variant 2 | Ric8b | 2.225282305 |
| eukaryotic translation elongation factor 1 epsilon 1 | Eef1e1 | 2.22444721 |
| polymerase (RNA) II (DNA directed) polypeptide G | Polr2g | 2.212010539 |
| similar to Electron transferring flavoprotein, alpha polypeptide | LOC100046918 | 2.203738476 |
| plastin 3 (T-isoform) | Pls3 | 2.20154453 |
| kelch-like 9 (Drosophila) | Klhl9 | 2.199209389 |
| Ras and Rab interactor 1 | Rin1 | 2.194078282 |
| ankyrin repeat domain 46 | Ankrd46 | 2.190013343 |
| RNA U, small nuclear RNA export adaptor | Rnuxa | 2.189051464 |
| RIKEN cDNA 3110035E14 gene | 3110035E14Rik | 2.185769855 |
| DnaJ (Hsp40) homolog, subfamily C, member 15 | Dnajc15 | 2.182844987 |
| golgi membrane protein 1, transcript variant 1 | Golm1 | 2.177923257 |
| neuronal pentraxin 2 | Nptx2 | 2.177835415 |
| insulin-like growth factor binding protein 7 | Igfbp7 | 2.176686464 |
| transmembrane protein 33, transcript variant 2 | Tmem33 | 2.171241182 |
| ornithine decarboxylase, structural 1 | Odc1 | 2.149909697 |
| histone cluster 2, H3c1, transcript variant 1 | Hist2h3c1 | 2.136200235 |
| growth hormone inducible transmembrane protein | Ghitm | 2.1216478 |
| zinc finger matrin type 3 | Zmat3 | 2.121081389 |
| tetraspanin 14 | Tspan14 | 2.118982265 |
| cDNA sequence BC055107 | BC055107 | 2.118807418 |
| RIKEN cDNA 2610101N10 gene | 2610101N10Rik | 2.101587429 |
| SH3-binding kinase 1 | Sbk | 2.094577246 |
| SMT3 suppressor of mif two 3 homolog 1 (yeast) | Sumo1 | 2.085541094 |
| midnolin | Midn | 2.074394922 |
| cysteine-rich hydrophobic domain 2 | Chic2 | 2.065518743 |
| ATP-binding cassette, sub-family F (GCN20), member 2 | Abcf2 | 2.062902699 |
| proteasome (prosome, macropain) 26S subunit, non-ATPase, 10 | Psmd10 | 2.060120193 |
| heat shock protein 90, beta (Grp94), member 1 | Hsp90b1 | 2.059298852 |
| phenylalkylamine Ca2+ antagonist (emopamil) binding protein | Ebp | 2.05497277 |
| TYRO3 protein tyrosine kinase 3 | Tyro3 | 2.041395535 |
| protein phosphatase 2, regulatory subunit B (PR 52), beta isoform, transcript variant 2 | Ppp2r2b | 2.040468226 |
| synaptobrevin like 1 | Sybl1 | 2.036869081 |
| V-set and transmembrane domain containing 2A | Vstm2a | 2.033806594 |
| ATPase, H+ transporting, lysosomal V1 subunit A | Atp6v1a | 2.033642982 |
| progestin and adipoQ receptor family member IX | Paqr9 | 2.028096763 |
| phosphatidylethanolamine binding protein 1 | Pebp1 | 2.020868804 |
| cysteine dioxygenase 1, cytosolic | Cdo1 | 2.017631468 |
| sulfiredoxin 1 homolog (S. cerevisiae) | Srxn1 | 2.010701116 |
| karyopherin (importin) alpha 1 | Kpna1 | 2.006107337 |
| transmembrane protein 33, transcript variant 1 | Tmem33 | 1.996605408 |
| poly (A) polymerase alpha | Papola | 1.991874962 |
| nudix (nucleoside diphosphate linked moiety X)-type motif 5 | Nudt5 | 1.984160595 |
| polymerase (DNA-directed), delta interacting protein 3 | Poldip3 | 1.977908455 |
| golgi membrane protein 1, transcript variant 2 | Golm1 | 1.977688524 |
| RIKEN cDNA 1810063B05 gene | 1810063B05Rik | 1.96223384 |
| RAB5C, member RAS oncogene family | Rab5c | 1.959281591 |
| pleckstrin homology-like domain, family A, member 1 | Phlda1 | 1.946261282 |
| cytidine monophospho-N-acetylneuraminic acid synthetase | Cmas | 1.940177164 |
| transcriptional adaptor 1 (HFI1 homolog, yeast) like | Tada1l | 1.929167493 |
| 9630015D15Rik | 9630015D15Rik | 1.919807941 |
| ADP-ribosylation factor-like 8A | Arl8a | 1.919278085 |
| NCK-associated protein 1 | Nckap1 | 1.907548024 |
| thioredoxin-like 4A, transcript variant 1 | Txnl4a | 1.904226329 |
| DNA segment, Chr 15, ERATO Doi 621 | D15Ertd621e | 1.901815405 |
| ubiquitin specific peptidase 39 | Usp39 | 1.889058224 |
| roundabout homolog 1 (Drosophila) | Robo1 | 1.884890178 |
| complement component 1, q subcomponent, C chain | C1qc | 1.881206275 |
| similar to EF-hand Ca2+ binding protein p22 | LOC100048622 | 1.877076441 |
| chemokine (C-X-C motif) ligand 12 | Cxcl12 | 1.872397953 |
| histone cluster 1, H2bf | Hist1h2bf | 1.87051334 |
| ARP3 actin-related protein 3 homolog (yeast) | Actr3 | 1.866230004 |
| glycine receptor, beta subunit | Glrb | 1.865010515 |
| tetratricopeptide repeat domain 1 | Ttc1 | 1.856204166 |
| DNA segment, Chr 12, ERATO Doi 647, expressed, transcript variant 4 | D12Ertd647e | 1.848825244 |
| immediate early response 3 | Ier3 | 1.845582463 |
| ACN9 homolog (S. cerevisiae) | Acn9 | 1.841935546 |
| hydroxysteroid (17-beta) dehydrogenase 7 | Hsd17b7 | 1.831814347 |
| homer homolog 1 (Drosophila), transcript variant d | Homer1 | 1.827397708 |
| Ngfi-A binding protein 1 | Nab1 | 1.825395184 |
| 3-phosphoinositide dependent protein kinase-1, transcript variant 2 | Pdpk1 | 1.822336257 |
| engulfment and cell motility 2, ced-12 homolog (C. elegans), transcript variant 3 | Elmo2 | 1.814473054 |
| Mki67 (FHA domain) interacting nucleolar phosphoprotein | Mki67ip | 1.800989283 |
| mediator of RNA polymerase II transcription, subunit 28 homolog (yeast) | Med28 | 1.796566344 |
| zinc ribbon domain containing, 1 | Znrd1 | 1.793675726 |
| sulfiredoxin 1 homolog (S. cerevisiae) | Srxn1 | 1.789235863 |
| cAMP-regulated phosphoprotein 19 | Arpp19 | 1.780347643 |
| autophagy-related 5 (yeast) | Atg5 | 1.779530393 |
| peroxisome biogenesis factor 2 | Pex2 | 1.771612481 |
| insulin induced gene 2 | Insig2 | 1.768748426 |
| smu-1 suppressor of mec-8 and unc-52 homolog (C. elegans) | Smu1 | 1.763677445 |
| RIKEN cDNA 5730507A09 gene | 5730507A09Rik | 1.756002962 |
| inositol 1,3,4,5,6-pentakisphosphate 2-kinase | Ippk | 1.754177829 |
| actin related protein 2/3 complex, subunit 5 | Arpc5 | 1.752722767 |
| fractured callus expressed transcript 1 | Fxc1 | 1.750007009 |
| zinc finger and BTB domain containing 33, transcript variant 2 | Zbtb33 | 1.749302448 |
| ectonucleotide pyrophosphatase/phosphodiesterase 2 | Enpp2 | 1.746142915 |
| cylindromatosis (turban tumor syndrome) | Cyld | 1.740522159 |
| split hand/foot malformation (ectrodactyly) type 1 | Shfm1 | 1.740412819 |
| RIKEN cDNA 1200015F23 gene | 1200015F23Rik | 1.739284407 |
| uridine-cytidine kinase 2 | Uck2 | 1.738891517 |
| thiosulfate sulfurtransferase, mitochondrial, nuclear gene encoding mitochondrial protein | Tst | 1.736546149 |
| DEAH (Asp-Glu-Ala-His) box polypeptide 15 (Dhx15), transcript variant 2 | Dhx15 | 1.735697622 |
| mitogen-activated protein kinase kinase kinase 7 interacting protein 3 | Map3k7ip3 | 1.734609984 |
| suppressor of cytokine signaling 5 | Socs5 | 1.733921193 |
| nascent polypeptide-associated complex alpha polypeptide | Naca | 1.732015868 |
| homocysteine-inducible, endoplasmic reticulum stress-inducible, ubiquitin-like domain member 1 | Herpud1 | 1.727803349 |
| transcription elongation factor A (SII)-like 8 | Tceal8 | 1.72666566 |
| endosulfine alpha, transcript variant 2 | Ensa | 1.725656324 |
| palmdelphin | Palmd | 1.72550044 |
| solute carrier family 40 (iron-regulated transporter), member 1 | Slc40a1 | 1.724473747 |
| zinc fingers and homeoboxes 1, transcript variant 1 | Zhx1 | 1.721898271 |
| translocase of inner mitochondrial membrane 17a | Timm17a | 1.721476882 |
| steroid 5 alpha-reductase 3 | Srd5a3 | 1.718613054 |
| solute carrier family 25 (mitochondrial carrier, brain), member 14 | Slc25a14 | 1.71645956 |
| similar to CMP-sialic acid transporter | LOC100046775 | 1.712534009 |
| RIKEN cDNA A030007L17 gene | A030007L17Rik | 1.706752597 |
| thyroid hormone receptor interactor 12 | Trip12 | 1.70474628 |
| host cell factor C1 | Hcfc1 | 1.698071195 |
| Tax1 (human T-cell leukemia virus type I) binding protein 3 | Tax1bp3 | 1.697772398 |
| protein phosphatase 1, regulatory (inhibitor) subunit 3C | Ppp1r3c | 1.696513214 |
| NHL repeat containing 1 | Nhlrc1 | 1.695732311 |
| FK506 binding protein 9 | Fkbp9 | 1.69287935 |
| ubiquitin carboxyl-terminal esterase L5 | Uchl5 | 1.688975652 |
| dynein light chain Tctex-type 3 | Dynlt3 | 1.688927888 |
| sprouty protein with EVH-1 domain 1, related sequence | Spred1 | 1.685605298 |
| craniofacial development protein 1 | Cfdp1 | 1.685056021 |
| RAS related protein 1b | Rap1b | 1.679375185 |
| schwannomin interacting protein 1 | Schip1 | 1.675801181 |
| signal recognition particle receptor ('docking protein') | Srpr | 1.657469994 |
| solute carrier family 25 (mitochondrial carrier, peroxisomal membrane protein), member 17 | Slc25a17 | 1.653319799 |
| monoglyceride lipase | Mgll | 1.644878104 |
| coenzyme Q10 homolog B (S. cerevisiae), transcript variant 1 | Coq10b | 1.638752722 |
| RAB28, member RAS oncogene family | Rab28 | 1.637894637 |
| cDNA sequence BC051227 | BC051227 | 1.634844551 |
| integrin beta 5 | Itgb5 | 1.63400349 |
| BUD31 homolog (yeast) | Bud31 | 1.63139955 |
| ARP10 actin-related protein 10 homolog (S. cerevisiae) | Actr10 | 1.628714388 |
| SMT3 suppressor of mif two 3 homolog 2 (yeast) | Sumo2 | 1.62517327 |
| catenin (cadherin associated protein), beta 1 | Ctnnb1 | 1.620632854 |
| electron transferring flavoprotein, dehydrogenase | Etfdh | 1.61904407 |
| RNA guanylyltransferase and 5'-phosphatase | Rngtt | 1.618852185 |
| myosin, heavy polypeptide 9, non-muscle | Myh9 | 1.613592061 |
| leucine rich repeat and fibronectin type III domain containing 2 | Lrfn2 | 1.611548627 |
| lon peptidase 2, peroxisomal | Lonp2 | 1.609529207 |
| syntaxin 6 | Stx6 | 1.607656772 |
| transmembrane protein 198 | Tmem198 | 1.604538715 |
| GC-rich promoter binding protein 1 | Gpbp1 | 1.601062708 |
| DnaJ (Hsp40) homolog, subfamily C, member 3A | Dnajc3a | 1.596683738 |
| exocyst complex component 8 | Exoc8 | 1.596115504 |
| DPH3 homolog (KTI11, S. cerevisiae), transcript variant 1 | Dph3 | 1.593250658 |
| microfibrillar-associated protein 1B | Mfap1b | 1.593035277 |
| V-set and transmembrane domain containing 2A | Vstm2a | 1.59147535 |
| myeloid differentiation primary response gene 88 | Myd88 | 1.582469203 |
| glutamyl-prolyl-tRNA synthetase | Eprs | 1.580022624 |
| tumor necrosis factor receptor superfamily, member 19 | Tnfrsf19 | 1.578129456 |
| transmembrane protein 77, transcript variant 2 | Tmem77 | 1.568053573 |
| kinesin-associated protein 3 | Kifap3 | 1.564636021 |
| thioredoxin-like 4A | Txnl4 | 1.560454587 |
| solute carrier family 25 (mitochondrial carnitine/acylcarnitine translocase), member 20 | Slc25a20 | 1.560280217 |
| histone cluster 1, H2bj | Hist1h2bj | 1.553438141 |
| serine/threonine kinase 4 | Stk4 | 1.551207526 |
| dual specificity phosphatase 14 | Dusp14 | 1.548101429 |
| microfibrillar-associated protein 3, transcript variant 2 | Mfap3 | 1.541906422 |
| transmembrane protein 185B | Tmem185b | 1.536102181 |
| microtubule-associated protein, RP/EB family, member 2 | Mapre2 | 1.536024431 |
| TSC22 domain family 2 | Tsc22d2 | 1.535367475 |
| RUN domain containing 3B | Rundc3b | 1.532812042 |
| syndecan 2 | Sdc2 | 1.528269498 |
| nuclear import 7 homolog (S. cerevisiae) | Nip7 | 1.527620471 |
| Unc-51 like kinase 2 (C. elegans) | Ulk2 | 1.526407903 |
| protein disulfide isomerase associated 3 | Pdia3 | 1.525332795 |
| coenzyme Q2 homolog, prenyltransferase (yeast) | Coq2 | 1.524599963 |
| methionine sulfoxide reductase B2 | Msrb2 | 1.52437112 |
| solute carrier family 11 (proton-coupled divalent metal ion transporters), member 1 | Slc11a1 | 1.520292126 |
| translocase of inner mitochondrial membrane 10 homolog (yeast) | Timm10 | 1.520209446 |
| DEAD (Asp-Glu-Ala-Asp) box polypeptide 20 | Ddx20 | 1.518221347 |
| similar to FOG | LOC100047651 | 1.517077158 |
| F-box protein 33 | Fbxo33 | 1.508521001 |
| RIKEN cDNA 1200003C05 gene | 1200003C05Rik | 1.507715294 |
| histone deacetylase 5, transcript variant 1 | Hdac5 | 1.507673606 |
| cDNA sequence AK129302 | AK129302 | 1.503352943 |
| RIKEN cDNA 3830406C13 gene, transcript variant 2 | 3830406C13Rik | 1.502940947 |
| solute carrier family 35, member F3 | Slc35f3 | 1.502456328 |
| growth arrest specific 7 | Gas7 | 1.500308407 |
| gene model 347 | Gm347 | -1.509077481 |
| beta-1,4-N-acetyl-galactosaminyl transferase 4 | B4galnt4 | -1.514891214 |
| ATPase, H+ transporting, lysosomal accessory protein 1 | Atp6ap1 | -1.51689522 |
| bromodomain containing 9 | Brd9 | -1.518431559 |
| actin, alpha 1, skeletal muscle | Acta1 | -1.520329087 |
| translocase of inner mitochondrial membrane 44 | Timm44 | -1.522321053 |
| carbohydrate (N-acetylgalactosamine 4-0) sulfotransferase 8 | Chst8 | -1.525913417 |
| glutamate receptor, ionotropic, AMPA2 (alpha 2) | Gria2 | -1.535411621 |
| RIKEN cDNA 2600009E05 gene | 2600009E05Rik | -1.535452762 |
| tubulin, gamma complex associated protein 2 | Tubgcp2 | -1.539727168 |
| RIKEN cDNA B930041F14 gene | B930041F14Rik | -1.541952728 |
| yippee-like 3 (Drosophila) | Ypel3 | -1.543724205 |
| phosphatidylinositol transfer protein, alpha | Pitpna | -1.545348726 |
| solute carrier family 4 (anion exchanger), member 3 | Slc4a3 | -1.545706498 |
| zinc finger, CCHC domain containing 6 | Zcchc6 | -1.546048519 |
| stathmin-like 4 | Stmn4 | -1.550790923 |
| protein tyrosine phosphatase, receptor type, D, transcript variant a | Ptprd | -1.552235622 |
| testis expressed gene 264, transcript variant 1 | Tex264 | -1.560366159 |
| Rab6 interacting protein 1 | Rab6ip1 | -1.562323358 |
| similar to Hmgcs1 protein, transcript variant 1 | LOC100040592 | -1.564926775 |
| transcriptional regulator, SIN3A (yeast) | Sin3a | -1.571253835 |
| predicted gene, OTTMUSG00000004551 | OTTMUSG00000004551 | -1.576196902 |
| aconitase 2, mitochondrial, nuclear gene encoding mitochondrial protein | Aco2 | -1.57750234 |
| KRAB-A domain containing 1 | Krba1 | -1.592986736 |
| DEAD (Asp-Glu-Ala-Asp) box polypeptide 3, Y-linked | Ddx3y | -1.594977601 |
| myb-like, SWIRM and MPN domains 1 | Mysm1 | -1.600678845 |
| ribosomal protein S19 | Rps19 | -1.603502086 |
| transcription elongation factor A (SII), 2 | Tcea2 | -1.605539887 |
| RIKEN cDNA 1810007P19 gene | 1810007P19Rik | -1.632352395 |
| RAB6B, member RAS oncogene family | Rab6b | -1.638323061 |
| calcium channel, voltage-dependent, alpha2/delta subunit 3 | Cacna2d3 | -1.64090328 |
| kinesin family member 5C | Kif5c | -1.665679808 |
| general transcription factor IIF, polypeptide 1 | Gtf2f1 | -1.668326215 |
| enolase 2, gamma neuronal | Eno2 | -1.668881233 |
| ribosomal protein S21 | Rps21 | -1.670369973 |
| RIKEN cDNA 4932409I22 gene | 4932409I22Rik | -1.670609452 |
| expressed sequence AW549877 | AW549877 | -1.670737933 |
| insulin-like growth factor binding protein 5 | Igfbp5 | -1.676035369 |
| neuroepithelial cell transforming gene 1, transcript variant 1 | Net1 | -1.677022045 |
| axin2 | Axin2 | -1.67824863 |
| cyclic AMP-regulated phosphoprotein, 21, transcript variant 2 | Arpp21 | -1.678682724 |
| non-SMC condensin II complex, subunit D3 | Ncapd3 | -1.684914875 |
| exostoses (multiple) 1 | Ext1 | -1.698459135 |
| thyrotroph embryonic factor, transcript variant 1 | Tef | -1.700139287 |
| coiled-coil domain containing 120 | Ccdc120 | -1.704156827 |
| CTD (carboxy-terminal domain, RNA polymerase II, polypeptide A) small phosphatase-like | Ctdspl | -1.704157999 |
| MAF1 homolog (S. cerevisiae) | Maf1 | -1.717816791 |
| solute carrier family 25, member 28 | Slc25a28 | -1.734671533 |
| unconventional SNARE in the ER 1 homolog (S. cerevisiae), transcript variant 1 | Use1 | -1.75786813 |
| transketolase | Tkt | -1.758186098 |
| ATPase, Na+/K+ transporting, alpha 1 polypeptide | Atp1a1 | -1.771814587 |
| nephronophthisis 4 (juvenile) homolog (human) | Nphp4 | -1.779421341 |
| interleukin enhancer binding factor 3 | Ilf3 | -1.779998169 |
| casein kinase 1, epsilon | Csnk1e | -1.782087604 |
| cholecystokinin B receptor | Cckbr | -1.78443574 |
| RIKEN cDNA 1700019D03 gene | 1700019D03Rik | -1.788407564 |
| potassium voltage gated channel, Shaw-related subfamily, member 4 | Kcnc4 | -1.797921045 |
| microtubule associated serine/threonine kinase 1 | Mast1 | -1.799204427 |
| transmembrane protein 110 | Tmem110 | -1.801534299 |
| ribosomal protein S8 | Rps8 | -1.803539598 |
| microtubule-associated protein 2, transcript variant 2 | Mtap2 | -1.810799375 |
| gamma-aminobutyric acid (GABA(A)) receptor-associated protein-like 1 | Gabarapl1 | -1.82381847 |
| non-SMC element 2 homolog | Nsmce2 | -1.826969617 |
| TRAF type zinc finger domain containing 1 | Trafd1 | -1.8295315 |
| ubiquitin specific peptidase 52 | Usp52 | -1.837406042 |
| RNA binding motif protein 5 | Rbm5 | -1.839234431 |
| megakaryocyte-associated tyrosine kinase | Matk | -1.859159352 |
| dapper homolog 2, antagonist of beta-catenin (xenopus) | Dact2 | -1.865557381 |
| cold inducible RNA binding protein | Cirbp | -1.872057652 |
| G protein-coupled receptor associated sorting protein 1, transcript variant 3 | Gprasp1 | -1.880766873 |
| transmembrane protein 201, transcript variant 1 | Tmem201 | -1.899349228 |
| expressed sequence AI316807 | AI316807 | -1.900334963 |
| ilvB (bacterial acetolactate synthase)-like | Ilvbl | -1.916962505 |
| leukocyte tyrosine kinase, transcript variant 2 | Ltk | -1.919477251 |
| resistance to inhibitors of cholinesterase 3 homolog (C. elegans), transcript variant 1 | Ric3 | -1.921335037 |
| complexin 1 | Cplx1 | -1.947085657 |
| protein tyrosine phosphatase, receptor type, D, transcript variant a | Ptprd | -1.95047979 |
| synaptic vesicle glycoprotein 2 a | Sv2a | -1.966224119 |
| RIKEN cDNA 1110008P14 gene | 1110008P14Rik | -1.977321892 |
| upstream binding protein 1 | Ubp1 | -1.989338447 |
| peroxisome proliferative activated receptor, gamma, coactivator 1 alpha | Ppargc1a | -2.007007094 |
| eukaryotic translation elongation factor 1 alpha 2 | Eef1a2 | -2.01246694 |
| RIKEN cDNA A230050P20 gene | A230050P20Rik | -2.016575713 |
| RAS protein-specific guanine nucleotide-releasing factor 1, transcript variant 1 | Rasgrf1 | -2.023751411 |
| similar to synaptotagmin XI | LOC100045981 | -2.028464027 |
| dihydropyrimidinase-like 4 | Dpysl4 | -2.045529913 |
| Rho GTPase-activating protein | Grit | -2.049059896 |
| trinucleotide repeat containing 6a | Tnrc6a | -2.067092088 |
| src homology 2 domain-containing transforming protein D | Shd | -2.067805022 |
| synaptic vesicle glycoprotein 2 a | Sv2a | -2.069922317 |
| golgi apparatus protein 1 | Glg1 | -2.078795602 |
| ankyrin 1, erythroid | Ank1 | -2.084602139 |
| rhomboid domain containing 2 | Rhbdl7 | -2.101751766 |
| thymosin, beta 10 | Tmsb10 | -2.110415716 |
| visinin-like 1 | Vsnl1 | -2.118788381 |
| mahogunin, ring finger 1 | Mgrn1 | -2.132742484 |
| leucine rich repeat transmembrane neuronal 1 | Lrrtm1 | -2.137907098 |
| prolylcarboxypeptidase (angiotensinase C) | Prcp | -2.18104141 |
| guanylate cyclase 1, soluble, alpha 3 | Gucy1a3 | -2.198085862 |
| potassium voltage gated channel, Shaw-related subfamily, member 4 | Kcnc4 | -2.206357382 |
| nuclear receptor binding protein 2 | Nrbp2 | -2.219580838 |
| syntaxin binding protein 2 | Stxbp2 | -2.224177246 |
| cadherin 8, transcript variant 1 | Cdh8 | -2.284745953 |
| microtubule associated serine/threonine kinase 1 | Mast1 | -2.355192907 |
| cholinergic receptor, muscarinic 3, cardiac | Chrm3 | -2.396245142 |
| cell adhesion molecule with homology to L1CAM | Chl1 | -2.435009467 |
| ATPase inhibitory factor 1, nuclear gene encoding mitochondrial protein | Atpif1 | -2.450559607 |
| myocyte enhancer factor 2C | Mef2c | -2.459979113 |
| cadherin, EGF LAG seven-pass G-type receptor 3 (flamingo homolog, Drosophila) | Celsr3 | -2.475420241 |
| sestrin 1 | Sesn1 | -2.563345523 |
| neurexin I | Nrxn1 | -2.580032877 |
| RIKEN cDNA E430002G05 gene | E430002G05Rik | -2.58540067 |
| trinucleotide repeat containing 6C | Tnrc6c | -2.639630405 |
| cDNA sequence BC048546 | BC048546 | -2.740252514 |
| unc-13 homolog B (C. elegans) | Unc13b | -2.767736806 |
| RIKEN cDNA 3300001P08 gene | 3300001P08Rik | -2.783095028 |
| A kinase (PRKA) anchor protein 8-like | Akap8l | -2.78567323 |
| abhydrolase domain containing 14b | Abhd14b | -2.78614971 |
| gene model 1821, (NCBI) (Gm1821) on chromosome 14 | Gm1821 | -2.831951435 |
| ubiquitin specific peptidase 2, transcript variant 2 | Usp2 | -2.853637181 |
| amnionless | Amn | -2.896558258 |
| teashirt zinc finger family member 3 | Tshz3 | -2.917368084 |
| Kruppel-like factor 5 | Klf5 | -2.919315042 |
| cDNA sequence BC048546 | BC048546 | -2.99501593 |
| doublecortin, transcript variant 4 | Dcx | -2.99973244 |
| odd Oz/ten-m homolog 4 (Drosophila) | Odz4 | -3.029973371 |
| special AT-rich sequence binding protein 1 | Satb1 | -3.078405807 |
| RIKEN cDNA B830045N13 gene | B830045N13Rik | -3.099882888 |
| similar to Paraneoplastic antigen MA3 | LOC100046068 | -3.100578681 |
| netrin G1 | Ntng1 | -3.18019181 |
| RIKEN cDNA A930034L06 gene | A930034L06Rik | -3.444678336 |
| zinc finger, matrin type 4 | Zmat4 | -3.459829639 |
| hydroxy-delta-5-steroid dehydrogenase, 3 beta- and steroid delta-isomerase 2 | Hsd3b2 | -3.501033055 |
| predicted gene, EG665378 | EG665378 | -3.524095469 |
| nuclear factor I/X, transcript variant 2 | Nfix | -3.767612198 |
| DNA segment, human D4S114 | D0H4S114 | -4.768529588 |
