# Supplementary material for: Textrous!: Extracting Semantic Textual Meaning from Gene Sets
Source: PLoS One. 2013 Apr 30;8(4):e62665. doi: 10.1371/journal.pone.0062665 (PMC3639949; doi:10.1371/journal.pone.0062665)
Supplement: Table S2 — Textrous! output for learning task-oriented activity transcriptomic dataset. The table indicates the Cosine similarity with the associated Z-scores and probability P values for each Textrous!-derived output word. (DOC) [file pone.0062665.s003.doc]

**Table S2. *Textrous!* output for learning task-oriented activity transcriptomic dataset.** The table indicates the Cosine similarity with the associated Z-scores and probability P values for each *Textrous!*-derived output word.

| **Word** | **Cosine Similarity** | **Z score** | **P value** |
| --- | --- | --- | --- |
| brain-derived | 0.565236958 | 3.09711241 | 0.00097745 |
| neurotrophic | 0.559643106 | 3.06745015 | 0.00108109 |
| beta-catenin | 0.555286217 | 3.04436892 | 0.00116728 |
| catenin | 0.534985323 | 2.93452671 | 0.00166774 |
| neuroplasticity | 0.458402558 | 2.5197641 | 0.00586774 |
| enhancer-binding | 0.442952243 | 2.44344144 | 0.00728287 |
| neurotrophins | 0.419682032 | 2.31802635 | 0.01022466 |
| depression | 0.412737401 | 2.27969584 | 0.01130384 |
| tcf | 0.402699877 | 2.22425076 | 0.01307422 |
| hippocampal | 0.385477545 | 2.13004243 | 0.01658581 |
| plakoglobin | 0.377250178 | 2.09183667 | 0.01821926 |
| huntington's | 0.368542535 | 2.04654679 | 0.02032904 |
| plasticity | 0.361744126 | 2.00822367 | 0.02232165 |
| stabilized | 0.361197466 | 2.00427774 | 0.02253503 |
| lithium | 0.342302728 | 1.90350316 | 0.02845509 |
| neostriatum | 0.328756948 | 1.82863398 | 0.0336998 |
| episodic | 0.327862998 | 1.82589424 | 0.03392513 |
| alpha-catenin | 0.326506071 | 1.82017793 | 0.0343795 |
| hippocampus | 0.31308709 | 1.74369803 | 0.04057954 |
| oval | 0.312285576 | 1.74358812 | 0.04057954 |
| training | 0.303448648 | 1.69559626 | 0.04494294 |
| viscera | 0.299484083 | 1.67622937 | 0.0468691 |
| hepatomegaly | 0.296754369 | 1.66674042 | 0.0477572 |
| geniculate | 0.294555467 | 1.6442612 | 0.0500881 |
| innervation | 0.292215532 | 1.63757792 | 0.05071085 |
| gustatory | 0.290835133 | 1.62877828 | 0.05165651 |
| peristaltic | 0.286287686 | 1.60995252 | 0.05369893 |
| transmissions | 0.285345152 | 1.599505 | 0.05479929 |
| eating | 0.284842932 | 1.59428503 | 0.05546802 |
| long-term | 0.282267204 | 1.57842898 | 0.0572828 |
| gamma-catenin | 0.281548407 | 1.57832911 | 0.0572828 |
| cyclin | 0.279074925 | 1.56296767 | 0.0590263 |
| victims | 0.275116963 | 1.54623361 | 0.06105228 |
| stressful | 0.275060072 | 1.54553206 | 0.06105228 |
| hippocampi | 0.263920346 | 1.48581771 | 0.06863956 |
| papillae | 0.257958051 | 1.45622864 | 0.07269631 |
| synapses | 0.252144033 | 1.42102369 | 0.07765838 |
| electroconvulsive | 0.251786181 | 1.41943926 | 0.07794951 |
| synaptic | 0.25088855 | 1.41342335 | 0.07882786 |
| voluntary | 0.248320241 | 1.40071215 | 0.08060704 |
| lamb | 0.245717234 | 1.38577099 | 0.08287345 |
| stabilization | 0.245337432 | 1.38408623 | 0.08317923 |
| postsynaptic | 0.244924127 | 1.38127426 | 0.08363948 |
| cement | 0.244317342 | 1.37986718 | 0.08379332 |
| cyclins | 0.243338985 | 1.37221664 | 0.08503172 |
| mechanoreceptor | 0.241557554 | 1.3675175 | 0.08565604 |
| factor-dependent | 0.241206876 | 1.36296583 | 0.08644126 |
| trans-synaptic | 0.240944975 | 1.36078407 | 0.08675684 |
| learning | 0.240802004 | 1.36048092 | 0.08691496 |
| bipolar | 0.240214174 | 1.3555574 | 0.08754958 |
| signaling | 0.238706485 | 1.35115457 | 0.08834772 |
| depressed | 0.234876888 | 1.32737495 | 0.09225434 |
| adipogenesis | 0.232339283 | 1.31524522 | 0.09425495 |
| gyrus | 0.231311072 | 1.31437933 | 0.0944231 |
| papilla | 0.231287464 | 1.30797616 | 0.09543665 |
| gabaergic | 0.228136317 | 1.29510404 | 0.09766011 |
| experiences | 0.228073651 | 1.2911405 | 0.09835184 |
| miniature | 0.227835086 | 1.28928649 | 0.09869904 |
| pyramidal | 0.227389208 | 1.28721655 | 0.09904714 |
| cytokine | 0.226695166 | 1.28560298 | 0.09922153 |
| glutamatergic | 0.22653954 | 1.28448616 | 0.09957098 |
| pentylenetetrazol | 0.225565745 | 1.28363609 | 0.09957098 |
| saccule | 0.225061651 | 1.28227873 | 0.09992132 |
| socs | 0.223337878 | 1.27007534 | 0.10204232 |
| potentiation | 0.22154892 | 1.25222212 | 0.10528493 |
| line-derived | 0.219356801 | 1.25196809 | 0.10528493 |
| clonic | 0.218136361 | 1.24120664 | 0.10730287 |
| ampa | 0.217794209 | 1.23796326 | 0.10785803 |
| endings | 0.217701594 | 1.23512296 | 0.10841525 |
| neurotrophin | 0.215874106 | 1.22927685 | 0.1095359 |
| high-frequency | 0.214956328 | 1.22507063 | 0.11028761 |
| relapsing-remitting | 0.214578451 | 1.22259136 | 0.11066485 |
| dentate | 0.214284709 | 1.21974476 | 0.11123244 |
| spines | 0.214086672 | 1.21927943 | 0.1114221 |
| depressive | 0.213967323 | 1.21626308 | 0.11199246 |
| presynaptic | 0.21336201 | 1.21238401 | 0.11275619 |
| sedentary | 0.210959204 | 1.20158096 | 0.11468176 |
| excitatory | 0.20598492 | 1.18638529 | 0.11781115 |
| vestibular | 0.204312779 | 1.17343657 | 0.1203979 |
| kainic | 0.203626399 | 1.16347076 | 0.12241475 |
| microphthalmia-associated | 0.202911863 | 1.15903679 | 0.12322809 |
| transmission | 0.202690875 | 1.15688453 | 0.12363618 |
| quantal | 0.202164323 | 1.15405793 | 0.12425008 |
| reasoning | 0.201357956 | 1.15257768 | 0.12445519 |
| running | 0.201076138 | 1.1502742 | 0.12507194 |
| ppd | 0.197448914 | 1.13262324 | 0.12860713 |
| mtocs | 0.196868554 | 1.13114983 | 0.12902755 |
| cyclin-dependent | 0.196740566 | 1.13031887 | 0.12923811 |
| mossy | 0.196512635 | 1.12496495 | 0.13029452 |
| dephosphorylated | 0.196091343 | 1.12200659 | 0.13093122 |
| affective | 0.195146145 | 1.1160737 | 0.13221107 |
| cingulate | 0.194098053 | 1.1130215 | 0.13285421 |
| dendrite | 0.193723039 | 1.11137894 | 0.13328417 |
| memory | 0.193636191 | 1.10519367 | 0.13457979 |
| cortex | 0.19307812 | 1.10391279 | 0.13479657 |
| lingual | 0.192655979 | 1.10323581 | 0.13501358 |
| somatosensory | 0.191756026 | 1.10316274 | 0.13501358 |
| proliferation | 0.19135249 | 1.10056405 | 0.13544833 |
| convergence | 0.191294207 | 1.09991548 | 0.13566606 |
| decision | 0.191017614 | 1.09281943 | 0.1371969 |
