# Supplementary material for: Textrous!: Extracting Semantic Textual Meaning from Gene Sets
Source: PLoS One. 2013 Apr 30;8(4):e62665. doi: 10.1371/journal.pone.0062665 (PMC3639949; doi:10.1371/journal.pone.0062665)
Supplement: Table S3 — Textrous! noun-phrase output for learning task-oriented activity. The noun-phrase output from Textrous! indicated below was generated from the top 10 most-significantly associated words (Table S2) from the original learning-driven transcriptomic set. (DOC) [file pone.0062665.s004.doc]

**Table S3. *Textrous!* noun-phrase output for learning task-oriented activity.** The noun-phrase output from *Textrous!* indicated below was generated from the top 10 most-significantly associated words (Table S2) from the original learning-driven transcriptomic set.

| **Noun** | **Noun-Phrase** | **Cosine Similarity** |
| --- | --- | --- |
| brain-derived | *enhanced brain-derived neurotrophic factor* | *0.537235* |
|  | *brain-derived neurotrophic factor* | *0.535516* |
|  | *brain-derived neurotrophic factor treatment* | *0.53353* |
|  | *brain-derived neurotrophic factor promoter* | *0.523658* |
|  | *brain-derived cell lines* | *0.50721* |
|  | *brain-derived phosphatase* | *0.467784* |
|  | *brain-derived microvascular endothelial cells* | *0.274016* |
|  |  |  |
| neurotrophic | *insufficient neurotrophic support* | *0.559327* |
|  | *neurotrophic support* | *0.558475* |
|  | *neurotrophic role* | *0.557727* |
|  | *cytokine ciliary neurotrophic factor* | *0.557194* |
|  | *neurotrophic factors* | *0.557114* |
|  | *neurotrophic molecules* | *0.556503* |
|  | *neurotrophic effects* | *0.553967* |
|  | *neurotrophic agents* | *0.552855* |
|  | *neurotrophic activity* | *0.552709* |
|  | *neurotrophic cytokine leukemia inhibitory factor* | *0.542931* |
|  | *enhanced brain-derived neurotrophic factor* | *0.537235* |
|  | *brain-derived neurotrophic factor* | *0.535516* |
|  | *brain-derived neurotrophic factor treatment* | *0.53353* |
|  | *specific dopaminergic neurotrophic protein* | *0.525455* |
|  | *brain-derived neurotrophic factor promoter* | *0.523658* |
|  | *ciliary neurotrophic factor prolonged survival* | *0.512108* |
|  | *ciliary neurotrophic factor* | *0.511874* |
|  | *neurotrophic factor* | *0.511067* |
|  | *human ciliary neurotrophic factor* | *0.494307* |
|  | *brain glycoprotein neurotrophic factor* | *0.490528* |
|  | *glial cell line-derived neurotrophic factor* | *0.489498* |
|  | *conserved dopamine neurotrophic factor* | *0.339236* |
|  |  |  |
| beta-catenin | *beta-catenin signaling* | *0.569028* |
|  | *enhanced beta-catenin signaling* | *0.569017* |
|  | *beta-catenin regulation* | *0.557794* |
|  | *chick beta-catenin* | *0.557053* |
|  | *beta-catenin transactivation* | *0.556843* |
|  | *beta-catenin hyperactivity* | *0.556638* |
|  | *beta-catenin stabilization* | *0.556396* |
|  | *stabilized beta-catenin* | *0.555806* |
|  | *oncogenes beta-catenin* | *0.555352* |
|  | *beta-catenin inhibitory domain* | *0.555141* |
|  | *beta-catenin levels* | *0.555114* |
|  | *beta-catenin signal* | *0.554985* |
|  | *stabilizing beta-catenin* | *0.554783* |
|  | *excess beta-catenin* | *0.554496* |
|  | *beta-catenin turnover* | *0.55445* |
|  | *beta-catenin lead* | *0.554265* |
|  | *free beta-catenin* | *0.553996* |
|  | *beta-catenin expression* | *0.553519* |
|  | *beta-catenin heterozygosity* | *0.553435* |
|  | *beta-catenin pathways* | *0.553255* |
|  | *bind beta-catenin* | *0.553234* |
|  | *membrane-bound beta-catenin* | *0.553209* |
|  | *transient beta-catenin stabilization* | *0.552901* |
|  | *elevated beta-catenin expression* | *0.55276* |
|  | *activated beta-catenin* | *0.552553* |
|  | *increased beta-catenin* | *0.55248* |
|  | *increases beta-catenin stability* | *0.551659* |
|  | *canonical beta-catenin pathway* | *0.551514* |
|  | *beta-catenin localization* | *0.551364* |
|  | *beta-catenin destruction complex* | *0.550992* |
|  | *intracellular beta-catenin pool* | *0.550454* |
|  | *beta-catenin pathway* | *0.549958* |
|  | *beta-catenin sequences* | *0.549741* |
|  | *beta-catenin target genes* | *0.54958* |
|  | *increased beta-catenin stability* | *0.549297* |
|  | *beta-catenin signaling pathway* | *0.548619* |
|  | *beta-catenin activity* | *0.547427* |
|  | *nuclear beta-catenin* | *0.547243* |
|  | *sustained beta-catenin activity* | *0.547166* |
|  | *beta-catenin protein* | *0.545341* |
|  | *high beta-catenin activity* | *0.545111* |
|  | *higher cytosolic beta-catenin* | *0.543736* |
|  | *beta-catenin nuclear translocation* | *0.542124* |
|  | *beta-catenin promoter* | *0.541232* |
|  | *beta-catenin protein expression* | *0.540284* |
|  | *recombinant beta-catenin protein* | *0.540121* |
|  | *beta-catenin degradation* | *0.539842* |
|  | *binding beta-catenin* | *0.53906* |
|  | *beta-catenin binding* | *0.53906* |
|  | *mutant beta-catenin* | *0.538886* |
|  | *nuclear beta-catenin staining* | *0.538258* |
|  | *stabilizing beta-catenin mutant* | *0.538019* |
|  | *beta-catenin phosphorylation* | *0.537268* |
|  | *beta-catenin mutants* | *0.535801* |
|  | *nuclear beta-catenin protein* | *0.535163* |
|  | *beta-catenin regulatory domain* | *0.533428* |
|  | *beta-catenin degradation pathway* | *0.533247* |
|  | *beta-catenin interaction domain* | *0.532593* |
|  | *beta-catenin binding sites* | *0.530913* |
|  | *mutant beta-catenin activity* | *0.529088* |
|  | *aberrant beta-catenin activation* | *0.526721* |
|  | *beta-catenin signaling programs dendritic cells* | *0.526214* |
|  | *mouse beta-catenin* | *0.525744* |
|  | *beta-catenin transcription* | *0.523276* |
|  | *epithelial beta-catenin* | *0.521595* |
|  | *beta-catenin activated transcription* | *0.518452* |
|  | *beta-catenin mutation* | *0.51017* |
|  | *beta-catenin activating mutation* | *0.504402* |
|  | *activating beta-catenin mutation* | *0.504402* |
|  | *beta-catenin mutations* | *0.468744* |
|  | *beta-catenin antagonist adenomatous polyposis coli results* | *0.250323* |
|  |  |  |
| catenin | *beta catenin* | *0.331529* |
|  |  |  |
| neuroplasticity | |  |
|  |  |  |
| enhancer-binding | *enhancer-binding factors* | *0.15855* |
|  | *ccaat enhancer-binding protein-binding sites* | *0.132681* |
|  | *enhancer-binding hlh proteins* | *0.101185* |
|  | *enhancer-binding protein* | *0.068515* |
|  | *immunoglobulin enhancer-binding proteins* | *0.059298* |
|  | *polyomavirus enhancer-binding protein* | *0.056731* |
|  |  |  |
| neurotrophins | *endogenous neurotrophins* | *0.324927* |
|  |  |  |
| depression | *deep depression* | *0.408749* |
|  | *manic depression* | *0.405577* |
|  | *long-term depression* | *0.40042* |
|  | *late long-term depression* | *0.398044* |
|  | *persistent depression* | *0.393591* |
|  | *unipolar depression* | *0.391701* |
|  | *primary depression* | *0.381282* |
|  | *enhanced long-term depression* | *0.380188* |
|  | *short-term depression* | *0.376424* |
|  | *major depression* | *0.373218* |
|  | *parallel fiber long-term depression* | *0.368008* |
|  | *unipolar major depression* | *0.358455* |
|  | *spreading depression* | *0.357212* |
|  | *cerebellar long-term depression* | *0.356018* |
|  | *long-term depression production* | *0.345129* |
|  | *enhanced long-term depression induction* | *0.319305* |
|  | *synaptic depression* | *0.310197* |
|  | *cortical spreading depression* | *0.308878* |
|  | *recurrent unipolar depression* | *0.307284* |
|  | *mental depression* | *0.298672* |
|  | *increased depression* | *0.271618* |
|  | *metabotropic glutamate receptor-mediated long-term synaptic depression* | *0.240835* |
|  | *systolic contractile depression* | *0.228285* |
|  | *depression cases* | *0.220869* |
|  | *early-onset recurrent major depression* | *0.106823* |
|  | *familial unipolar depression patients* | *0.008857* |
|  |  |  |
| tcf | *tcf reporter* | *0.180122* |
|  | *tcf reporter activities* | *0.150171* |
|  | *tcf target genes* | *0.103795* |
|  | *nuclear tcf dna-binding factors* | *0.055035* |
|  | *tcf binding* | *0.029317* |
|  | *human tcf proteins* | *0.025332* |
|  |  |  |
| hippocampal | *hippocampal plasticity* | *0.394611* |
|  | *hippocampal volume* | *0.388866* |
|  | *hippocampal organizer* | *0.38685* |
|  | *hippocampal focus* | *0.386573* |
|  | *hippocampal commissure* | *0.386156* |
|  | *impaired hippocampal plasticity* | *0.38508* |
|  | *hippocampal sclerosis* | *0.383907* |
|  | *hippocampal fimbria* | *0.382764* |
|  | *hippocampal activations* | *0.38183* |
|  | *ectopic hippocampal fields* | *0.381758* |
|  | *hippocampal deficit* | *0.381748* |
|  | *mature hippocampal* | *0.380693* |
|  | *rodent hippocampal* | *0.380519* |
|  | *smaller total hippocampal volume* | *0.377803* |
|  | *lower hippocampal* | *0.377578* |
|  | *hippocampal area* | *0.375266* |
|  | *hippocampal structures* | *0.374882* |
|  | *primary hippocampal* | *0.37462* |
|  | *hippocampal areas* | *0.373567* |
|  | *hippocampal dysgenesis* | *0.372297* |
|  | *intracerebral hippocampal delivery* | *0.372101* |
|  | *hippocampal neurogenic niche* | *0.368602* |
|  | *isolated hippocampal* | *0.367353* |
|  | *hippocampal development* | *0.366598* |
|  | *hippocampal region* | *0.365738* |
|  | *hippocampal memory* | *0.365135* |
|  | *hippocampal spatial representation* | *0.364154* |
|  | *hippocampal dendrites* | *0.360568* |
|  | *adult hippocampal* | *0.359363* |
|  | *hippocampal formation* | *0.357635* |
|  | *hippocampal synapses* | *0.353844* |
|  | *hippocampal field potentials* | *0.351755* |
|  | *single hippocampal neuron* | *0.351566* |
|  | *hippocampal seizures* | *0.350125* |
|  | *hippocampal dentate gyrus* | *0.34959* |
|  | *hippocampal late long-term potentiation* | *0.348453* |
|  | *hippocampal long-term potentiation* | *0.348285* |
|  | *hippocampal complex* | *0.347487* |
|  | *enhanced hippocampal long-term potentiation* | *0.347187* |
|  | *hippocampal memory system* | *0.344479* |
|  | *hippocampal neuron development* | *0.342896* |
|  | *hippocampal atrophy* | *0.340404* |
|  | *developing hippocampal dentate gyrus* | *0.339545* |
|  | *hippocampal levels* | *0.338784* |
|  | *hippocampal mossy fiber long-term potentiation* | *0.335962* |
|  | *reduced hippocampal neurogenesis* | *0.332409* |
|  | *hippocampal synaptic plasticity* | *0.3316* |
|  | *hippocampal neural progenitor proliferation* | *0.323582* |
|  | *adult hippocampal neurogenesis* | *0.32019* |
|  | *hippocampal tissue* | *0.319319* |
|  | *hippocampal mossy fibers* | *0.319058* |
|  | *hippocampal interneurons* | *0.318636* |
|  | *hippocampal long-term synaptic potentiation* | *0.315611* |
|  | *postmortem hippocampal tissue* | *0.314437* |
|  | *hippocampal synaptic transmission* | *0.313446* |
|  | *hippocampal expression* | *0.311303* |
|  | *hippocampal dysfunction* | *0.309073* |
|  | *hippocampal mossy fiber axons* | *0.286754* |
|  | *defective postnatal hippocampal development* | *0.286573* |
|  | *human hippocampal library* | *0.281527* |
|  | *hippocampal dendritic atrophy* | *0.280196* |
|  | *hippocampal pyramidal cell layers* | *0.27266* |
|  | *hippocampal pyramidal cell layer* | *0.270332* |
|  | *human hippocampal formation* | *0.266475* |
|  | *hippocampal neuronal processes* | *0.261833* |
|  | *glutamatergic hippocampal neurons* | *0.255886* |
|  | *isolated hippocampal neuronal cultures* | *0.254694* |
|  | *hippocampal protein synthesis* | *0.253405* |
|  | *hippocampal neuronal cholinergic stimulation* | *0.253334* |
|  | *hippocampal cell lines* | *0.24809* |
|  | *hippocampal pyramidal neurons* | *0.241575* |
|  | *rodent hippocampal neurons* | *0.240711* |
|  | *mature hippocampal neurons* | *0.239972* |
|  | *hippocampal neurons* | *0.239362* |
|  | *primary hippocampal neurons* | *0.238759* |
|  | *hippocampal primary neurons* | *0.238759* |
|  | *hippocampal cell death* | *0.238254* |
|  | *single hippocampal neurons* | *0.237876* |
|  | *dissociated hippocampal neurons* | *0.237037* |
|  | *early hippocampal neurons* | *0.236454* |
|  | *rat hippocampal synaptic vesicles* | *0.235334* |
|  | *adult hippocampal neurons* | *0.233472* |
|  | *hippocampal granule neurons* | *0.232057* |
|  | *hippocampal basal protein synthesis* | *0.230772* |
|  | *cultured hippocampal neurons* | *0.228735* |
|  | *cultured rodent primary hippocampal neurons* | *0.228507* |
|  | *embryonic hippocampal neurons* | *0.220137* |
|  | *hippocampal damage* | *0.219967* |
|  | *hippocampal neuronal growth cones* | *0.212005* |
|  | *hippocampal ampa receptors* | *0.205601* |
|  | *abnormal hippocampal activation* | *0.201265* |
|  | *mutant hippocampal neurons* | *0.199304* |
|  | *apoptotic hippocampal neurons* | *0.196951* |
|  | *hippocampal nmda receptors* | *0.194618* |
|  | *hippocampal cdna* | *0.192009* |
|  | *mouse hippocampal* | *0.184867* |
|  | *hippocampal pyramidal cells* | *0.181406* |
|  | *rat hippocampal neuron dendrites* | *0.179442* |
|  | *individual hippocampal pyramidal cells* | *0.178673* |
|  | *mouse hippocampal neurons* | *0.17652* |
