# Supplementary material for: Textrous!: Extracting Semantic Textual Meaning from Gene Sets
Source: PLoS One. 2013 Apr 30;8(4):e62665. doi: 10.1371/journal.pone.0062665 (PMC3639949; doi:10.1371/journal.pone.0062665)
Supplement: Table S4 — KEGG signaling pathway output for learning task-oriented activity. KEGG signaling pathway output was prepared using WebGestalt (http://bioinfo.vanderbilt.edu/webgestalt/). The table indicates the KEGG pathway output generated using the original learning task-oriented transcriptomic dataset. The table indicates the number of reference genes in the KEGG pathway category (C), number of genes from the input set in the specific category (O), the expected number in the category (E) based on a murine background set, the ratio of enrichment (R) and p value (P: hypergeometric test, p<0.05) adjusted by multiple test adjustment. (DOC) [file pone.0062665.s005.doc]

**Table S4. KEGG signaling pathway output for learning task-oriented activity.** KEGG signaling pathway output was prepared using WebGestalt (http://bioinfo.vanderbilt.edu/webgestalt/). The table indicates the KEGG pathway output generated using the original learning task-oriented transcriptomic dataset. The table indicates the number of reference genes in the KEGG pathway category (C), number of genes from the input set in the specific category (O), the expected number in the category (E) based on a murine background set, the ratio of enrichment (R) and p value (P: hypergeometric test, p<0.05) adjusted by multiple test adjustment.

| **KEGG Pathway** | **C** | **E** | **O** | **R** | **P** |
| --- | --- | --- | --- | --- | --- |
| MAPK signaling pathway | 281 | 12 | 1.55 | 7.72 | 4.54E-06 |
| Metabolic pathways | 1229 | 21 | 6.79 | 3.09 | 0.0002 |
| Focal adhesion | 207 | 7 | 1.14 | 6.12 | 0.0032 |
| Ubiquitin mediated proteolysis | 147 | 6 | 0.81 | 7.38 | 0.0032 |
| Endometrial cancer | 56 | 4 | 0.31 | 12.92 | 0.0038 |
| Insulin signaling pathway | 146 | 5 | 0.81 | 6.19 | 0.0075 |
| SNARE interactions in vesicular transport | 38 | 3 | 0.21 | 14.28 | 0.0075 |
| Colorectal cancer | 94 | 4 | 0.52 | 7.7 | 0.0075 |
| Regulation of autophagy | 36 | 3 | 0.2 | 15.07 | 0.0075 |
| Wnt signaling pathway | 160 | 5 | 0.88 | 5.65 | 0.0075 |
| Prostate cancer | 94 | 4 | 0.52 | 7.7 | 0.0075 |
| p53 signaling pathway | 76 | 4 | 0.42 | 9.52 | 0.0075 |
| Regulation of actin cytoskeleton | 227 | 6 | 1.25 | 4.78 | 0.0075 |
| Dilated cardiomyopathy | 94 | 4 | 0.52 | 7.7 | 0.0075 |
| Hypertrophic cardiomyopathy (HCM) | 85 | 4 | 0.47 | 8.51 | 0.0075 |
| Cardiac muscle contraction | 88 | 4 | 0.49 | 8.22 | 0.0075 |
| Long-term potentiation | 81 | 4 | 0.45 | 8.93 | 0.0075 |
| Systemic lupus erythematosus | 179 | 5 | 0.99 | 5.05 | 0.0117 |
| Steroid biosynthesis | 17 | 2 | 0.09 | 21.28 | 0.0131 |
| Non-small cell lung cancer | 58 | 3 | 0.32 | 9.36 | 0.0131 |
| RNA degradation | 69 | 3 | 0.38 | 7.86 | 0.0204 |
| RIG-I-like receptor signaling pathway | 70 | 3 | 0.39 | 7.75 | 0.0204 |
| Axon guidance | 139 | 4 | 0.77 | 5.21 | 0.0211 |
| Tight junction | 144 | 4 | 0.8 | 5.02 | 0.0223 |
| Arrhythmogenic right ventricular cardiomyopathy (ARVC) | 76 | 3 | 0.42 | 7.14 | 0.0223 |
| Huntington's disease | 231 | 5 | 1.28 | 3.92 | 0.0234 |
| ECM-receptor interaction | 84 | 3 | 0.46 | 6.46 | 0.0273 |
| RNA polymerase | 32 | 2 | 0.18 | 11.31 | 0.0279 |
| Thyroid cancer | 31 | 2 | 0.17 | 11.67 | 0.0279 |
| Purine metabolism | 163 | 4 | 0.9 | 4.44 | 0.0279 |
| Pathways in cancer | 344 | 6 | 1.9 | 3.16 | 0.0279 |
| Pyrimidine metabolism | 102 | 3 | 0.56 | 5.32 | 0.0384 |
| Androgen and estrogen metabolism | 41 | 2 | 0.23 | 8.82 | 0.0421 |
