# Supplementary material for: Textrous!: Extracting Semantic Textual Meaning from Gene Sets
Source: PLoS One. 2013 Apr 30;8(4):e62665. doi: 10.1371/journal.pone.0062665 (PMC3639949; doi:10.1371/journal.pone.0062665)
Supplement: Table S5 — Gene Ontology term enrichment output for learning task-oriented activity. Gene Ontology term enrichment output was prepared using WebGestalt (http://bioinfo.vanderbilt.edu/webgestalt/). The table indicates the GO term output generated using the original learning task-oriented transcriptomic dataset. The table indicates the number of reference genes in the GO term category (C), number of genes from the input set in the specific category (O), the expected number in the category (E) based on a murine background set, the ratio of enrichment (R) and p value (P: hypergeometric test, p<0.05) adjusted by multiple test adjustment. (DOC) [file pone.0062665.s006.doc]

**Table S5. Gene Ontology term enrichment output for learning task-oriented activity.** Gene Ontology term enrichment output was prepared using WebGestalt (http://bioinfo.vanderbilt.edu/webgestalt/). The table indicates the GO term output generated using the original learning task-oriented transcriptomic dataset. The table indicates the number of reference genes in the GO term category (C), number of genes from the input set in the specific category (O), the expected number in the category (E) based on a murine background set, the ratio of enrichment (R) and p value (P: hypergeometric test, p<0.05) adjusted by multiple test adjustment.

| **GO Term ID** | **GO Term Description** | **C** | **O** | **E** | **R** | **P** |
| --- | --- | --- | --- | --- | --- | --- |
|  | **Biological Process** |  |  |  |  |  |
| GO:0051641 | cellular localization | 644 | 29 | 11.3 | 2.57 | 0.0023 |
| GO:0051649 | establishment of localization in cell | 603 | 26 | 10.58 | 2.46 | 0.0066 |
| GO:0065008 | regulation of biological quality | 1035 | 37 | 18.16 | 2.04 | 0.0066 |
| GO:0051179 | localization | 2566 | 69 | 45.01 | 1.53 | 0.0152 |
| GO:0070647 | protein modification by small protein conjugation or removal | 81 | 8 | 1.42 | 5.63 | 0.0152 |
| GO:0032446 | protein modification by small protein conjugation | 69 | 7 | 1.21 | 5.78 | 0.0253 |
| GO:0030030 | cell projection organization | 329 | 15 | 5.77 | 2.6 | 0.036 |
| GO:0016043 | cellular component organization | 1799 | 50 | 31.56 | 1.58 | 0.036 |
| GO:0044267 | cellular protein metabolic process | 2020 | 55 | 35.44 | 1.55 | 0.036 |
| GO:0051234 | establishment of localization | 2240 | 59 | 39.3 | 1.5 | 0.036 |
| GO:0003001 | generation of a signal involved in cell-cell signaling | 115 | 8 | 2.02 | 3.97 | 0.036 |
| GO:0010894 | negative regulation of steroid biosynthetic process | 3 | 2 | 0.05 | 38 | 0.036 |
| GO:0045939 | negative regulation of steroid metabolic process | 3 | 2 | 0.05 | 38 | 0.036 |
| GO:0031175 | neuron projection development | 228 | 12 | 4 | 3 | 0.036 |
| GO:0090066 | regulation of anatomical structure size | 193 | 11 | 3.39 | 3.25 | 0.036 |
| GO:0032940 | secretion by cell | 235 | 12 | 4.12 | 2.91 | 0.036 |
| GO:0046903 | secretion | 263 | 13 | 4.61 | 2.82 | 0.036 |
| GO:0006810 | transport | 2227 | 59 | 39.07 | 1.51 | 0.036 |
| GO:0016192 | vesicle-mediated transport | 435 | 18 | 7.63 | 2.36 | 0.036 |
| GO:0009987 | cellular process | 11150 | 215 | 195.6 | 1.1 | 0.0361 |
| GO:0030073 | insulin secretion | 44 | 5 | 0.77 | 6.48 | 0.0361 |
| GO:0001649 | osteoblast differentiation | 46 | 5 | 0.81 | 6.2 | 0.0414 |
| GO:0000902 | cell morphogenesis | 355 | 15 | 6.23 | 2.41 | 0.0434 |
| GO:0007492 | endoderm development | 29 | 4 | 0.51 | 7.86 | 0.0434 |
| GO:0043632 | modification-dependent macromolecule catabolic process | 468 | 18 | 8.21 | 2.19 | 0.0434 |
| GO:0019941 | modification-dependent protein catabolic process | 468 | 18 | 8.21 | 2.19 | 0.0434 |
| GO:0043687 | post-translational protein modification | 1000 | 31 | 17.54 | 1.77 | 0.0434 |
| GO:0032535 | regulation of cellular component size | 153 | 9 | 2.68 | 3.35 | 0.0434 |
| GO:0007267 | cell-cell signaling | 324 | 14 | 5.68 | 2.46 | 0.0455 |
| GO:0051055 | negative regulation of lipid biosynthetic process | 4 | 2 | 0.07 | 28.5 | 0.0455 |
| GO:0030072 | peptide hormone secretion | 51 | 5 | 0.89 | 5.59 | 0.0465 |
| GO:0000904 | cell morphogenesis involved in differentiation | 226 | 11 | 3.96 | 2.77 | 0.0485 |
| GO:0034621 | cellular macromolecular complex subunit organization | 262 | 12 | 4.6 | 2.61 | 0.0485 |
| GO:0048812 | neuron projection morphogenesis | 194 | 10 | 3.4 | 2.94 | 0.0485 |
| GO:0002790 | peptide secretion | 53 | 5 | 0.93 | 5.38 | 0.0485 |
| GO:0051603 | proteolysis involved in cellular protein catabolic process | 483 | 18 | 8.47 | 2.12 | 0.0485 |
| GO:0034622 | cellular macromolecular complex assembly | 230 | 11 | 4.03 | 2.73 | 0.0487 |
| GO:0044257 | cellular protein catabolic process | 486 | 18 | 8.53 | 2.11 | 0.0487 |
| GO:0007268 | synaptic transmission | 197 | 10 | 3.46 | 2.89 | 0.0487 |
|  | **Cellular Compartment** |  |  |  |  |  |
| GO:0044424 | intracellular part | 9057 | 221 | 164.05 | 1.35 | 4.72E-11 |
| GO:0005622 | intracellular | 9293 | 225 | 168.33 | 1.34 | 4.72E-11 |
| GO:0005737 | cytoplasm | 6135 | 163 | 111.13 | 1.47 | 1.33E-08 |
| GO:0043229 | intracellular organelle | 7707 | 180 | 139.6 | 1.29 | 2.47E-05 |
| GO:0043226 | organelle | 7711 | 180 | 139.67 | 1.29 | 2.47E-05 |
| GO:0044444 | cytoplasmic part | 3650 | 98 | 66.11 | 1.48 | 0.0003 |
| GO:0043231 | intracellular membrane-bounded organelle | 6827 | 156 | 123.66 | 1.26 | 0.0013 |
| GO:0043227 | membrane-bounded organelle | 6833 | 156 | 123.77 | 1.26 | 0.0013 |
| GO:0044464 | cell part | 13804 | 269 | 250.04 | 1.08 | 0.0014 |
| GO:0005623 | cell | 13805 | 269 | 250.06 | 1.08 | 0.0014 |
| GO:0031252 | cell leading edge | 86 | 8 | 1.56 | 5.14 | 0.0027 |
| GO:0030027 | lamellipodium | 50 | 6 | 0.91 | 6.62 | 0.0038 |
| GO:0032991 | macromolecular complex | 2161 | 59 | 39.14 | 1.51 | 0.0081 |
| GO:0043234 | protein complex | 1636 | 47 | 29.63 | 1.59 | 0.0091 |
| GO:0045202 | synapse | 292 | 14 | 5.29 | 2.65 | 0.0091 |
| GO:0042995 | cell projection | 491 | 19 | 8.89 | 2.14 | 0.0151 |
| GO:0005758 | mitochondrial intermembrane space | 16 | 3 | 0.29 | 10.35 | 0.0249 |
| GO:0005744 | mitochondrial inner membrane presequence translocase complex | 5 | 2 | 0.09 | 22.08 | 0.0268 |
| GO:0005875 | microtubule associated complex | 58 | 5 | 1.05 | 4.76 | 0.031 |
| GO:0042719 | mitochondrial intermembrane space protein transporter complex | 6 | 2 | 0.11 | 18.4 | 0.0355 |
| GO:0043232 | intracellular non-membrane-bounded organelle | 1610 | 43 | 29.16 | 1.47 | 0.0419 |
| GO:0043228 | non-membrane-bounded organelle | 1610 | 43 | 29.16 | 1.47 | 0.0419 |
| GO:0033180 | proton-transporting V-type ATPase, V1 domain | 7 | 2 | 0.13 | 15.77 | 0.0427 |
| GO:0044456 | synapse part | 186 | 9 | 3.37 | 2.67 | 0.0434 |
| GO:0005776 | autophagic vacuole | 8 | 2 | 0.14 | 13.8 | 0.0494 |
| GO:0005856 | cytoskeleton | 999 | 29 | 18.1 | 1.6 | 0.0494 |
|  | **Molecular Function** |  |  |  |  |  |
| GO:0005488 | binding | 10190 | 211 | 177.6 | 1.19 | 0.0005 |
| GO:0000166 | nucleotide binding | 1955 | 56 | 34.07 | 1.64 | 0.0054 |
| GO:0005515 | protein binding | 5149 | 120 | 89.74 | 1.34 | 0.0054 |
| GO:0004221 | ubiquitin thiolesterase activity | 61 | 7 | 1.06 | 6.58 | 0.0054 |
| GO:0016790 | thiolester hydrolase activity | 89 | 8 | 1.55 | 5.16 | 0.0086 |
| GO:0032555 | purine ribonucleotide binding | 1646 | 47 | 28.69 | 1.64 | 0.0123 |
| GO:0032553 | ribonucleotide binding | 1646 | 47 | 28.69 | 1.64 | 0.0123 |
| GO:0033549 | MAP kinase phosphatase activity | 11 | 3 | 0.19 | 15.65 | 0.0191 |
| GO:0017017 | MAP kinase tyrosine/serine/threonine phosphatase activity | 11 | 3 | 0.19 | 15.65 | 0.0191 |
| GO:0017076 | purine nucleotide binding | 1710 | 47 | 29.8 | 1.58 | 0.0215 |
| GO:0008092 | cytoskeletal protein binding | 354 | 15 | 6.17 | 2.43 | 0.0274 |
| GO:0016779 | nucleotidyltransferase activity | 106 | 7 | 1.85 | 3.79 | 0.0413 |
| GO:0019787 | small conjugating protein ligase activity | 106 | 7 | 1.85 | 3.79 | 0.0413 |
| GO:0008138 | protein tyrosine/serine/threonine phosphatase activity | 35 | 4 | 0.61 | 6.56 | 0.0476 |
