# Supplementary material for: Textrous!: Extracting Semantic Textual Meaning from Gene Sets
Source: PLoS One. 2013 Apr 30;8(4):e62665. doi: 10.1371/journal.pone.0062665 (PMC3639949; doi:10.1371/journal.pone.0062665)
Supplement: Table S6 — WikiPathways enrichment output for learning task-oriented activity. WikiPathway term enrichment output was prepared using WebGestalt (http://bioinfo.vanderbilt.edu/webgestalt/). The table indicates the WikiPathways output generated using the original learning task-oriented transcriptomic dataset. The table indicates the number of reference genes in the specific WikiPathway category (C), number of genes from the input set in the specific category (O), the expected number in the category (E) based on a murine background set, the ratio of enrichment (R) and p value (P: hypergeometric test, p<0.05) adjusted by multiple test adjustment. (DOC) [file pone.0062665.s007.doc]

**Table S6. WikiPathways enrichment output for learning task-oriented activity.** WikiPathway term enrichment output was prepared using WebGestalt (http://bioinfo.vanderbilt.edu/webgestalt/). The table indicates the WikiPathways output generated using the original learning task-oriented transcriptomic dataset. The table indicates the number of reference genes in the specific WikiPathway category (C), number of genes from the input set in the specific category (O), the expected number in the category (E) based on a murine background set, the ratio of enrichment (R) and p value (P: hypergeometric test, p<0.05) adjusted by multiple test adjustment.

| **WikiPathways** | **C** | **O** | **E** | **R** | **P** |
| --- | --- | --- | --- | --- | --- |
| mRNA processing | 536 | 17 | 2.96 | 5.74 | 5.18E-07 |
| Circadian Exercise | 65 | 6 | 0.36 | 16.7 | 2.99E-05 |
| Wnt Signaling Pathway and Pluripotency | 104 | 7 | 0.57 | 12.18 | 2.99E-05 |
| Wnt Signaling Pathway NetPath | 135 | 7 | 0.75 | 9.38 | 0.0001 |
| TNF-alpha NF-kB Signaling Pathway | 203 | 8 | 1.12 | 7.13 | 0.0002 |
| TGF-beta Receptor Signaling Pathway | 164 | 7 | 0.91 | 7.72 | 0.0003 |
| Striated Muscle Contraction | 44 | 4 | 0.24 | 16.44 | 0.0005 |
| Adipogenesis | 139 | 6 | 0.77 | 7.81 | 0.0005 |
| MAPK signaling pathway | 181 | 7 | 1 | 7 | 0.0005 |
| Mitochondrial Gene Expression | 20 | 3 | 0.11 | 27.13 | 0.0009 |
| Diurnally regulated genes with circadian orthologs | 58 | 4 | 0.32 | 12.48 | 0.0012 |
| B Cell Receptor Signaling Pathway | 170 | 6 | 0.94 | 6.38 | 0.0015 |
| Kit Receptor Signaling Pathway | 70 | 4 | 0.39 | 10.34 | 0.0021 |
| estrogen signalling | 84 | 4 | 0.46 | 8.61 | 0.0042 |
| Steroid Biosynthesis | 15 | 2 | 0.08 | 24.12 | 0.0093 |
| EGFR1 Signaling Pathway | 188 | 5 | 1.04 | 4.81 | 0.0115 |
| Senescence and Autophagy | 60 | 3 | 0.33 | 9.04 | 0.0119 |
| Leptin Insulin Overlap | 19 | 2 | 0.11 | 19.04 | 0.0123 |
| Wnt Signaling Pathway | 63 | 3 | 0.35 | 8.61 | 0.0123 |
| Proteasome Degradation | 76 | 3 | 0.42 | 7.14 | 0.0196 |
| Androgen Receptor Signaling Pathway | 150 | 4 | 0.83 | 4.82 | 0.0212 |
| Delta-Notch Signaling Pathway | 88 | 3 | 0.49 | 6.17 | 0.0266 |
| Toll Like Receptor signaling | 33 | 2 | 0.18 | 10.96 | 0.0282 |
| Glycogen Metabolism | 36 | 2 | 0.2 | 10.05 | 0.0298 |
| Hypothetical Network for Drug Addiction | 35 | 2 | 0.19 | 10.34 | 0.0298 |
| Myometrial Relaxation and Contraction Pathways | 177 | 4 | 0.98 | 4.09 | 0.0298 |
| Amino Acid metabolism - Sokolovic | 102 | 3 | 0.56 | 5.32 | 0.032 |
| Integrin-mediated cell adhesion | 105 | 3 | 0.58 | 5.17 | 0.0333 |
| G13 Signaling Pathway | 44 | 2 | 0.24 | 8.22 | 0.0364 |
| Oxidative Stress | 44 | 2 | 0.24 | 8.22 | 0.0364 |
| Focal Adhesion | 199 | 4 | 1.1 | 3.64 | 0.0364 |
