# Supplementary material for: Textrous!: Extracting Semantic Textual Meaning from Gene Sets
Source: PLoS One. 2013 Apr 30;8(4):e62665. doi: 10.1371/journal.pone.0062665 (PMC3639949; doi:10.1371/journal.pone.0062665)
Supplement: Table S7 — Ingenuity Pathway Analysis BioFunction enrichment output for learning task-oriented activity. Ingenuity Pathway Analysis (IPA: http://www.ingenuity.com/products/ipa) was employed to generate specific BioFunction activity output from the murine learning transcriptomic dataset. The specific significant P value for each enriched BioFunction is indicated. (DOC) [file pone.0062665.s008.doc]

**Table S7. Ingenuity Pathway Analysis BioFunction enrichment output for learning task-oriented activity.** Ingenuity Pathway Analysis (IPA: http://www.ingenuity.com/products/ipa) was employed to generate specific BioFunction activity output from the murine learning transcriptomic dataset. The specific significant P value for each enriched BioFunction is indicated.

| **BioFunction** | **P value** |
| --- | --- |
| Hereditary Disorder | 1.90E-08 |
| Neurological Disease | 1.90E-08 |
| Skeletal and Muscular Disorders | 1.90E-08 |
| Post-Translational Modification | 1.56E-05 |
| Nervous System Development and Function | 1.92E-05 |
| Organ Morphology | 1.92E-05 |
| Cell Death and Survival | 3.57E-05 |
| Cellular Assembly and Organization | 5.49E-05 |
| RNA Post-Transcriptional Modification | 5.49E-05 |
| Behavior | 5.58E-05 |
| Cell Morphology | 6.81E-05 |
| Cell-To-Cell Signaling and Interaction | 1.21E-04 |
| Cellular Development | 2.19E-04 |
| Cardiovascular System Development and Function | 2.97E-04 |
| Embryonic Development | 2.97E-04 |
| Organismal Development | 2.97E-04 |
| Tissue Development | 2.97E-04 |
| Tissue Morphology | 2.97E-04 |
| Cellular Movement | 4.23E-04 |
| Cancer | 4.97E-04 |
| Reproductive System Disease | 4.97E-04 |
| Cellular Function and Maintenance | 5.41E-04 |
| Organismal Functions | 5.74E-04 |
| Digestive System Development and Function | 8.12E-04 |
| Endocrine System Development and Function | 8.12E-04 |
| Hematological Disease | 9.63E-04 |
| Immunological Disease | 9.63E-04 |
| Inflammatory Disease | 9.63E-04 |
| Inflammatory Response | 9.63E-04 |
| Respiratory Disease | 9.63E-04 |
| Cell Cycle | 9.97E-04 |
| Connective Tissue Development and Function | 9.97E-04 |
| Connective Tissue Disorders | 1.28E-03 |
| Gene Expression | 1.32E-03 |
| Carbohydrate Metabolism | 1.62E-03 |
| Protein Degradation | 1.62E-03 |
| Protein Synthesis | 1.62E-03 |
| Cellular Compromise | 1.74E-03 |
| Nutritional Disease | 1.74E-03 |
| Organ Development | 1.74E-03 |
| Organismal Injury and Abnormalities | 1.74E-03 |
| Ophthalmic Disease | 1.99E-03 |
| Molecular Transport | 2.27E-03 |
| Small Molecule Biochemistry | 2.27E-03 |
| Cellular Growth and Proliferation | 2.87E-03 |
| Dermatological Diseases and Conditions | 2.87E-03 |
| Hematological System Development and Function | 2.87E-03 |
| Hematopoiesis | 2.87E-03 |
| Lipid Metabolism | 2.98E-03 |
| Lymphoid Tissue Structure and Development | 2.98E-03 |
| Vitamin and Mineral Metabolism | 2.98E-03 |
| Metabolic Disease | 4.02E-03 |
| Visual System Development and Function | 4.67E-03 |
| Energy Production | 5.25E-03 |
| Reproductive System Development and Function | 5.25E-03 |
| Skeletal and Muscular System Development and Function | 5.32E-03 |
| Hair and Skin Development and Function | 6.52E-03 |
| Psychological Disorders | 8.10E-03 |
| Developmental Disorder | 1.33E-02 |
| Cardiovascular Disease | 1.46E-02 |
| Amino Acid Metabolism | 1.73E-02 |
| Cell-mediated Immune Response | 1.73E-02 |
| Endocrine System Disorders | 1.73E-02 |
| Gastrointestinal Disease | 1.73E-02 |
| Immune Cell Trafficking | 1.73E-02 |
| Infectious Disease | 1.73E-02 |
| Renal and Urological Disease | 1.73E-02 |
| Renal and Urological System Development and Function | 1.73E-02 |
