# Supplementary material for: Textrous!: Extracting Semantic Textual Meaning from Gene Sets
Source: PLoS One. 2013 Apr 30;8(4):e62665. doi: 10.1371/journal.pone.0062665 (PMC3639949; doi:10.1371/journal.pone.0062665)
Supplement: Table S8 — NIH DAVID PIR Keyword enrichment output for learning task-oriented activity. The batch gene annotation module of NIH DAVID (http://david.abcc.ncifcrf.gov/) was employed for the derivation of the fold enrichment (using a murine background set) and the significant P value (<0.05) for the specifically enriched PIR keywords extracted from the learning oriented-task transcriptomic dataset. (DOC) [file pone.0062665.s009.doc]

**Table S8. NIH DAVID PIR Keyword enrichment output for learning task-oriented activity.** The batch gene annotation module of NIH DAVID (http://david.abcc.ncifcrf.gov/) was employed for the derivation of the fold enrichment (using a murine background set) and the significant P value (<0.05) for the specifically enriched PIR keywords extracted from the learning oriented-task transcriptomic dataset.

| **PIR Keyword** | **P Value** | **Fold Enrichment** |
| --- | --- | --- |
| cytoplasm | 2.60E-05 | 1.5 |
| phosphoprotein | 9.90E-05 | 1.3 |
| acetylation | 3.00E-04 | 1.5 |
| ubl conjugation | 3.60E-04 | 2.3 |
| nucleotide-binding | 4.80E-03 | 1.5 |
| mitochondrion inner membrane | 5.20E-03 | 3.1 |
| cytoskeleton | 5.80E-03 | 2 |
| isopeptide bond | 6.10E-03 | 2.5 |
| transport | 1.20E-02 | 1.5 |
| atp-binding | 1.70E-02 | 1.5 |
| synapse | 1.90E-02 | 2.5 |
| methylation | 2.50E-02 | 2.4 |
| ubl conjugation pathway | 3.00E-02 | 1.8 |
| endoplasmic reticulum | 3.10E-02 | 1.6 |
| translocation | 3.20E-02 | 4.2 |
| protein transport | 4.70E-02 | 1.8 |
