# Supplementary material for: Textrous!: Extracting Semantic Textual Meaning from Gene Sets
Source: PLoS One. 2013 Apr 30;8(4):e62665. doi: 10.1371/journal.pone.0062665 (PMC3639949; doi:10.1371/journal.pone.0062665)
Supplement: Table S9 — IPA Canonical signaling pathway enrichment output for learning task-oriented activity. Ingenuity Pathway Analysis (IPA: http://www.ingenuity.com/products/ipa) was employed to generate specific Canonical Signaling Pathway activity output from the murine learning oriented-task transcriptomic dataset. The specific significant negative log10 of the P value, as well as the enrichment ratio for each of the significantly-populated Canonical signaling pathways is indicated. (DOC) [file pone.0062665.s010.doc]

**Table S9. IPA Canonical signaling pathway enrichment output for learning task-oriented activity.** Ingenuity Pathway Analysis (IPA: http://www.ingenuity.com/products/ipa) was employed to generate specific Canonical Signaling Pathway activity output from the murine learning oriented-task transcriptomic dataset. The specific significant negative log_10_ of the P value, as well as the enrichment ratio for each of the significantly-populated Canonical signaling pathways is indicated.

| **Canonical Pathways** | **-log(P value)** | **Ratio** |
| --- | --- | --- |
| ILK Signaling | 3.42E00 | 5.73E-02 |
| Remodeling of Epithelial Adherens Junctions | 2.95E00 | 8.82E-02 |
| Androgen Biosynthesis | 2.79E00 | 1.15E-01 |
| Epithelial Adherens Junction Signaling | 2.43E00 | 5.44E-02 |
| Calcium Signaling | 2.42E00 | 4.27E-02 |
| Actin Cytoskeleton Signaling | 2.4E00 | 4.2E-02 |
| Protein Ubiquitination Pathway | 2.31E00 | 4.1E-02 |
| Cyclins and Cell Cycle Regulation | 1.93E00 | 5.62E-02 |
| Mineralocorticoid Biosynthesis | 1.91E00 | 9.52E-02 |
| Regulation of Actin-based Motility by Rho | 1.86E00 | 5.62E-02 |
| Prostate Cancer Signaling | 1.86E00 | 5.1E-02 |
| Glucocorticoid Biosynthesis | 1.83E00 | 9.52E-02 |
| ERK/MAPK Signaling | 1.81E00 | 3.88E-02 |
| CDK5 Signaling | 1.76E00 | 5.32E-02 |
| PI3K/AKT Signaling | 1.7E00 | 4.17E-02 |
| Cholesterol Biosynthesis I | 1.69E00 | 5E-02 |
| Cholesterol Biosynthesis II (via 24,25-dihydrolanosterol) | 1.69E00 | 5E-02 |
| Cholesterol Biosynthesis III (via Desmosterol) | 1.69E00 | 5E-02 |
| Dopamine-DARPP32 Feedback in cAMP Signaling | 1.67E00 | 3.83E-02 |
| Cell Cycle Regulation by BTG Family Proteins | 1.65E00 | 8.33E-02 |
| Cellular Effects of Sildenafil (Viagra) | 1.64E00 | 4.08E-02 |
| Non-Small Cell Lung Cancer Signaling | 1.59E00 | 5.06E-02 |
| Hypoxia Signaling in the Cardiovascular System | 1.59E00 | 6.06E-02 |
| IGF-1 Signaling | 1.58E00 | 4.76E-02 |
| Wnt/β-catenin Signaling | 1.55E00 | 4.02E-02 |
| Telomerase Signaling | 1.53E00 | 4.85E-02 |
| Thyroid Cancer Signaling | 1.5E00 | 7.14E-02 |
| Rac Signaling | 1.48E00 | 4.1E-02 |
| Dopamine Receptor Signaling | 1.39E00 | 4.26E-02 |
| GADD45 Signaling | 1.38E00 | 9.09E-02 |
| Regulation of eIF4 and p70S6K Signaling | 1.37E00 | 3.45E-02 |
| Corticotropin Releasing Hormone Signaling | 1.36E00 | 3.68E-02 |
| Aldosterone Signaling in Epithelial Cells | 1.31E00 | 3.57E-02 |
| Tight Junction Signaling | 1.3E00 | 3.73E-02 |
| RhoA Signaling | 1.28E00 | 4.17E-02 |
| Polyamine Regulation in Colon Cancer | 1.26E00 | 6.9E-02 |
| Synaptic Long Term Potentiation | 1.26E00 | 3.88E-02 |
| Cdc42 Signaling | 1.23E00 | 3.39E-02 |
| Endometrial Cancer Signaling | 1.22E00 | 5.26E-02 |
