# Supplementary material for: Textrous!: Extracting Semantic Textual Meaning from Gene Sets
Source: PLoS One. 2013 Apr 30;8(4):e62665. doi: 10.1371/journal.pone.0062665 (PMC3639949; doi:10.1371/journal.pone.0062665)
Supplement: Table S10 — Parathyroid hormone (hPTH (1–34))-induced bone transcription response in wild-type mice. The transcriptomic response data indicates the significantly regulated genes expressed in calvarial bone extracts from mice intermittently dosed with hPTH (1–34). (DOC) [file pone.0062665.s011.doc]

**Table S10. Parathyroid hormone (hPTH (1-34))-induced bone transcription response in wild-type mice.** The transcriptomic response data indicates the significantly regulated genes expressed in calvarial bone extracts from mice intermittently dosed with hPTH (1-34).

| **Gene Description** | **Gene Symbol** | **Fold-Change Expression** |
| --- | --- | --- |
| phosphate regulating gene with homologies to endopeptidases on the X chromosome | Phex | 4.71166 |
| Wnt inhibitory factor 1 | Wif1 | 4.05855 |
| special AT-rich sequence binding protein 2 | Satb2 | 3.96997 |
| cell growth regulator with EF hand domain 1 | Cgref1 | 3.89926 |
| RNA binding motif protein 45 | Rbm45 | 3.86479 |
| collagen, type XXIV, alpha 1 | Col24a1 | 3.61237 |
| collagen, type XI, alpha 1 | Col11a1 | 3.49771 |
| RAS-like, estrogen-regulated, growth-inhibitor | Rerg | 3.40664 |
| aldehyde dehydrogenase 1 family, member L2 | Aldh1l2 | 3.32607 |
| collagen, type XXII, alpha 1 | Col22a1 | 3.28885 |
| DNA segment, Chr 13, ERATO Doi 787, expressed | D13Ertd787e | 3.13762 |
| pyrroline-5-carboxylate reductase 1 | Pycr1 | 3.03512 |
| lipase, hepatic | Lipc | 3.03131 |
| matrix extracellular phosphoglycoprotein with ASARM motif (bone) | Mepe | 2.9983 |
| FERM, RhoGEF and pleckstrin domain protein 2 | Farp2 | 2.99776 |
| serine (or cysteine) peptidase inhibitor, clade H, member 1 | Serpinh1 | 2.95498 |
| FK506 binding protein 11 | Fkbp11 | 2.95072 |
| pannexin 3 | Panx3 | 2.8097 |
| dickkopf homolog 1 (Xenopus laevis) | Dkk1 | 2.7897 |
| cadherin 2 | Cdh2 | 2.77666 |
| REST corepressor 3 | Rcor3 | 2.76027 |
| interferon induced transmembrane protein 5 | Ifitm5 | 2.74929 |
| protocadherin beta 9 | Pcdhb9 | 2.74635 |
| a disintegrin and metallopeptidase domain 12 (meltrin alpha) | Adam12 | 2.71606 |
| collagen, type XI, alpha 2 | Col11a2 | 2.59785 |
| dentin matrix protein 1 | Dmp1 | 2.59235 |
| solute carrier family 13 (sodium-dependent citrate transporter), member 5 | Slc13a5 | 2.58479 |
| Sp7 transcription factor 7 | Sp7 | 2.55762 |
| fidgetin | Fign | 2.51465 |
| collagen, type I, alpha 2 | Col1a2 | 2.46669 |
| RAN binding protein 3-like | Ranbp3l | 2.42729 |
| CD109 antigen | Cd109 | 2.41716 |
| T-box 2 | Tbx2 | 2.41018 |
| FAT tumor suppressor homolog 3 (Drosophila) | Fat3 | 2.39596 |
| sphingomyelin phosphodiesterase 3, neutral | Smpd3 | 2.32721 |
| proprotein convertase subtilisin/kexin type 6 | Pcsk6 | 2.32285 |
| FK506 binding protein 7 | Fkbp7 | 2.31697 |
| Kazal-type serine peptidase inhibitor domain 1 | Kazald1 | 2.31668 |
| protein tyrosine phosphatase, receptor type Z, polypeptide 1 | Ptprz1 | 2.28387 |
| cyclin B1 interacting protein 1 | Ccnb1ip1 | 2.27818 |
| protocadherin 7 | Pcdh7 | 2.24449 |
| rhophilin, Rho GTPase binding protein 2 | Rhpn2 | 2.24423 |
| mannose receptor, C type 2 | Mrc2 | 2.24308 |
| ectonucleoside triphosphate diphosphohydrolase 3 | Entpd3 | 2.24281 |
| bone gamma-carboxyglutamate protein, related sequence 1 | Bglap1 | 2.22978 |
| potassium channel tetramerisation domain containing 12b | Kctd12b | 2.22355 |
| phosphate regulating gene with homologies to endopeptidases on the X chromosome | Phex | 2.2233 |
| Tenascin C | Tnc | 2.21952 |
| heat shock protein 1B | Hspa1b | 2.21921 |
| collagen triple helix repeat containing 1 | Cthrc1 | 2.21466 |
| FK506 binding protein 10 | Fkbp10 | 2.21315 |
| BMP and activin membrane-bound inhibitor, homolog (Xenopus laevis) | Bambi | 2.20673 |
| solute carrier family 36 (proton/amino acid symporter), member 2 | Slc36a2 | 2.20282 |
| Ca2+-dependent activator protein for secretion 2 | Cadps2 | 2.19952 |
| Retinoic acid induced 14 | Rai14 | 2.19232 |
| WNT1 inducible signaling pathway protein 1 | Wisp1 | 2.18801 |
| membrane associated guanylate kinase, WW and PDZ domain containing 2 | Magi2 | 2.17214 |
| cysteine rich protein 61 | Cyr61 | 2.16723 |
| KDEL (Lys-Asp-Glu-Leu) endoplasmic reticulum protein retention receptor 3 | Kdelr3 | 2.16712 |
| tetraspanin 6 | Tspan6 | 2.16623 |
| cartilage associated protein | Crtap | 2.14902 |
| alkaline phosphatase, liver/bone/kidney | Alpl | 2.14592 |
| procollagen lysine, 2-oxoglutarate 5-dioxygenase 2 | Plod2 | 2.13655 |
| transmembrane protein 87A | Tmem87a | 2.1226 |
| ectonucleotide pyrophosphatase/phosphodiesterase 6 | Enpp6 | 2.1193 |
| cAMP responsive element binding protein 3-like 1 | Creb3l1 | 2.11925 |
| sphingomyelin synthase 2 | Sgms2 | 2.11093 |
| chimerin (chimaerin) 1 | Chn1 | 2.11053 |
| procollagen-proline, 2-oxoglutarate 4-dioxygenase (proline 4-hydroxylase), alpha | P4ha1 | 2.10771 |
| family with sequence similarity 167, member A | Fam167a | 2.10235 |
| DNA segment, human D4S114 | D0H4S114 | 2.09904 |
| cell adhesion molecule 1 | Cadm1 | 2.08442 |
| sphingomyelin phosphodiesterase 3, neutral | Smpd3 | 2.07699 |
| reticulocalbin 3, EF-hand calcium binding domain | Rcn3 | 2.07252 |
| procollagen C-endopeptidase enhancer protein | Pcolce | 2.06648 |
| CD200 antigen | Cd200 | 2.05822 |
| myosin IB | Myo1b | 2.05816 |
| zinc finger homeodomain 4 | Zfhx4 | 2.05648 |
| collagen, type XIII, alpha 1 | Col13a1 | 2.05094 |
| calcium channel, voltage-dependent, beta 3 subunit | Cacnb3 | 2.04872 |
| serine (or cysteine) peptidase inhibitor, clade F, member 1 | Serpinf1 | 2.04349 |
| Na+/H+ exchanger domain containing 2 | Nhedc2 | 2.03545 |
| carbonic anhydrase 3 | Car3 | 2.02058 |
| nuclear factor of activated T-cells, cytoplasmic, calcineurin-dependent 1 | Nfatc1 | 2.01039 |
| cysteine rich protein 61 | Cyr61 | 2.00735 |
| reticulocalbin 1 | Rcn1 | 2.00625 |
| retinol binding protein 4, plasma | Rbp4 | 2.00593 |
| Embryonal Fyn-associated substrate | Efs | 1.99336 |
| gene model 266, (NCBI) | Gm266 | 1.99135 |
| coatomer protein complex, subunit zeta 2 | Copz2 | 1.99126 |
| FK506 binding protein 14 | Fkbp14 | 1.98447 |
| trophoblast glycoprotein | Tpbg | 1.96811 |
| bone morphogenetic protein 8a | Bmp8a | 1.96278 |
| protocadherin 18 | Pcdh18 | 1.95842 |
| germ cell-specific gene 2 | Gsg2 | 1.95689 |
| meteorin, glial cell differentiation regulator-like | Metrnl | 1.95182 |
| leucine rich repeat containing 16A | Lrrc16a | 1.94124 |
| leukemia inhibitory factor receptor | Lifr | 1.94113 |
| a disintegrin-like and metallopeptidase (reprolysin type) with thrombospondin type 1 motif, 9 | Adamts9 | 1.93628 |
| paired related homeobox 1 | Prrx1 | 1.93628 |
| integrin alpha 9 | Itga9 | 1.93403 |
| syndecan 2 | Sdc2 | 1.93351 |
| CLIP associating protein 1 | Clasp1 | 1.92325 |
| collagen, type V, alpha 1 | Col5a1 | 1.91717 |
| sushi-repeat-containing protein, X-linked 2 | Srpx2 | 1.90926 |
| bone morphogenetic protein 1 | Bmp1 | 1.90652 |
| peptidylglycine alpha-amidating monooxygenase | Pam | 1.90415 |
| extracellular matrix protein 1 | Ecm1 | 1.89452 |
| glutathione peroxidase 7 | Gpx7 | 1.88999 |
| glypican 6 | Gpc6 | 1.88696 |
| dynamin 3, opposite strand | Dnm3os | 1.88073 |
| melanoma antigen, family D, 2 | Maged2 | 1.8766 |
| solute carrier family 2 (facilitated glucose transporter), member 13 | Slc2a13 | 1.86687 |
| par-6 partitioning defective 6 homolog gamma (C. elegans) | Pard6g | 1.86131 |
| myocyte enhancer factor 2C | Mef2c | 1.84732 |
| MAM domain containing 2 | Mamdc2 | 1.84419 |
| sema domain, immunoglobulin domain (Ig), short basic domain, secreted, (semaphorin) 3D | Sema3d | 1.84416 |
| Rab40b, member RAS oncogene family | Rab40b | 1.8419 |
| leprecan 1 | Lepre1 | 1.84101 |
| formin homology 2 domain containing 3 | Fhod3 | 1.83505 |
| ST8 alpha-N-acetyl-neuraminide alpha-2,8-sialyltransferase 6 | St8sia6 | 1.83456 |
| suppressor of cytokine signaling 2 | Socs2 | 1.83336 |
| phospholipase A2, group V | Pla2g5 | 1.83332 |
| G protein-coupled receptor 137B | Gpr137b | 1.83079 |
| C-type lectin domain family 11, member a | Clec11a | 1.82959 |
| zinc finger and BTB domain containing 7C | Zbtb7c | 1.82782 |
| matrix metallopeptidase 16 | Mmp16 | 1.82762 |
| histone cluster 1, H3b | Hist1h3b | 1.82747 |
| transmembrane protein 119 | Tmem119 | 1.82703 |
| solute carrier family 37 (glycerol-3-phosphate transporter), member 2 | Slc37a2 | 1.82595 |
| matrix metallopeptidase 23 | Mmp23 | 1.82543 |
| acid phosphatase 5, tartrate resistant | Acp5 | 1.82467 |
| FERM domain containing 4B | Frmd4b | 1.82284 |
| zinc finger protein 36, C3H type-like 1 | Zfp36l1 | 1.81561 |
| unc-5 homolog B (C. elegans) | Unc5b | 1.80912 |
| guanine nucleotide binding protein (G protein), alpha inhibiting 1 | Gnai1 | 1.80799 |
| carbonic anyhydrase 12 | Car12 | 1.80561 |
| Iroquois related homeobox 5 (Drosophila) | Irx5 | 1.80138 |
| carboxypeptidase Z | Cpz | 1.80131 |
| hephaestin | Heph | 1.79851 |
| inscuteable homolog (Drosophila) | Insc | 1.79756 |
| zinc finger protein of the cerebellum 3 | Zic3 | 1.79413 |
| engrailed 1 | En1 | 1.79279 |
| secreted acidic cysteine rich glycoprotein | Sparc | 1.79146 |
| Kruppel-like factor 12 | Klf12 | 1.78128 |
| inhibitor of DNA binding 4 | Id4 | 1.77822 |
| human immunodeficiency virus type I enhancer binding protein 2 | Hivep2 | 1.77741 |
| melanoma antigen, family D, 1 | Maged1 | 1.77675 |
| transmembrane and tetratricopeptide repeat containing 2 | Tmtc2 | 1.77275 |
| galanin | Gal | 1.77242 |
| SWI/SNF related, matrix associated, actin dependent regulator of chromatin, subf | Smarca1 | 1.76055 |
| retinitis pigmentosa GTPase regulator | Rpgr | 1.76051 |
| slit homolog 2 (Drosophila) | Slit2 | 1.75961 |
| rhomboid, veinlet-like 2 (Drosophila) | Rhbdl2 | 1.75683 |
| poly(A) binding protein, cytoplasmic 4-like | Pabpc4l | 1.75422 |
| LIM domain containing preferred translocation partner in lipoma | Lpp | 1.75007 |
| sema domain, seven thrombospondin repeats (type 1 and type 1-like), transmembrane domain (TM) and short cytoplasmic domain, (semaphorin) 5A | Sema5a | 1.74652 |
| G protein-coupled receptor 177 | Gpr177 | 1.74622 |
| actin filament associated protein 1-like 2 | Afap1l2 | 1.73883 |
| neuropilin 2 | Nrp2 | 1.73801 |
| sprouty protein with EVH-1 domain 1, related sequence | Spred1 | 1.73561 |
| unc-5 homolog C (C. elegans) | Unc5c | 1.73334 |
| junction adhesion molecule 2 | Jam2 | 1.73219 |
| anoctamin 1, calcium activated chloride channel | Ano1 | 1.73169 |
| leucine-rich repeat-containing G protein-coupled receptor 4 | Lgr4 | 1.72966 |
| peptidylprolyl isomerase C | Ppic | 1.72812 |
| platelet-derived growth factor, D polypeptide | Pdgfd | 1.72758 |
| G protein-coupled receptor 68 | Gpr68 | 1.72752 |
| collagen, type V, alpha 2 | Col5a2 | 1.72656 |
| collagen, type VI, alpha 3 | Col6a3 | 1.72409 |
| zinc finger protein 260 | Zfp260 | 1.72357 |
| protocadherin beta 17 | Pcdhb17 | 1.71979 |
| inhibitor of DNA binding 3 | Id3 | 1.71754 |
| SEC16 homolog B (S. cerevisiae) | Sec16b | 1.71526 |
| lysophosphatidic acid receptor 3 | Lpar3 | 1.7148 |
| ATPase, H+ transporting, lysosomal V0 subunit D2 | Atp6v0d2 | 1.71464 |
| UDP-GlcNAc:betaGal beta-1,3-N-acetylglucosaminyltransferase 9 | B3gnt9 | 1.70982 |
| CDK5 regulatory subunit associated protein 1 | Cdk5rap1 | 1.70941 |
| glycosyltransferase 8 domain containing 4 | Glt8d4 | 1.70932 |
| piggyBac transposable element derived 5 | Pgbd5 | 1.70794 |
| erythrocyte protein band 4.1-like 2 | Epb4.1l2 | 1.70197 |
| Hydroxyprostaglandin dehydrogenase 15 (NAD) | Hpgd | 1.70024 |
| KDEL (Lys-Asp-Glu-Leu) endoplasmic reticulum protein retention receptor 2 | Kdelr2 | 1.69857 |
| choline kinase alpha | Chka | 1.6985 |
| vitamin K epoxide reductase complex, subunit 1 | Vkorc1 | 1.69753 |
| vesicle-associated membrane protein 7 | Vamp7 | 1.69662 |
| tissue factor pathway inhibitor 2 | Tfpi2 | 1.6959 |
| solute carrier family 8 (sodium/calcium exchanger), member 3 | Slc8a3 | 1.69355 |
| serine (or cysteine) peptidase inhibitor, clade E, member 2 | Serpine2 | 1.69326 |
| solute carrier family 35 (UDP-galactose transporter), member A2 | Slc35a2 | 1.6917 |
| aldehyde dehydrogenase family 1, subfamily A2 | Aldh1a2 | 1.69058 |
| rhomboid, veinlet-like 3 (Drosophila) | Rhbdl3 | 1.69002 |
| SH3 and PX domains 2B | Sh3pxd2b | 1.68898 |
| lectin, mannose-binding, 1 | Lman1 | 1.68768 |
| pentraxin related gene | Ptx3 | 1.6864 |
| sorbin and SH3 domain containing 2 | Sorbs2 | 1.68482 |
| immunoglobulin superfamily containing leucine-rich repeat | Islr | 1.68196 |
| feline leukemia virus subgroup C cellular receptor family, member 2 | Flvcr2 | 1.6815 |
| Sec24 related gene family, member D (S. cerevisiae) | Sec24d | 1.68148 |
| FAT tumor suppressor homolog 1 (Drosophila) | Fat1 | 1.68032 |
| potassium channel, subfamily K, member 1 | Kcnk1 | 1.67891 |
| gap junction protein, alpha 1 | Gja1 | 1.67814 |
| transmembrane protein 98 | Tmem98 | 1.67539 |
| cathepsin K | Ctsk | 1.65795 |
| poliovirus receptor-related 3 | Pvrl3 | 1.65782 |
| NHS-like 1 | Nhsl1 | 1.65421 |
| histone cluster 2, H3c2 | Hist2h3c2 | 1.65317 |
| histocompatibility 60a | H60a | 1.65287 |
| killer cell lectin-like receptor, subfamily A, member 17 | Klra17 | 1.65226 |
| androgen receptor | Ar | 1.64302 |
| potassium channel tetramerisation domain containing 12 | Kctd12 | 1.64164 |
| claudin 10 | Cldn10 | 1.64102 |
| dihydropyrimidinase-like 3 | Dpysl3 | 1.64055 |
| LIM domain binding 2 | Ldb2 | 1.63961 |
| osteoclast associated receptor | Oscar | 1.6384 |
| solute carrier family 2 (facilitated glucose transporter), member 10 | Slc2a10 | 1.63787 |
| arylsulfatase B | Arsb | 1.63037 |
| sphingosine-1-phosphate receptor 3 | S1pr3 | 1.63037 |
| filamin binding LIM protein 1 | Fblim1 | 1.62251 |
| C1q and tumor necrosis factor related protein 6 | C1qtnf6 | 1.62146 |
| N-acetylglucosamine-1-phosphate transferase, alpha and beta subunits | Gnptab | 1.62078 |
| ubiquitin-conjugating enzyme E2E 2 (UBC4/5 homolog, yeast) | Ube2e2 | 1.61983 |
| ribosomal protein L39-like | Rpl39l | 1.619 |
| cadherin 11 | Cdh11 | 1.61847 |
| guanylate cyclase 1, soluble, alpha 3 | Gucy1a3 | 1.61757 |
| armadillo repeat containing, X-linked 2 | Armcx2 | 1.61746 |
| FK506 binding protein 9 | Fkbp9 | 1.61586 |
| epsin 2 | Epn2 | 1.61412 |
| catenin (cadherin associated protein), delta 1 | Ctnnd1 | 1.61325 |
| estrogen receptor 1 (alpha) | Esr1 | 1.61255 |
| uronyl-2-sulfotransferase | Ust | 1.60865 |
| ephrin B1 | Efnb1 | 1.60746 |
| cerebellar degeneration-related protein 2-like | Cdr2l | 1.60705 |
| gamma-glutamyl carboxylase | Ggcx | 1.60582 |
| distal-less homeobox 3 | Dlx3 | 1.60094 |
| G protein-coupled receptor 153 | Gpr153 | 1.59794 |
| hairy/enhancer-of-split related with YRPW motif 1 | Hey1 | 1.5978 |
| vitamin D receptor | Vdr | 1.5962 |
| epidermal growth factor-containing fibulin-like extracellular matrix protein 2 | Efemp2 | 1.59592 |
| zinc finger with KRAB and SCAN domains 3 | Zkscan3 | 1.59548 |
| zinc finger protein 467 | Zfp467 | 1.59516 |
| deleted in lymphocytic leukemia, 2 | Dleu2 | 1.59405 |
| N-acetylglucosamine-1-phosphate transferase, alpha and beta subunits | Gnptab | 1.59154 |
| fibroblast activation protein | Fap | 1.59015 |
| early growth response 3 | Egr3 | 1.58984 |
| neuromedin B | Nmb | 1.58797 |
| CD2-associated protein | Cd2ap | 1.58757 |
| ubiquitin protein ligase E3A | Ube3a | 1.58732 |
| gene model 22, (NCBI) | Gm22 | 1.5848 |
| Rous sarcoma oncogene | Src | 1.58333 |
| geranylgeranyl diphosphate synthase 1 | Ggps1 | 1.57953 |
| MAD homolog 6 (Drosophila) | Smad6 | 1.57928 |
| apelin receptor | Aplnr | 1.57888 |
| activin A receptor, type 1 | Acvr1 | 1.57707 |
| integral membrane protein 2A | Itm2a | 1.57688 |
| high density lipoprotein (HDL) binding protein | Hdlbp | 1.57683 |
| 5'-nucleotidase domain containing 2 | Nt5dc2 | 1.57664 |
| myosin X | Myo10 | 1.57504 |
| alpha-N-acetylglucosaminidase (Sanfilippo disease IIIB) | Naglu | 1.57415 |
| nuclear receptor subfamily 1, group D, member 2 | Nr1d2 | 1.57407 |
| catenin (cadherin associated protein), beta 1 | Ctnnb1 | 1.56966 |
| death-associated protein kinase 2 | Dapk2 | 1.56642 |
| KN motif and ankyrin repeat domains 1 | Kank1 | 1.56588 |
| FYVE, RhoGEF and PH domain containing 1 | Fgd1 | 1.5631 |
| twist gene homolog 1 (Drosophila) | Twist1 | 1.56 |
| regulator of G-protein signaling 3 | Rgs3 | 1.559 |
| carboxypeptidase E | Cpe | 1.55718 |
| ATPase, H+ transporting, lysosomal V1 subunit B2 | Atp6v1b2 | 1.55684 |
| transforming growth factor, beta receptor I | Tgfbr1 | 1.55549 |
| protein tyrosine phosphatase, receptor type, S | Ptprs | 1.55272 |
| a disintegrin-like and metallopeptidase (reprolysin type) with thrombospondin ty | Adamts2 | 1.5505 |
| guanylate cyclase 1, soluble, alpha 3 | Gucy1a3 | 1.55037 |
| fumarylacetoacetate hydrolase domain containing 2A | Fahd2a | 1.54731 |
| spinster homolog 2 (Drosophila) | Spns2 | 1.54542 |
| storkhead box 2 | Stox2 | 1.54515 |
| Neuron navigator 1 | Nav1 | 1.54482 |
| receptor-like tyrosine kinase | Ryk | 1.54474 |
| protein disulfide isomerase associated 5 | Pdia5 | 1.54282 |
| phospholipase C, delta 1 | Plcd1 | 1.54083 |
| integrin binding sialoprotein | Ibsp | 1.54071 |
| olfactomedin-like 2B | Olfml2b | 1.5393 |
| parathyroid hormone receptor 1 | Pthr1 | 1.53899 |
| retinoic acid receptor, beta | Rarb | 1.53749 |
| RIKEN cDNA E430002G05 gene | E430002G05Rik | 1.53516 |
| procollagen-lysine, 2-oxoglutarate 5-dioxygenase 1 | Plod1 | 1.53496 |
| BTB (POZ) domain containing 7 | Btbd7 | 1.5324 |
| coiled-coil domain containing 80 | Ccdc80 | 1.53233 |
| phosphatidic acid phosphatase type 2 domain containing 1B | Ppapdc1b | 1.53232 |
| killer cell lectin-like receptor, subfamily A, member 18 | Klra18 | 1.5322 |
| integrator complex subunit 2 | Ints2 | 1.53175 |
| cadherin 5 | Cdh5 | 1.53123 |
| frizzled homolog 5 (Drosophila) | Fzd5 | 1.53116 |
| cytochrome b5 domain containing 2 | Cyb5d2 | 1.53048 |
| ubiquitin domain containing 2 | Ubtd2 | 1.52931 |
| glycosyltransferase 8 domain containing 1 | Glt8d1 | 1.52814 |
| ceruloplasmin | Cp | 1.52702 |
| solute carrier family 39 (zinc transporter), member 10 | Slc39a10 | 1.52658 |
| complement component factor h | Cfh | 1.52525 |
| Ras association (RalGDS/AF-6) and pleckstrin homology domains 1 | Raph1 | 1.52506 |
| LSM12 homolog (S. cerevisiae) | Lsm12 | 1.52463 |
| protein tyrosine phosphatase, receptor type, f polypeptide (PTPRF), interacting | Ppfia2 | 1.52437 |
| cDNA sequence X99384 | X99384 | 1.52106 |
| tetraspanin 4 | Tspan4 | 1.52103 |
| zinc finger protein 354C | Zfp354c | 1.51809 |
| EP300 interacting inhibitor of differentiation 1 | Eid1 | 1.51687 |
| SRY-box containing gene 17 | Sox17 | 1.51675 |
| ADP-ribosylation factor 4 | Arf4 | 1.51449 |
| glypican 4 | Gpc4 | 1.51283 |
| G protein-coupled receptor 133 | Gpr133 | 1.51112 |
| latent transforming growth factor beta binding protein 3 | Ltbp3 | 1.51089 |
| collagen, type I, alpha 1 | Col1a1 | 1.50688 |
| cytochrome P450, family 4, subfamily v, polypeptide 3 | Cyp4v3 | 1.50601 |
| vasorin | Vasn | 1.50339 |
| sprouty-related, EVH1 domain containing 2 | Spred2 | 1.50252 |
| G1 to S phase transition 1 | Gspt1 | 1.50227 |
| sodium channel, voltage-gated, type III, alpha | Scn3a | 1.50112 |
| mitogen-activated protein kinase kinase kinase 7 interacting protein 3 | Map3k7ip3 | -1.50257 |
| potassium intermediate/small conductance calcium-activated channel, subfamily N, member 4 | Kcnn4 | -1.50328 |
| DEAD (Asp-Glu-Ala-Asp) box polypeptide 60 | Ddx60 | -1.50575 |
| chromodomain helicase DNA binding protein 8 | Chd8 | -1.51074 |
| transmembrane protein with EGF-like and two follistatin-like domains 2 | Tmeff2 | -1.51665 |
| ribosomal protein S6 kinase, polypeptide 1 | Rps6kb1 | -1.52726 |
| ankyrin repeat and BTB (POZ) domain containing 1 | Abtb1 | -1.53197 |
| solute carrier family 6 (neurotransmitter transporter, GABA), member 13 | Slc6a13 | -1.53564 |
| ficolin A | Fcna | -1.53901 |
| sorting nexin 5 | Snx5 | -1.57347 |
| ring finger protein 128 | Rnf128 | -1.57804 |
| kininogen 1 | Kng1 | -1.5813 |
| sodium channel, voltage-gated, type VIII, alpha | Scn8a | -1.58589 |
| cysteine-rich C-terminal 1 | Crct1 | -1.58696 |
| B-cell leukemia/lymphoma 2 | Bcl2 | -1.59123 |
| microfibrillar associated protein 5 | Mfap5 | -1.59271 |
| ATPase, class I, type 8B, member 4 | Atp8b4 | -1.59426 |
| SWI/SNF related, matrix associated, actin dependent regulator of chromatin, subfamily e, member 1 | Smarce1 | -1.60382 |
| ATP-binding cassette, sub-family D (ALD), member 2 | Abcd2 | -1.60385 |
| poly (ADP-ribose) polymerase family, member 3 | Parp3 | -1.61286 |
| 3-hydroxyanthranilate 3,4-dioxygenase | Haao | -1.62242 |
| annexin A6 | Anxa6 | -1.63816 |
| chemokine (C-C motif) ligand 6 | Ccl6 | -1.65112 |
| transcription termination factor, RNA polymerase I | Ttf1 | -1.6561 |
| similar to T-cell receptor beta-2 chain C region | LOC665506 | -1.67362 |
| suprabasin | Sbsn | -1.70619 |
| apolipoprotein C-II | Apoc2 | -1.70742 |
| 2'-5' oligoadenylate synthetase 3 | Oas3 | -1.71966 |
| TSC22 domain family, member 1 | Tsc22d1 | -1.73313 |
| solute carrier family 6 (neurotransmitter transporter), member 20A | Slc6a20a | -1.73452 |
| acyl-CoA thioesterase 2 | Acot2 | -1.73874 |
| XIAP associated factor 1 | Xaf1 | -1.74037 |
| T cell receptor beta, joining region | Tcrb-J | -1.74298 |
| nephroblastoma overexpressed gene | Nov | -1.77729 |
| leucine zipper transcription factor-like 1 | Lztfl1 | -1.83916 |
| Activating signal cointegrator 1 complex subunit 3-like 1 | Ascc3l1 | -1.84552 |
| BCL2-like 11 (apoptosis facilitator) | Bcl2l11 | -1.84582 |
| metallothionein 2 | Mt2 | -1.92114 |
| CD163 antigen | Cd163 | -1.93717 |
| DEP domain containing 6 | Depdc6 | -1.94935 |
| zinc finger, CCHC domain containing 11 | Zcchc11 | -2.02479 |
| D-2-hydroxyglutarate dehydrogenase | D2hgdh | -2.03238 |
| integrin beta 2-like | Itgb2l | -2.03658 |
| radical S-adenosyl methionine domain containing 2 | Rsad2 | -2.08215 |
| zinc finger protein 39 | Zfp39 | -2.16668 |
| keratinocyte differentiation associated protein | Krtdap | -2.17775 |
| mitochondrial fission factor | Mff | -2.30955 |
| ankyrin repeat domain 1 (cardiac muscle) | Ankrd1 | -2.33276 |
| CD5 antigen-like | Cd5l | -2.39341 |
| RIO kinase 3 (yeast) | Riok3 | -2.53207 |
| nuclear distribution gene E-like homolog 1 (A. nidulans) | Ndel1 | -4.57746 |
