# Supplementary material for: Textrous!: Extracting Semantic Textual Meaning from Gene Sets
Source: PLoS One. 2013 Apr 30;8(4):e62665. doi: 10.1371/journal.pone.0062665 (PMC3639949; doi:10.1371/journal.pone.0062665)
Supplement: Table S11 — Parathyroid hormone variant (bPTH (7–34))-induced bone transcription responses in wild-type mice. The transcriptomic response data indicates the significantly regulated genes expressed in calvarial bone extracts from wild-type mice intermittently dosed with the parathyroid hormone variant bPTH (7–34). (DOC) [file pone.0062665.s012.doc]

**Table S11. Parathyroid hormone variant (bPTH (7-34))-induced bone transcription responses in wild-type mice.** The transcriptomic response data indicates the significantly regulated genes expressed in calvarial bone extracts from wild-type mice intermittently dosed with the parathyroid hormone variant bPTH (7-34).

| **Gene Description** | **Gene Symbol** | **Fold-Change Expression** |
| --- | --- | --- |
| ankyrin repeat and SOCS box-containing 15 | Asb15 | 2.87096 |
| germ cell-specific gene 2 | Gsg2 | 2.55592 |
| neuropeptide Y | Npy | 2.43545 |
| T-cell immunoglobulin and mucin domain containing 4 | Timd4 | 2.36497 |
| E2F transcription factor 8 | E2f8 | 2.20251 |
| tyrosine 3-monooxygenase/tryptophan 5-monooxygenase activation protein, zeta | Ywhaz | 2.01103 |
| RIKEN cDNA 6820431F20 | 6820431F20Rik | 1.98818 |
| angiopoietin-like 2 | Angptl2 | 1.94868 |
| angiopoietin-like 2 | Angptl2 | 1.92492 |
| cyclin B1 interacting protein 1 | Ccnb1ip1 | 1.82268 |
| protease, serine, 35 | Prss35 | 1.81559 |
| suppressor of Ty 16 homolog (S. cerevisiae) | Supt16h | 1.78623 |
| sodium channel, voltage-gated, type II, beta | Scn2b | 1.77891 |
| solute carrier organic anion transporter family, member 4C1 | Slco4c1 | 1.77737 |
| peptidylglycine alpha-amidating monooxygenase | Pam | 1.76729 |
| steroid 5 alpha-reductase 3 | Srd5a3 | 1.76501 |
| tetratricopeptide repeat domain 39A | Ttc39a | 1.74172 |
| hyaluronan mediated motility receptor (RHAMM) | Hmmr | 1.73988 |
| cyclin B1 | Ccnb1 | 1.72099 |
| glutamate receptor, ionotropic, AMPA1 (alpha 1) | Gria1 | 1.6782 |
| hydroxymethylbilane synthase | Hmbs | 1.66568 |
| collagen, type XXV, alpha 1 | Col25a1 | 1.65357 |
| sperm associated antigen 5 | Spag5 | 1.64787 |
| ATP-binding cassette, sub-family B (MDR/TAP), member 10 | Abcb10 | 1.64448 |
| cyclin B1 | Ccnb1 | 1.62986 |
| DNA segment, human D4S114 | D0H4S114 | 1.62365 |
| forkhead box J3 | Foxj3 | 1.61415 |
| RIKEN cDNA 6820431F20 | 6820431F20Rik | 1.60639 |
| RIKEN cDNA 4933404O12 | 4933404O12Rik | 1.60599 |
| ribonucleotide reductase M2 | Rrm2 | 1.59372 |
| Kruppel-like factor 1 (erythroid) | Klf1 | 1.57571 |
| AF4/FMR2 family, member 1 | Aff1 | 1.56928 |
| RIKEN cDNA 2310005G13 | 2310005G13Rik | 1.56191 |
| GTP cyclohydrolase 1 | Gch1 | 1.55831 |
| RIKEN cDNA 4632434I11 | 4632434I11Rik | 1.5512 |
| plexin C1 | Plxnc1 | 1.54933 |
| large tumor suppressor 2 | Lats2 | 1.54795 |
| colony stimulating factor 2 receptor, alpha | Csf2ra | 1.54681 |
| DNA segment, human D4S114 | D0H4S114 | 1.54665 |
| tripartite motif-containing 10 | Trim10 | 1.54339 |
| glutamate receptor, ionotropic, AMPA1 (alpha 1) | Gria1 | 1.54321 |
| transcription factor Dp 2 | Tfdp2 | 1.54178 |
| integrin alpha 9 | Itga9 | 1.5261 |
| ATP-binding cassette, sub-family B (MDR/TAP), member 10 | Abcb10 | 1.51663 |
| ring finger protein 11 | Rnf11 | 1.51145 |
| GTP cyclohydrolase 1 | Gch1 | 1.51114 |
| thioredoxin reductase 1 | Txnrd1 | 1.51041 |
| tetratricopeptide repeat domain 28 | Ttc28 | -1.50147 |
| diaphanous homolog 2 (Drosophila) | Diap2 | -1.50231 |
| SH3 domain protein D19 | Sh3d19 | -1.50358 |
| large tumor suppressor 2 | Lats2 | -1.50945 |
| transmembrane protein 30B | Tmem30b | -1.50966 |
| RIKEN cDNA G530011O06 | G530011O06Rik | -1.51013 |
| sodium channel, voltage-gated, type VII, alpha | Scn7a | -1.51338 |
| RAS-like, family 11, member B | Rasl11b | -1.51415 |
| follistatin-like 1 | Fstl1 | -1.51518 |
| G protein-coupled receptor associated sorting protein 1 | Gprasp1 | -1.5179 |
| synaptopodin | Synpo | -1.52 |
| membrane-spanning 4-domains, subfamily A, member 4D | Ms4a4d | -1.52411 |
| pleckstrin homology domain-containing, family A, member 2 | Plekha2 | -1.52471 |
| forkhead box P1 | Foxp1 | -1.52562 |
| T-cell receptor beta, joining region | Tcrb-J | -1.52658 |
| tumor necrosis factor receptor superfamily, member 12a | Tnfrsf12a | -1.52985 |
| podoplanin | Pdpn | -1.53186 |
| transmembrane protein with EGF-like and two follistatin-like domains 2 | Tmeff2 | -1.53768 |
| transcription factor 7, T-cell specific | Tcf7 | -1.54135 |
| collagen, type V, alpha 3 | Col5a3 | -1.54196 |
| ribosome binding protein 1 | Rrbp1 | -1.5439 |
| DNA segment, Chr 17, ERATO Doi 165, expressed | D17Ertd165e | -1.55134 |
| suprabasin | Sbsn | -1.55206 |
| nuclear protein 1 | Nupr1 | -1.55251 |
| RNA binding motif, single stranded interacting protein | Rbms3 | -1.55377 |
| glutamyl-prolyl-tRNA synthetase | Eprs | -1.55632 |
| cytochrome P450, family 2, subfamily d, polypeptide 22 | Cyp2d22 | -1.55896 |
| protein tyrosine phosphatase, receptor type, G | Ptprg | -1.56027 |
| RIKEN cDNA 0610007N19 | 0610007N19Rik | -1.56416 |
| potassium inwardly-rectifying channel, subfamily J, member 15 | Kcnj15 | -1.56593 |
| leukemia inhibitory factor receptor | Lifr | -1.57094 |
| HtrA serine peptidase 3 | Htra3 | -1.57099 |
| GTPase, IMAP family member 4 | Gimap4 | -1.57157 |
| Autism susceptibility candidate 2 | Auts2 | -1.57238 |
| DEAH (Asp-Glu-Ala-His) box polypeptide 35 | Dhx35 | -1.57438 |
| lipoic acid synthetase | Lias | -1.57755 |
| thymus cell antigen 1, theta | Thy1 | -1.5827 |
| cDNA sequence BC028801 | BC028801 | -1.58295 |
| RIKEN cDNA 9130213B05 | 9130213B05Rik | -1.58312 |
| B-cell leukemia/lymphoma 2 | Bcl2 | -1.58444 |
| RIKEN cDNA 2210012G02 | 2210012G02Rik | -1.58632 |
| RIKEN cDNA 9130213B05 | 9130213B05Rik | -1.58891 |
| B-cell leukemia/lymphoma 11B | Bcl11b | -1.58944 |
| chordin-like 1 | Chrdl1 | -1.59103 |
| nuclear protein 1 | Nupr1 | -1.59139 |
| a disintegrin-like and metallopeptidase (reprolysin type) with thrombospondin type 1 motif, 4 | Adamts4 | -1.59539 |
| RIKEN cDNA D730035F11 | D730035F11Rik | -1.59933 |
| early B-cell factor 1 | Ebf1 | -1.60065 |
| G protein-coupled receptor 124 | Gpr124 | -1.60285 |
| fibrillin 1 | Fbn1 | -1.60708 |
| immunoglobulin heavy variable V14-2 | Ighv14-2 | -1.61626 |
| carboxypeptidase X 2 (M14 family) | Cpxm2 | -1.62123 |
| poly (ADP-ribose) polymerase family, member 3 | Parp3 | -1.62512 |
| ubiquitin associated and SH3 domain containing, B | Ubash3b | -1.62625 |
| GTPase, IMAP family member 3 | Gimap3 | -1.62746 |
| p21 (CDKN1A)-activated kinase 3 | Pak3 | -1.6294 |
| apolipoprotein C-II | Apoc2 | -1.63011 |
| WNT1 inducible signaling pathway protein 2 | Wisp2 | -1.63014 |
| neuronal PAS domain protein 2 | Npas2 | -1.63374 |
| RIKEN cDNA C130098B18 | C130098B18Rik | -1.63941 |
| activating transcription factor 3 | Atf3 | -1.64678 |
| ring finger protein 128 | Rnf128 | -1.65384 |
| 2'-5' oligoadenylate synthetase 3 | Oas3 | -1.65447 |
| tripartite motif-containing 30 | Trim30 | -1.65995 |
| claudin 8 | Cldn8 | -1.66198 |
| Sorting nexin 10 | Snx10 | -1.66627 |
| RIKEN cDNA C230085N15 | C230085N15Rik | -1.6696 |
| glutamyl-prolyl-tRNA synthetase | Eprs | -1.67084 |
| solute carrier family 47, member 1 | Slc47a1 | -1.67191 |
| insulin-like growth factor binding protein 3 | Igfbp3 | -1.67299 |
| BCL2-like 11 (apoptosis facilitator) | Bcl2l11 | -1.68132 |
| interleukin 33 | Il33 | -1.68206 |
| chemokine (C-X-C motif) receptor 7 | Cxcr7 | -1.68252 |
| TSC22 domain family, member 1 | Tsc22d1 | -1.68292 |
| LIM domains containing 1 | Limd1 | -1.68332 |
| T cell receptor beta, joining region | Tcrb-J | -1.69002 |
| epidermal growth factor-containing fibulin-like extracellular matrix protein 1 | Efemp1 | -1.69341 |
| sodium channel, voltage-gated, type VII, alpha | Scn7a | -1.69635 |
| zinc finger homeobox 3 | Zfhx3 | -1.70056 |
| scavenger receptor class A, member 5 (putative) | Scara5 | -1.7045 |
| vestigial like 3 (Drosophila) | Vgll3 | -1.70493 |
| suprabasin | Sbsn | -1.70559 |
| collagen, type IV, alpha 1 | Col4a1 | -1.70785 |
| actin, alpha 2, smooth muscle, aorta | Acta2 | -1.70917 |
| plasminogen activator, tissue | Plat | -1.71112 |
| transgelin | Tagln | -1.71976 |
| leucyl-tRNA synthetase, mitochondrial | Lars2 | -1.72309 |
| microfibrillar associated protein 5 | Mfap5 | -1.72807 |
| chromodomain helicase DNA binding protein 8 | Chd8 | -1.72979 |
| solute carrier family 4, sodium bicarbonate cotransporter, member 7 | Slc4a7 | -1.73816 |
| vestigial like 3 (Drosophila) | Vgll3 | -1.74038 |
| similar to T-cell receptor beta-2 chain C region | LOC665506 | -1.74874 |
| RIKEN cDNA 3021401C12 | 3021401C12Rik | -1.75382 |
| adaptor-related protein complex 2, beta 1 subunit | Ap2b1 | -1.75442 |
| glutamyl aminopeptidase | Enpep | -1.76178 |
| TM2 domain containing 2 | Tm2d2 | -1.76181 |
| expressed sequence C80258 | C80258 | -1.77182 |
| Fc receptor, IgE, low affinity II, alpha polypeptide | Fcer2a | -1.77886 |
| RIKEN cDNA 1810015C04 gene | 1810015C04Rik | -1.78481 |
| cell adhesion molecule-related/down-regulated by oncogenes | Cdon | -1.79032 |
| nebulette | Nebl | -1.8188 |
| nicotinamide N-methyltransferase | Nnmt | -1.82334 |
| zyg-ll homolog B (C. elegans) | Zyg11b | -1.82841 |
| tumor necrosis factor receptor superfamily, member 12a | Tnfrsf12a | -1.83095 |
| kinesin family member 21A | Kif21a | -1.83612 |
| T cell receptor beta, joining region | Tcrb-J | -1.86405 |
| membrane-associated ring finger (C3HC4) 6 | March6 | -1.87113 |
| ribosomal protein S25 | Rps25 | -1.8781 |
| apolipoprotein D | Apod | -1.88246 |
| Esterase D/formylglutathione hydrolase | Esd | -1.90384 |
| phosphopantothenoylcysteine decarboxylase | Ppcdc | -1.93065 |
| ELAV (embryonic lethal, abnormal vision, Drosophila)-like 1 (Hu antigen R) | Elavl1 | -1.94339 |
| similar to T-cell receptor beta-2 chain C region | LOC665506 | -1.955 |
| membrane-spanning 4-domains, subfamily A, member 4B | Ms4a4b | -1.9613 |
| cyclin-dependent kinase inhibitor 1A (P21) | Cdkn1a | -1.97213 |
| histocompatibility 2, Q region locus 10 | H2-Q10 | -1.98893 |
| ankyrin repeat domain 1 (cardiac muscle) | Ankrd1 | -1.99451 |
| LOC403343 | 130004C03 | -1.99531 |
| septin 14 | Sept14 | -2.01321 |
| annexin A6 | Anxa6 | -2.01694 |
| DEP domain containing 6 | Depdc6 | -2.0238 |
| apolipoprotein D | Apod | -2.03343 |
| leucine zipper transcription factor-like 1 | Lztfl1 | -2.08228 |
| RIKEN cDNA D130051D11 | D130051D11Rik | -2.11416 |
| BTB and CNC homology 2 | Bach2 | -2.13539 |
| lysyl oxidase | Lox | -2.14505 |
| nephroblastoma overexpressed gene | Nov | -2.23761 |
| nephroblastoma overexpressed gene | Nov | -2.31564 |
| xin actin-binding repeat containing 1 | Xirp1 | -2.31964 |
| dysferlin interacting protein 1 | Dysfip1 | -2.47285 |
| ataxia telangiectasia mutated homolog (human) | Atm | -2.49957 |
| RIKEN cDNA 2310076L09 gene | 2310076L09Rik | -2.54546 |
| RIKEN cDNA 2310050P20 gene | 2310050P20Rik | -2.60405 |
| lectin, galactose binding, soluble 7 | Lgals7 | -2.66571 |
| ankyrin repeat and SOCs box-containing 5 | Asb5 | -2.80108 |
| RIKEN cDNA 2310076L09 gene | 2310076L09Rik | -2.8101 |
| zinc finger, CCHC domain containing 11 | Zcchc11 | -2.97615 |
| RIKEN cDNA 5730557B15 gene | 5730557B15Rik | -3.43097 |
| CDNA sequence BC031748 | BC031748 | -3.63349 |
| angiogenin, ribonuclease, RNase A family, 5 | Ang | -3.65652 |
| nuclear distribution gene E-like homolog 1 (A. nidulans) | Ndel1 | -5.008 |
| retinitis pigmentosa GTPase regulator interacting protein 1 | Rpgrip1 | -6.6466 |
| palate, lung, and nasal epithelium associated | Plunc | -6.8373 |
| hypoxanthine guanine phosphoribosyl transferase 1 | Hprt1 | -10.2881 |
| angiogenin, ribonuclease, RNase A family, 5 | Ang | -11.0878 |
