# Supplementary material for: Textrous!: Extracting Semantic Textual Meaning from Gene Sets
Source: PLoS One. 2013 Apr 30;8(4):e62665. doi: 10.1371/journal.pone.0062665 (PMC3639949; doi:10.1371/journal.pone.0062665)
Supplement: Table S12 — Textrous! output from hPTH (1–34) calvarial bone transcription response in wild-type mice. The Cosine similarity, Z-scores and associated P values for the word data output (top 100) from hPTH (1–34)-treated mice is indicated in the table. (DOC) [file pone.0062665.s013.doc]

**Table S12. *Textrous!* output from hPTH (1-34) calvarial bone transcription response in wild-type mice.** The Cosine similarity, Z-scores and associated P values for the word data output (top 100) from hPTH (1-34)-treated mice is indicated in the table.

| **Word** | **Cosine Similarity** | **Z-Score** | **P Value** |
| --- | --- | --- | --- |
| adherens | 0.593309429 | 2.733649797 | 0.003128502 |
| alpha-catenin | 0.587621872 | 2.707576181 | 0.003384501 |
| gamma-catenin | 0.526720547 | 2.428384644 | 0.007591172 |
| catenin | 0.512354452 | 2.362525782 | 0.009063835 |
| tilt | 0.494475221 | 2.280561555 | 0.011274223 |
| plakoglobin | 0.490989743 | 2.26458299 | 0.01175634 |
| n-cadherin | 0.490418913 | 2.261966118 | 0.011848702 |
| cadherin | 0.490030438 | 2.26018522 | 0.011910625 |
| catenins | 0.473624645 | 2.184975713 | 0.014444423 |
| cadherins | 0.470742919 | 2.171764946 | 0.014927831 |
| e-cadherin | 0.462175031 | 2.132486952 | 0.016503423 |
| p-cadherin | 0.421215771 | 1.944716346 | 0.025887489 |
| beta-catenin | 0.415929253 | 1.920481223 | 0.02742895 |
| zonula | 0.383463161 | 1.77164607 | 0.038197279 |
| dermal | 0.383069707 | 1.769842347 | 0.03836357 |
| calvaria | 0.379265699 | 1.752403536 | 0.039886904 |
| blastomeres | 0.375074221 | 1.733188433 | 0.041547834 |
| wolffian | 0.373052644 | 1.723920865 | 0.042353922 |
| myoepithelial | 0.352480163 | 1.629609903 | 0.051550748 |
| osteocyte | 0.350906187 | 1.622394286 | 0.052401674 |
| osteocytes | 0.349511216 | 1.615999281 | 0.053047157 |
| mineral | 0.34870495 | 1.612303094 | 0.05348097 |
| fracture | 0.346487008 | 1.602135328 | 0.054577805 |
| fractures | 0.345781321 | 1.598900227 | 0.054910301 |
| hypotrichosis | 0.344866678 | 1.594707206 | 0.055356118 |
| blood-testis | 0.342239071 | 1.582661395 | 0.056710732 |
| femoral | 0.337922105 | 1.562871019 | 0.059026297 |
| tight | 0.336480051 | 1.556260174 | 0.059854046 |
| hormone-dependent | 0.336378675 | 1.55579543 | 0.059854046 |
| blastomere | 0.333088816 | 1.540713645 | 0.061658393 |
| morula | 0.333008692 | 1.540346329 | 0.061780177 |
| cell-to-cell | 0.332607861 | 1.538508791 | 0.061902148 |
| seminiferous | 0.331995105 | 1.535699715 | 0.06226919 |
| junctions | 0.330154065 | 1.527259791 | 0.063380506 |
| intercalated | 0.328217593 | 1.518382369 | 0.064507199 |
| sertoli | 0.326833842 | 1.512038806 | 0.065266931 |
| osteoporosis | 0.32587406 | 1.507638852 | 0.065777264 |
| emt | 0.324603354 | 1.501813519 | 0.066548554 |
| sclera | 0.323638738 | 1.497391404 | 0.067196629 |
| osteoporotic | 0.319551136 | 1.478652505 | 0.069570157 |
| trabecular | 0.316609297 | 1.465166157 | 0.071460459 |
| ureters | 0.316418039 | 1.464289365 | 0.071596974 |
| periosteum | 0.314929642 | 1.457466067 | 0.07255819 |
| absorptiometry | 0.313922329 | 1.452848217 | 0.073111877 |
| dxa | 0.313808204 | 1.452325031 | 0.073250803 |
| osteopenia | 0.311406277 | 1.441313815 | 0.074792341 |
| limitans | 0.311129126 | 1.440043266 | 0.0749337 |
| mesenchymal | 0.307405796 | 1.422974305 | 0.077368078 |
| sediments | 0.306046171 | 1.41674134 | 0.078241464 |
| dedifferentiation | 0.302156157 | 1.398908247 | 0.080906492 |
| occludens | 0.301553514 | 1.396145535 | 0.081357248 |
| vertebra | 0.300550741 | 1.391548498 | 0.081961199 |
| heterotypic | 0.300411008 | 1.390907917 | 0.082112713 |
| mullerian | 0.300240569 | 1.390126567 | 0.082264439 |
| culture | 0.298667078 | 1.382913173 | 0.083332433 |
| disorganization | 0.298640548 | 1.382791551 | 0.083332433 |
| lapse | 0.298363155 | 1.381519891 | 0.083485851 |
| postmenopausal | 0.297426273 | 1.377224917 | 0.084256124 |
| estrogenic | 0.296747861 | 1.374114855 | 0.084720841 |
| post-menopausal | 0.290929475 | 1.347441478 | 0.088990116 |
| osteogenesis | 0.290209127 | 1.344139164 | 0.089474193 |
| lumbar | 0.289372218 | 1.340302502 | 0.090122672 |
| dedifferentiated | 0.287858153 | 1.333361536 | 0.091265902 |
| fetoplacental | 0.287035767 | 1.329591447 | 0.091759136 |
| procollagen | 0.286035521 | 1.325005994 | 0.092585576 |
| genistein | 0.28601191 | 1.324897755 | 0.092585576 |
| height | 0.285981905 | 1.324760201 | 0.092585576 |
| vertebral | 0.285811652 | 1.323979707 | 0.092751522 |
| estrogen | 0.284551237 | 1.318201553 | 0.093751824 |
| bone | 0.284188407 | 1.316538224 | 0.093919312 |
| ici | 0.282195454 | 1.307401874 | 0.095606356 |
| organogenesis | 0.282084581 | 1.3068936 | 0.095606356 |
| feminization | 0.280786815 | 1.300944214 | 0.096629227 |
| prostates | 0.28012357 | 1.297903685 | 0.097143667 |
| decorin | 0.278763346 | 1.291667972 | 0.098178572 |
| preimplantation | 0.278544047 | 1.290662635 | 0.098351839 |
| androgen | 0.278445813 | 1.2902123 | 0.098525329 |
| beta-estradiol | 0.27834202 | 1.289736476 | 0.098525329 |
| lobules | 0.277693678 | 1.286764268 | 0.099047144 |
| dermis | 0.277218152 | 1.284584301 | 0.099396142 |
| bulbar | 0.275399018 | 1.276244795 | 0.10097776 |
| trans-synaptic | 0.274995775 | 1.2743962 | 0.101331708 |
| banded | 0.274949174 | 1.274182567 | 0.101331708 |
| mechanical | 0.274902135 | 1.273966921 | 0.101331708 |
| spine | 0.27382771 | 1.26904141 | 0.102220532 |
| estradiol | 0.273599777 | 1.267996489 | 0.102398975 |
| myopia | 0.2733342 | 1.266778999 | 0.102577645 |
| collagen | 0.273316895 | 1.266699667 | 0.102577645 |
| dogs | 0.272882442 | 1.264707993 | 0.102935664 |
| uteri | 0.272262163 | 1.26186443 | 0.103474393 |
| dht | 0.271887112 | 1.260145076 | 0.103834681 |
| academic | 0.271463226 | 1.258201843 | 0.104195878 |
| prepubertal | 0.271097926 | 1.256527188 | 0.104376818 |
| subfertility | 0.27097278 | 1.255953477 | 0.104557985 |
| testes | 0.269420187 | 1.248835887 | 0.105832537 |
| bisphosphonate | 0.267481813 | 1.239949749 | 0.107487697 |
| kennedy | 0.266748629 | 1.236588592 | 0.108043541 |
| mar | 0.266227107 | 1.234197765 | 0.108601452 |
| fetuses | 0.265851277 | 1.23247484 | 0.108974542 |
| embedding | 0.265307058 | 1.229979961 | 0.109348552 |
