# Supplementary material for: Textrous!: Extracting Semantic Textual Meaning from Gene Sets
Source: PLoS One. 2013 Apr 30;8(4):e62665. doi: 10.1371/journal.pone.0062665 (PMC3639949; doi:10.1371/journal.pone.0062665)
Supplement: Table S13 — Textrous! output from bPTH (7–34) parathyroid hormone variant calvarial bone transcription responses in wild-type mice. The Cosine similarity, Z-scores and associated P values for the word data output (top 100) from bPTH (7–34)-treated mice is indicated in the table. (DOC) [file pone.0062665.s014.doc]

**Table S13. *Textrous!* output from bPTH (7-34) parathyroid hormone variant calvarial bone transcription responses in wild-type mice.** The Cosine similarity, Z-scores and associated P values for the word data output (top 100) from bPTH (7-34)-treated mice is indicated in the table.

| **Word** | **Cosine Similarity** | **Z-Score** | **P Value** |
| --- | --- | --- | --- |
| cyclin | 0.656744083 | 2.898398523 | 0.001877753 |
| cyclins | 0.59048034 | 2.607173831 | 0.004566968 |
| cyclin-dependent | 0.579988547 | 2.561063105 | 0.005218568 |
| m-phase | 0.530709037 | 2.344482973 | 0.009539086 |
| cdk | 0.515369257 | 2.277065669 | 0.011393113 |
| n-methyl-d-aspartic | 0.46697827 | 2.064390539 | 0.019508858 |
| glutamatergic | 0.461335056 | 2.03958899 | 0.020675163 |
| nmda | 0.460093393 | 2.034131967 | 0.020975798 |
| postsynaptic | 0.45075893 | 1.99310763 | 0.023130724 |
| mitosis | 0.446009147 | 1.972232655 | 0.024304803 |
| n-methyl-d-aspartate | 0.443178934 | 1.959794058 | 0.024997895 |
| ionotropic | 0.433896568 | 1.918998681 | 0.027492167 |
| ampa | 0.427921038 | 1.892736631 | 0.029178935 |
| nmdar | 0.425269989 | 1.881085446 | 0.029985959 |
| synapses | 0.421004896 | 1.862340649 | 0.031301545 |
| potentiation | 0.41899095 | 1.853489494 | 0.031941179 |
| interphase | 0.416515318 | 1.842609256 | 0.032664505 |
| transition | 0.415616895 | 1.83866075 | 0.032957593 |
| long-term | 0.414510954 | 1.833800214 | 0.033326997 |
| mitotic | 0.41178242 | 1.821808491 | 0.034227493 |
| interferences | 0.406798097 | 1.799902724 | 0.035930319 |
| inhibitor | 0.406649321 | 1.799248866 | 0.03600934 |
| propionic | 0.402817651 | 1.78240893 | 0.037374616 |
| synaptic | 0.399312519 | 1.767004115 | 0.038614114 |
| bicuculline | 0.398456016 | 1.763239841 | 0.038950245 |
| metabotropic | 0.396883603 | 1.756329191 | 0.039544204 |
| phases | 0.394103412 | 1.74411044 | 0.040579545 |
| plasticity | 0.393911369 | 1.743266423 | 0.040666807 |
| transmission | 0.393697212 | 1.742325215 | 0.040754222 |
| d-dependent | 0.393138525 | 1.739869825 | 0.040929509 |
| phase | 0.391243744 | 1.731542389 | 0.041636781 |
| excitatory | 0.378720099 | 1.676501812 | 0.046771241 |
| kainate | 0.378338762 | 1.674825862 | 0.046967116 |
| cyca | 0.37539893 | 1.661905496 | 0.048256387 |
| post-synaptic | 0.373577352 | 1.653899783 | 0.049063756 |
| cycle | 0.372502227 | 1.649174681 | 0.049573817 |
| thalamic | 0.370962283 | 1.642406734 | 0.050295002 |
| s-phase | 0.366019753 | 1.620684644 | 0.052508819 |
| kainic | 0.360462317 | 1.596260089 | 0.055244397 |
| herpesviruses | 0.360445547 | 1.596186383 | 0.055244397 |
| ketamine | 0.359993883 | 1.594201353 | 0.055468017 |
| afferents | 0.359012576 | 1.589888574 | 0.055917403 |
| kinase-associated | 0.35589973 | 1.576207823 | 0.057512902 |
| laminae | 0.355369462 | 1.573877333 | 0.057743725 |
| proliferating | 0.355054526 | 1.572493212 | 0.057975276 |
| ota | 0.35500364 | 1.572269569 | 0.057975276 |
| epileptiform | 0.353947348 | 1.567627237 | 0.058440566 |
| brdu | 0.349692045 | 1.548925467 | 0.06069086 |
| centrosome | 0.348139212 | 1.542100871 | 0.061536797 |
| cell-cycle | 0.348088167 | 1.541876532 | 0.061536797 |
| terminals | 0.342973747 | 1.519398999 | 0.064381248 |
| learning | 0.337042203 | 1.493330264 | 0.067718593 |
| hippocampal | 0.336128581 | 1.489314956 | 0.068243684 |
| progression | 0.334856528 | 1.483724366 | 0.06890446 |
| b-raf | 0.332207567 | 1.472082364 | 0.070510439 |
| bursts | 0.332066763 | 1.47146354 | 0.070645558 |
| cytoplasms | 0.328922317 | 1.457643911 | 0.072420272 |
| arrest | 0.324546007 | 1.438410321 | 0.075217027 |
| glutamate | 0.323440572 | 1.433552007 | 0.075786131 |
| righting | 0.32275671 | 1.430546483 | 0.076215108 |
| injection | 0.322656463 | 1.430105902 | 0.07635851 |
| monocular | 0.321931718 | 1.426920699 | 0.076789947 |
| spikes | 0.321289854 | 1.424099748 | 0.077223236 |
| pyramidal | 0.318909794 | 1.413639545 | 0.078680951 |
| poisons | 0.318424005 | 1.411504535 | 0.078974981 |
| deafferentation | 0.316468768 | 1.4029114 | 0.080308419 |
| reflex | 0.314811494 | 1.395627792 | 0.081357248 |
| nephritic | 0.312303873 | 1.384606966 | 0.083026234 |
| proliferation | 0.311511406 | 1.381124127 | 0.08363948 |
| mossy | 0.311330405 | 1.38032864 | 0.083793322 |
| shields | 0.310927578 | 1.378558241 | 0.083947377 |
| morris | 0.310367023 | 1.376094641 | 0.084410817 |
| phrenic | 0.309933941 | 1.374191275 | 0.084720841 |
| scaling | 0.309478687 | 1.372190464 | 0.085031719 |
| hippocampus | 0.309337228 | 1.37156876 | 0.085031719 |
| occluded | 0.308856567 | 1.369456288 | 0.085499637 |
| presynaptic | 0.307144927 | 1.361933746 | 0.086598943 |
| application | 0.30678709 | 1.360361076 | 0.086914962 |
| prometaphase | 0.305807935 | 1.356057756 | 0.087549584 |
| centrosomes | 0.300300551 | 1.331853174 | 0.091430094 |
| hypofunction | 0.300201844 | 1.33141936 | 0.091594505 |
| hepatectomy | 0.298390233 | 1.323457454 | 0.092917689 |
| gabaergic | 0.294481732 | 1.306279855 | 0.095776279 |
| dendrites | 0.294340259 | 1.305658092 | 0.095776279 |
| spines | 0.29393742 | 1.303887638 | 0.096116791 |
| globus | 0.292710959 | 1.298497426 | 0.097143667 |
| enterococcus | 0.290049354 | 1.286799853 | 0.099047144 |
| somatosensory | 0.289774838 | 1.285593372 | 0.099221531 |
| oligopeptides | 0.28973268 | 1.28540809 | 0.099396142 |
| high-frequency | 0.288022148 | 1.277890415 | 0.100624713 |
| maze | 0.287888768 | 1.277304221 | 0.100801124 |
| evoked | 0.287336288 | 1.274876107 | 0.101154621 |
| mitoses | 0.286491536 | 1.271163479 | 0.101864324 |
| parvalbumin | 0.283072083 | 1.256135213 | 0.104557985 |
| ethyl | 0.282929899 | 1.255510325 | 0.104557985 |
| polyploid | 0.280995288 | 1.247007841 | 0.106198749 |
| neuroplasticity | 0.280578228 | 1.245174891 | 0.106565875 |
| entry | 0.280248204 | 1.243724456 | 0.106749781 |
| threonine | 0.279954917 | 1.242435479 | 0.107118281 |
| quantal | 0.278954731 | 1.238039729 | 0.10785803 |
