# Supplementary material for: Textrous!: Extracting Semantic Textual Meaning from Gene Sets
Source: PLoS One. 2013 Apr 30;8(4):e62665. doi: 10.1371/journal.pone.0062665 (PMC3639949; doi:10.1371/journal.pone.0062665)
Supplement: Table S14 — Textrous! noun-phrase output from hPTH (1–34) calvarial bone transcription responses in wild-type mice. The data indicated in the table consists of the Cosine similarity scores for the most strongly associated noun-phrases linked to the top 10 most significantly-associated words extracted by Textrous! from the hPTH (1–134)-induced transcriptome data. (DOC) [file pone.0062665.s015.doc]

**Table S14. *Textrous!* noun-phrase output from hPTH (1-34) calvarial bone transcription responses in wild-type mice.** The data indicated in the table consists of the Cosine similarity scores for the most strongly associated noun-phrases linked to the top 10 most significantly-associated words extracted by *Textrous!* from the hPTH (1-134)-induced transcriptome data.

| **Word** | **Noun-Phrase** | **Cosine Similarity** |
| --- | --- | --- |
| adherens | *fascia adherens* | *0.61666572* |
|  | *zonula adherens* | *0.600638626* |
|  | *full adherens* | *0.584241849* |
|  | *zonula adherens integrity* | *0.552635425* |
|  | *zonula adherens assembly* | *0.49321546* |
|  | *disorganized zona adherens* | *0.477660413* |
|  | *zona adherens* | *0.476461362* |
|  | *epithelial adherens junctions* | *0.473571649* |
|  | *adherens junction formation* | *0.443658382* |
|  | *adherens junctions* | *0.436198996* |
|  | *adherens junction* | *0.435294505* |
|  | *abnormal adherens junctions* | *0.43439758* |
|  | *typical adherens junctions* | *0.433886815* |
|  | *adherens junction molecules* | *0.42644019* |
|  | *adherens junction maturation* | *0.423531707* |
|  | *adherens junction components* | *0.420594247* |
|  | *adherens junction ring* | *0.413241433* |
|  | *adherens junction assembly* | *0.40000835* |
|  | *adherens junction complexes* | *0.396270325* |
|  | *adherens junction complex* | *0.38661882* |
|  | *intercellular adherens junctions* | *0.375410716* |
|  | *intercellular adherens junction components* | *0.349966259* |
|  | *adherens junction protein* | *0.331532311* |
|  | *major adherens junction protein* | *0.322477553* |
|  | *photoreceptor adherens junction* | *0.197918572* |
|  |  |  |
| alpha-catenin | *surface epithelium alpha-catenin* | *0.342050632* |
|  | *human alpha-catenin* | *0.230644107* |
|  | *alpha-catenin expression* | *0.203910921* |
|  | *alpha-catenin genes* | *0.106469189* |
|  | *alpha-catenin dimerization domain* | *0.105654953* |
|  | *alpha-catenin protein ablation* | *0.103198395* |
|  | *alpha-catenin null keratinocytes* | *0.100898551* |
|  | *alpha-catenin binding partners* | *0.08183376* |
|  | *cytoplasmic proteins alpha-catenin* | *0.062620855* |
|  |  |  |
| gamma-catenin | *gamma-catenin transformation* | *0.138700751* |
|  |  |  |
| catenin | *beta catenin* | *0.441924174* |
|  |  |  |
| tilt | *head tilt mutation* | *-0.054160335* |
|  |  |  |
| plakoglobin | *plakoglobin associates* | *0.388180199* |
|  | *junction plakoglobin* | *0.369269758* |
|  | *bovine plakoglobin* | *0.240324054* |
|  | *plakoglobin knockout* | *0.224371284* |
|  | *plakoglobin mutant mouse embryos* | *0.154018633* |
|  | *plakoglobin mrna* | *0.142892051* |
|  | *mutant plakoglobin* | *0.089876303* |
|  | *human plakoglobin cdna* | *0.067302321* |
|  |  |  |
| n-cadherin | *human n-cadherin* | *0.493375223* |
|  | *n-cadherin complexes* | *0.471363323* |
|  | *defective n-cadherin processing* | *0.463880864* |
|  | *cell adhesion molecule n-cadherin* | *0.322767797* |
|  |  |  |
| cadherin | *adherence junction component cadherin* | *0.54521798* |
|  | *extracellular cadherin repeat* | *0.50438745* |
|  | *epithelial cadherin* | *0.493705629* |
|  | *cadherin superfamily* | *0.492725117* |
|  | *atypical cadherin* | *0.487899359* |
|  | *cadherin organization* | *0.487450748* |
|  | *placental cadherin* | *0.487386372* |
|  | *representative classical cadherin* | *0.487251102* |
|  | *extracellular cadherin domains* | *0.485438869* |
|  | *common cadherin* | *0.48318291* |
|  | *cadherin repeats* | *0.482495842* |
|  | *contiguous cadherin repeats* | *0.481964445* |
|  | *cadherin stereocilia* | *0.479347441* |
|  | *cadherin motifs* | *0.478867076* |
|  | *cadherin expression* | *0.476272331* |
|  | *cadherin cytoplasmic region* | *0.470130524* |
|  | *cadherin adhesive complexes* | *0.466555406* |
|  | *type ii classic cadherin* | *0.465913388* |
|  | *cadherin domains* | *0.464833124* |
|  | *cadherin motif* | *0.464693628* |
|  | *cadherin sequences* | *0.463140403* |
|  | *cadherin extracellular domain* | *0.460313718* |
|  | *cadherin substrates* | *0.459750337* |
|  | *neural cadherin* | *0.459402696* |
|  | *cadherin complex* | *0.456923117* |
|  | *mouse epithelial cadherin* | *0.456220071* |
|  | *functional cadherin complex* | *0.447227497* |
|  | *epithelial cell cadherin* | *0.447032815* |
|  | *cadherin genes* | *0.438095249* |
|  | *cadherin domain* | *0.436547881* |
|  | *muscle cadherin* | *0.435782956* |
|  | *cadherin class* | *0.435162172* |
|  | *mature cadherin proteins* | *0.420324438* |
|  | *cadherin cytoplasmic domain* | *0.415775677* |
|  | *calcium-dependent cadherin cell adhesion molecules* | *0.299368585* |
|  | *vascular endothelial cadherin* | *0.280611252* |
|  | *vascular endothelial cell cadherin* | *0.267325561* |
|  |  |  |
| catenins |  |  |
|  |  |  |
| cadherins | *classic cadherins* | *0.473476921* |
|  | *classical cadherins* | *0.469477642* |
|  | *typical cadherins* | *0.46713094* |
|  | *desmosomal cadherins* | *0.46133384* |
|  | *neural cadherins* | *0.413361208* |
|  | *retinal cadherins* | *0.232850257* |
