# Supplementary material for: Textrous!: Extracting Semantic Textual Meaning from Gene Sets
Source: PLoS One. 2013 Apr 30;8(4):e62665. doi: 10.1371/journal.pone.0062665 (PMC3639949; doi:10.1371/journal.pone.0062665)
Supplement: Table S15 — Textrous! noun-phrase output from bPTH (7–34) parathyroid hormone variant-treatment of calvarial bone transcription responses in wild-type mice. The data indicated in the table consists of the Cosine similarity scores for the most strongly associated noun-phrases linked to the top 10 most significantly-associated words linked to the bPTH (7–34)-induced transcriptome data. (DOC) [file pone.0062665.s016.doc]

**Table S15. *Textrous!* noun-phrase output from bPTH (7-34) parathyroid hormone variant-treatment of calvarial bone transcription responses in wild-type mice.** The data indicated in the table consists of the Cosine similarity scores for the most strongly associated noun-phrases linked to the top 10 most significantly-associated words linked to the bPTH (7-34)-induced transcriptome data.

| **Word** | **Noun-Phrase** | **Cosine Similarity** |
| --- | --- | --- |
| cyclin | *cyclin levels* | *0.66371713* |
|  | *decreased cyclin* | *0.662191747* |
|  | *cyclin es* | *0.659314001* |
|  | *higher cyclin* | *0.658521539* |
|  | *reduced cyclin* | *0.657472563* |
|  | *reconstituted cyclin* | *0.656980464* |
|  | *native cyclin* | *0.656754436* |
|  | *cyclin boxes* | *0.655435127* |
|  | *major cyclin* | *0.65542185* |
|  | *cyclin destruction* | *0.654959836* |
|  | *cyclin partner* | *0.654185368* |
|  | *regulating cyclin* | *0.653119074* |
|  | *multiple cyclin* | *0.652722966* |
|  | *canonical cyclin fold* | *0.652068231* |
|  | *endogenous cyclin* | *0.650957763* |
|  | *central cyclin box* | *0.650349251* |
|  | *human cyclin* | *0.649556678* |
|  | *cyclin box* | *0.649144769* |
|  | *cyclin consensus sequence* | *0.648586209* |
|  | *homologous cyclin* | *0.648138633* |
|  | *cyclin expression* | *0.643448255* |
|  | *s-phase cyclin complexes* | *0.642234183* |
|  | *reduced cyclin expression* | *0.641874503* |
|  | *cyclin domains* | *0.641781578* |
|  | *cyclin box consensus sequence* | *0.640231622* |
|  | *functional cyclin box* | *0.63847758* |
|  | *cyclin box motif* | *0.637322428* |
|  | *cyclin homology region* | *0.633960352* |
|  | *mitotic cyclin* | *0.632749545* |
|  | *mouse cyclin* | *0.63194441* |
|  | *human b-type cyclin* | *0.628819571* |
|  | *n-terminal cyclin box* | *0.628491247* |
|  | *conserved cyclin box region* | *0.626005672* |
|  | *n-terminal cyclin a-binding motif* | *0.62238905* |
|  | *mutant cyclin* | *0.621733467* |
|  | *cyclin box domain* | *0.617815716* |
|  | *defective cyclin degradation* | *0.607126301* |
|  | *cyclin d-dependent kinases* | *0.605600513* |
|  | *conserved n-terminal cyclin box region* | *0.600726112* |
|  | *cyclin d-dependent phosphorylation* | *0.563966331* |
|  | *cyclin d-dependent kinase* | *0.49774016* |
|  |  |  |
| cyclins | *a-type cyclins* | *0.592579342* |
|  | *c-type cyclins* | *0.572820475* |
|  | *vertebrate cyclins* | *0.527124346* |
|  | *human cyclins* | *0.466958213* |
|  | *b-type cyclins* | *0.366348755* |
|  |  |  |
| cyclin-dependent | *cyclin-dependent cell cycle regulation* | *0.441380875* |
|  | *cyclin-dependent kinases* | *0.322065441* |
|  | *human cyclin-dependent kinases* | *0.29983041* |
|  | *cyclin-dependent kinase inhibitor* | *0.271944758* |
|  | *cyclin-dependent protein kinases* | *0.239075706* |
|  | *cyclin-dependent kinase inhibitors* | *0.218058305* |
|  | *cyclin-dependent kinase complexes* | *0.206220105* |
|  | *cyclin-dependent kinase sites* | *0.203292399* |
|  | *cyclin-dependent kinase* | *0.203231293* |
|  | *cyclin-dependent kinase activity* | *0.199156612* |
|  | *critical cyclin-dependent kinase substrates* | *0.19103248* |
|  | *potential cyclin-dependent kinase phosphorylation site* | *0.171020294* |
|  | *cyclin-dependent kinase phosphorylation* | *0.170371599* |
|  | *cyclin-dependent kinase activation* | *0.158338564* |
|  |  |  |
| m-phase | *m-phase progression* | *0.411072036* |
|  | *m-phase extracts* | *0.359639146* |
|  | *m-phase events* | *0.319873332* |
|  | *m-phase checkpoints* | *0.297115573* |
|  | *m-phase abnormalities* | *0.263274382* |
|  | *xenopus m-phase* | *0.156744912* |
|  | *m-phase cells* | *0.12663011* |
|  | *m-phase cytosol* | *0.111439443* |
|  | *m-phase promoting factor* | *0.051586589* |
|  | *m-phase kinases* | *-0.008828006* |
|  |  |  |
| cdk | *cdk inhibitor* | *0.566906191* |
|  | *cdk inhibitors* | *0.516755961* |
|  | *cdk module* | *0.495010382* |
|  | *normal cdk control mechanisms* | *0.456166812* |
|  | *cdk consensus sites* | *0.445710169* |
|  | *partial cdk activity* | *0.420726803* |
|  | *cdk activity* | *0.420373104* |
|  | *human cdk* | *0.390354083* |
|  | *cdk activator* | *0.358541005* |
|  | *cdk interaction* | *0.304173909* |
|  | *downstream cdk substrate* | *0.287081685* |
|  | *conserved cdk motif* | *0.277459848* |
|  | *cdk pathway* | *0.276675034* |
|  | *vivo cdk substrate* | *0.252348119* |
|  | *cdk regulatory signals* | *0.220355955* |
|  | *cdk phosphorylation* | *0.199859931* |
|  | *cdk phosphorylation sites* | *0.196340671* |
|  | *cdk activation* | *0.180706006* |
|  | *n-terminal cdk inhibitory domain* | *0.174269855* |
|  |  |  |
| n-methyl-d-aspartic |  |  |
|  |  |  |
| glutamatergic | *glutamatergic excitotoxin kainate* | *0.480890002* |
|  | *glutamatergic transmission* | *0.46524577* |
|  | *glutamatergic synapses* | *0.464237408* |
|  | *glutamatergic hypofunction* | *0.461779216* |
|  | *glutamatergic tone* | *0.460596596* |
|  | *intrinsic glutamatergic connections* | *0.456257036* |
|  | *excitatory glutamatergic synapses* | *0.453002779* |
|  | *glutamatergic terminals* | *0.441313172* |
|  | *glutamatergic synaptic transmission* | *0.438437958* |
|  | *excitatory glutamatergic* | *0.43241201* |
|  | *glutamatergic presynaptic short-term plasticity* | *0.430233895* |
|  | *forebrain glutamatergic* | *0.428675617* |
|  | *glutamatergic system* | *0.424420237* |
|  | *forebrain glutamatergic circuits* | *0.421937253* |
|  | *glutamatergic synaptic currents* | *0.416986812* |
|  | *glutamatergic neurotransmission* | *0.414367927* |
|  | *presynaptic glutamatergic nerve endings* | *0.409891863* |
|  | *postnatal glutamatergic synapse development* | *0.399536984* |
|  | *excitatory glutamatergic neurons* | *0.398503925* |
|  | *glutamatergic hippocampal neurons* | *0.380245152* |
|  | *glutamatergic neurons* | *0.366568123* |
|  | *rat glutamatergic neurons* | *0.353221115* |
|  | *increased glutamatergic spontaneous release events* | *0.344634824* |
|  | *cortical glutamatergic neurons* | *0.340709706* |
|  | *glutamatergic pathways* | *0.29128119* |
|  | *glutamatergic phenotype* | *0.285817699* |
|  | *glutamatergic cells* | *0.264869652* |
|  |  |  |
| nmda | *nmda receptor-mediated excitatory postsynaptic potentials* | *0.47307394* |
|  | *nmda toxicity* | *0.45246107* |
|  | *nmda receptor-mediated neurotransmission* | *0.451100887* |
|  | *nmda currents* | *0.445105997* |
|  | *nmda receptor-mediated glutamate toxicity* | *0.442030491* |
|  | *nmda receptor-mediated synaptic currents* | *0.442014675* |
|  | *nmda responses* | *0.439054915* |
|  | *nmda receptor-mediated field responses* | *0.434559647* |
|  | *nmda agonist* | *0.40379383* |
|  | *nmda glutamate receptors* | *0.387994045* |
|  | *nmda synaptic receptors* | *0.369699236* |
|  | *synaptic nmda receptors* | *0.369699236* |
|  | *postsynaptic nmda receptors* | *0.354639087* |
|  | *hippocampal nmda receptors* | *0.344239597* |
|  | *nmda receptors* | *0.308665993* |
|  | *blocking nmda receptors* | *0.305989733* |
|  | *nmda channels* | *0.297879077* |
|  | *competitive nmda antagonist* | *0.292095252* |
|  | *functional nmda receptors* | *0.291765312* |
|  |  |  |
| postsynaptic | *nmda receptor-mediated excitatory postsynaptic potentials* | *0.47307394* |
|  | *postsynaptic layer* | *0.465517399* |
|  | *postsynaptic decrease* | *0.462537237* |
|  | *postsynaptic terminals* | *0.454102393* |
|  | *excitatory postsynaptic sites* | *0.453655589* |
|  | *postsynaptic terminal* | *0.450993122* |
|  | *postsynaptic spikes* | *0.450431691* |
|  | *long-lasting inhibitory postsynaptic potentials* | *0.45001817* |
|  | *postsynaptic pool* | *0.448930104* |
|  | *postsynaptic specialization* | *0.447397623* |
|  | *postsynaptic densities* | *0.445244822* |
|  | *postsynaptic plasticity* | *0.444883969* |
|  | *postsynaptic side* | *0.444359533* |
|  | *postsynaptic apparatus* | *0.443956075* |
|  | *postsynaptic events* | *0.443882894* |
|  | *postsynaptic sites* | *0.441941611* |
|  | *postsynaptic markers* | *0.441494914* |
|  | *postsynaptic inhibition* | *0.4414855* |
|  | *postsynaptic site* | *0.439988951* |
|  | *postsynaptic maturation* | *0.439419901* |
|  | *postsynaptic reception apparatus* | *0.438066592* |
|  | *postsynaptic action* | *0.435061021* |
|  | *evoked postsynaptic potentials* | *0.434841778* |
|  | *postsynaptic density* | *0.434710928* |
|  | *postsynaptic density fractions* | *0.432111239* |
|  | *postsynaptic density preparations* | *0.429080954* |
|  | *postsynaptic density fraction* | *0.427593969* |
|  | *postsynaptic glycine* | *0.427171762* |
|  | *postsynaptic region* | *0.424766732* |
|  | *postsynaptic components* | *0.423911157* |
|  | *postsynaptic structures* | *0.422776581* |
|  | *postsynaptic regions* | *0.422332044* |
|  | *postsynaptic scaffolding* | *0.422215785* |
|  | *postsynaptic role* | *0.422157337* |
|  | *miniature excitatory postsynaptic currents* | *0.417936066* |
|  | *excitatory postsynaptic currents* | *0.417524313* |
|  | *kainate receptor-mediated excitatory postsynaptic currents* | *0.416424305* |
|  | *postsynaptic neuron* | *0.414613455* |
|  | *gaba-a receptor-mediated postsynaptic inhibition* | *0.413478156* |
|  | *excitatory postsynaptic current properties* | *0.413148322* |
|  | *postsynaptic elements* | *0.409918152* |
|  | *postsynaptic activity* | *0.406487348* |
|  | *spontaneous gabaergic inhibitory postsynaptic currents* | *0.405922213* |
|  | *miniature excitatory postsynaptic current frequency* | *0.396633059* |
|  | *postsynaptic current* | *0.395933156* |
|  | *postsynaptic density scaffold* | *0.393668253* |
|  | *postsynaptic compartment* | *0.39239065* |
|  | *postsynaptic membranes* | *0.391182533* |
|  | *spontaneous miniature excitatory postsynaptic current frequency* | *0.388965566* |
|  | *postsynaptic calcium* | *0.387052595* |
|  | *spontaneous inhibitory postsynaptic responses* | *0.384982513* |
|  | *postsynaptic currents* | *0.383990801* |
|  | *inhibitory postsynaptic current frequency* | *0.383644235* |
|  | *postsynaptic responses* | *0.383189805* |
|  | *postsynaptic molecules* | *0.382296521* |
|  | *postsynaptic neurons* | *0.381579258* |
|  | *postsynaptic spiral ganglion neurons* | *0.378030007* |
|  | *postsynaptic target* | *0.377133916* |
|  | *neuronal postsynaptic densities* | *0.376575852* |
|  | *postsynaptic calcium transients* | *0.37364068* |
|  | *postsynaptic expression* | *0.37234487* |
|  | *postsynaptic differentiation* | *0.367545715* |
|  | *postsynaptic localization* | *0.365636437* |
|  | *postsynaptic density complex* | *0.364183119* |
|  | *central postsynaptic hearing impairment* | *0.356408472* |
|  | *postsynaptic nmda receptors* | *0.354639087* |
|  | *postsynaptic enzymes* | *0.347730141* |
|  | *postsynaptic central nervous system neurons* | *0.340112069* |
|  | *postsynaptic congenital myasthenic syndromes* | *0.338724891* |
|  | *postsynaptic spectrin cytoskeletons* | *0.333942736* |
|  | *postsynaptic dopaminergic neurons* | *0.330680056* |
|  | *postsynaptic cytoskeleton* | *0.330286647* |
|  | *postsynaptic trafficking* | *0.326218386* |
|  | *postsynaptic surface expression* | *0.325981781* |
|  | *postsynaptic clathrin-coated vesicles* | *0.325429918* |
|  | *postsynaptic cells* | *0.323378972* |
|  | *postsynaptic neuromuscular junction* | *0.320486141* |
|  | *postsynaptic calcium ion sensor* | *0.3198639* |
|  | *postsynaptic ampa receptors* | *0.316917247* |
|  | *functional postsynaptic response* | *0.316675244* |
|  | *postsynaptic endocytic events* | *0.314315539* |
|  | *postsynaptic plasma membranes* | *0.295343682* |
|  | *postsynaptic lipid raft fractions* | *0.293049302* |
|  | *specialized postsynaptic endocytic mechanism* | *0.27909233* |
|  | *postsynaptic density proteins* | *0.253199829* |
|  | *postsynaptic proteins* | *0.242183929* |
|  | *nicotinic fast excitatory postsynaptic currents* | *0.241021333* |
|  | *postsynaptic junctions* | *0.240229295* |
|  | *postsynaptic domain* | *0.239929928* |
|  | *inhibitory postsynaptic membrane formation* | *0.224669721* |
|  | *electric organ postsynaptic membrane* | *0.211636792* |
|  | *postsynaptic protein sorting* | *0.207346802* |
|  | *postsynaptic membrane* | *0.207093278* |
|  | *postsynaptic ephb receptors* | *0.205388856* |
|  | *postsynaptic receptors* | *0.205092182* |
|  | *postsynaptic congenital myasthenic syndrome* | *0.197620862* |
|  | *postsynaptic calcium channels* | *0.162647921* |
|  | *postsynaptic membrane trafficking* | *0.136222772* |
|  | *postsynaptic adhesion* | *0.115844359* |
|  | *postsynaptic atp-sensitive potassium channels* | *0.076565677* |
|  |  |  |
| mitosis | *normal mitosis* | *0.451746699* |
|  | *terminal mitosis* | *0.445830924* |
|  | *increased mitosis* | *0.441125255* |
|  | *mitosis control* | *0.440553002* |
|  | *early mitosis* | *0.437496298* |
|  | *abnormal mitosis* | *0.421538342* |
|  | *aberrant mitosis* | *0.399203589* |
|  | *glial mitosis* | *0.390617606* |
|  | *cell mitosis* | *0.379656671* |
|  | *mitosis human* | *0.35982392* |
|  | *normal erythroid mitosis* | *0.320533961* |
|  | *cells exit mitosis* | *0.305692699* |
